# Cost-Effectiveness of Electronic Patient-Reported Outcome Measure Interventions in Cancer: Systematic Review and Parameter Extraction for Economic Modeling

**DOI:** 10.2196/100505

**Published:** 2026-09-11

**Authors:** Marek Atter, Alistair Bullen, Kathrin Cresswell, Peter S Hall

**Affiliations:** 1 College of Medicine & Veterinary Medicine University of Edinburgh Edinburgh, Scotland United Kingdom; 2 Yunus Centre Glasgow Caledonian University Glasgow, Scotland United Kingdom

**Keywords:** digital technology, health care economics and organizations, neoplasms, patient-reported outcome measures, systematic review

## Abstract

**Background:**

Complex digital interventions that integrate electronic patient-reported outcome measures (ePROM) into clinical practice in cancer have the potential to improve quality of life, increase survival, and reduce health resource use and costs. Such systems can help patients with cancer self-manage chemotherapy symptoms, reduce clinicians’ workloads through automated decision support, and resolve problems earlier. However, more research on the cost-effectiveness of ePROM monitoring is needed.

**Objective:**

This paper comprises two complementary components: (1) a systematic literature review summarizing and evaluating the quantitative and qualitative evidence related to the cost-effectiveness of ePROM monitoring and (2) a health economic model parameter extraction. We also conducted supplementary targeted searches and scoping to provide context to our findings.

**Methods:**

We searched Ovid (including MEDLINE and Embase), Scopus, and the International Health Technology Assessment Database for original English-language papers published on or before March 2025 using search strings that combined terms related to ePROMs, health economics, and cancer/oncology. We included papers reporting health economic–related outcomes for ePROM interventions designed for adult cancer populations and excluded screening tools and conference abstracts.

**Results:**

We included 34 publications from 27 unique studies and identified and analyzed 26 ePROM-integrated interventions within these. Most (23/26) of the included interventions explicitly described some form of alert handling and automated decision support based on remote ePROM monitoring. Of the 34 publications, 5 presented full cost-effectiveness analysis results, of which 3 were highly uncertain and lacked clear differences in costs and health outcomes between ePROMs and standard care; conversely, 2 presented strong evidence of cost-effectiveness due to quality-of-life improvements, reduced hospitalizations, and potentially more autonomy in health-related travel (eg, ePROM-monitored patients can drive or walk to the hospital instead of using taxis or ambulances). A further 5 publications reported partial health economic results (eg, cost-consequence and budget impact), of which 1 detected no difference in strategies; in contrast, 4 reported lower health resource use and costs of ePROMs, mainly due to hospitalization reductions. Overall, 12 of the 27 studies included a qualitative component but mostly focused on user experience and design-related themes; only 2 of these addressed economic-specific themes (eg, changes in workflow and resource use due to ePROM implementation and integration), indicating some potential for time saving due to ePROM monitoring.

**Conclusions:**

Some ePROM-integrated interventions demonstrated cost-effectiveness in cancer care, but the evidence base remains limited. Where evidence does exist, cost-effectiveness appears driven by reduced hospitalization and improved quality of life. Qualitative research within the included studies rarely addressed economic questions. We provide a detailed parameter extraction for use in future economic modeling and recommend research priorities, including quantitative mapping of ePROM symptom data onto health resource use patterns, and qualitative work exploring how ePROM implementation affects clinical workloads and patient-perspective costs.

## Introduction

### Rationale

Patient-reported outcome measures (PROMs) are data reported directly by patients without interpretation by health care professionals (HCPs) [1]. Electronic systems and mobile devices offer greater capabilities than paper-based data collection for integrating electronic patient-reported outcome measures (ePROMs) into routine care, supporting new health interventions such as automated guidance for patient self-management, real-time alerts, automated scheduling, and earlier adverse event monitoring [1]. These innovations have been shown to improve outcomes, including greater patient health-related quality of life (HRQoL) and lower rates of hospital admissions, albeit with mixed results necessitating further research [2-4].

Preliminary evidence from ePROM use in cancer points to statistically significant reductions in resource use (eg, emergency department visits) and the potential for HRQoL improvement and cost-effectiveness of online symptom monitoring [5,6]. This could be especially useful in facilitating patient-centered care in cancer, where there is an unmet need for addressing patients’ high symptom burden, loss of function, and emotional distress [7]. Patient-centered care refers to care focused on patient preferences, needs, and values, provided alongside tumor-centered care, which focuses on treating the disease [7]. However, despite the potential, implementing ePROMs is difficult in real-world practice, due to sociotechnical factors including intervention complexity (with a large number of interrelated technological, behavioral, and organizational components), integration with socio-organizational environments and work practices (eg, lack of adequate equipment and resources), and infrastructural or other technological characteristics (eg, lack of user-friendly ePROM integration into electronic medical records) [2,8-10].

Standard existing methods for cost-effectiveness analysis are insufficient in the analysis and modeling of ePROM interventions. First, ePROM-integrated programs are “complex” interventions in public health by the Medical Research Council’s definition, due mainly (but not limited) to the number of components involved and expertise/skills required [11]. For example, a patient may complete ePROMs on their tablet, after which a dedicated HCP must review symptom-triggered alerts, all while a software company maintains the ePROM-to-HCP action information transmission system and integrates it with existing hospital records; this complicates the appropriate selection and inclusion of variables within a health economic analysis and increases the number of assumptions needed for modeling. Second, health economic models often lack formal methods (eg, conceptual modeling) and could benefit from conducting qualitative research to inform model structure and assumptions; without these, a bloated set of assumptions due to ePROM complexity may imperil model validity [12].

As a result of these difficulties, health economic evaluations of ePROMs are scarce despite the potential impacts on HRQoL and health resource use (HRU). A systematic literature review (SLR) of interventions using PROMs to monitor symptoms in patients with cancer conducted by Lizán et al [13] has shown evidence of substantial improvements to survival (≥5 months gained per patient) and HRQoL, as well as reductions in HRU in emergency services and readmissions; however, it does not focus on digital interventions or remote monitoring or provide detailed parameter extraction for economic modeling [5,6]. Therefore, there is a need to research further economic literature related to complex digital interventions using ePROMs, their impact on costs and health outcomes, and the methods used to understand their cost-effectiveness.

### Objectives

This paper has a dual objective: (1) systematically summarizing existing economic evidence and (2) extracting granular health economic modeling parameters.

First, we aimed to assess and summarize the evidence of ePROM cost-effectiveness, as well as the challenges, potential methods, and improvements to practices when designing cost-effectiveness analyses of ePROM-based interventions in cancer care.

Second, we set out to inform the structure, parameters, and assumptions of an early health economic model evaluating the cost-effectiveness of using ePROMs in cancer care [7].

## Methods

### Protocol and Registration

The protocol for this SLR was produced following the PRISMA-P (Preferred Reporting Items for Systematic Review and Meta-Analysis Protocols) 2015 statement and was published on PROSPERO (CRD420251023447) [14,15].

### Eligibility Criteria

We included studies related to adults (aged ≥18 years) with a diagnosis of any form of cancer and excluded studies focused on pediatric (aged <18 years) patients. The interventions of interest included integrated elements of ePROMs or other digital tools that facilitate regular patient-clinic communication or real-time self-reporting and monitoring. We therefore excluded studies that used ePROMs solely for research purposes (ie, not integrated into the intervention) or as one-off screening tools. We included studies that reported outcomes from any of the following categories: (1) health economic outcomes, such as changes in quality-adjusted life years (QALYs), HRU, and/or costs; (2) mapping of ePROM outcomes onto validated health economic measures; and (3) qualitative processes and mechanisms by which ePROMs impact HRU patterns in cancer care. We rejected studies that did not report outcomes relevant to or useful in economic analyses. We accepted full-text English-language quantitative, qualitative, and mixed methods studies, including clinical trials (both randomized and nonrandomized), observational studies, literature reviews, implementation papers, and published guidelines. We rejected conference abstracts and proceedings, as well as nonscientific material (news reports, websites, etc).

### Information Sources

We searched publications on Scopus, the International Health Technology Assessment (HTA) Database (INAHTA), and Ovid, which comprises MEDLINE and Embase. The choice of databases included in the search strategy was modeled on a recent (2024) health economic SLR within the field of oncology published in *Pharmacoeconomics* [16].

### Search Strategy

The search strategy combined concepts relating to ePROMs, cost-effectiveness, and cancer into terms used and adapted for specific databases. The exact search strings used in each database are provided in the Multimedia Appendix 1.

### Study Records

The selection process started with 2 blinded independent reviewers (MA and AB) screening the titles and abstracts based on the eligibility criteria. The protocol stated that a third reviewer would resolve disagreements, but this was unnecessary as the two reviewers reached consensus following unblinding. We then retrieved records accepted at the screening stage and conducted a blinded, independent full-text screening by the same two reviewers. At this stage, we also conducted a secondary reference screening, whereby potentially useful papers referenced within the full-text review were added to the title and abstract screening.

Once we finalized a list of included entries, we extracted key data using a standardized collection form. Extracted information included study characteristics, type of intervention, HRU outcomes, cost-effectiveness results, and study quality. We also documented processes by which ePROMs changed HRU patterns and/or health outcomes and described methods and themes present within qualitative or mixed methods components. One researcher extracted the data (MA), while the other checked and verified the output (AB).

We set out to contact authors in cases where the existence of data relevant to this review was mentioned in a paper but not available in full, which we did in 2 cases. We asked researchers from the CAPRI (*Impact of a Monitoring Device for Patients With Cancer Treated Using Oral Therapeutics*) trial to provide absolute numbers of HRU events; however, the data were no longer available. We asked authors of the PHONEME/Interaktor report to provide complete versions of the partial HRU event rate formulae equations, which they were able to provide. We thank both authors for their responses and help.

We stored and screened records using dedicated literature review software (Rayyan; Rayyan Systems Inc).

### Risk of Bias and Reporting Quality

We assessed risk of bias (RoB) and reporting quality using appraisal tools appropriate for the study design, including the Cochrane RoB tool for clinical trials, CHEERS (Consolidated Health Economic Evaluation Reporting Standards) for economic evaluations, and PRISMA (Preferred Reporting Items for Systematic Reviews and Meta-Analyses) for SLRs [17-19].

We did not use a formal assessment of RoB due to missing evidence (eg, by using the ROB-ME tool), as we did not plan to conduct a meta-analysis [20]. Instead, we summarized possible publication bias by reviewing whether prespecified protocols and analysis plans preceded published results.

### Data Synthesis

We summarized the SLR results with an overview of the types and characteristics of the included studies and the identified interventions. We described the approaches to designing ePROM-integrated systems and their impact on health economic measures and cost-effectiveness. We also summarized the qualitative components of included studies, assessed their usefulness to the subject of our review, and discussed implications for economic modeling. This paper includes a detailed modeling parameter table along with guidance to help future modelers hoping to simulate the cost-effectiveness of ePROM monitoring in cancer.

### Framework for Model Parameter Extraction

**Figure 1:**
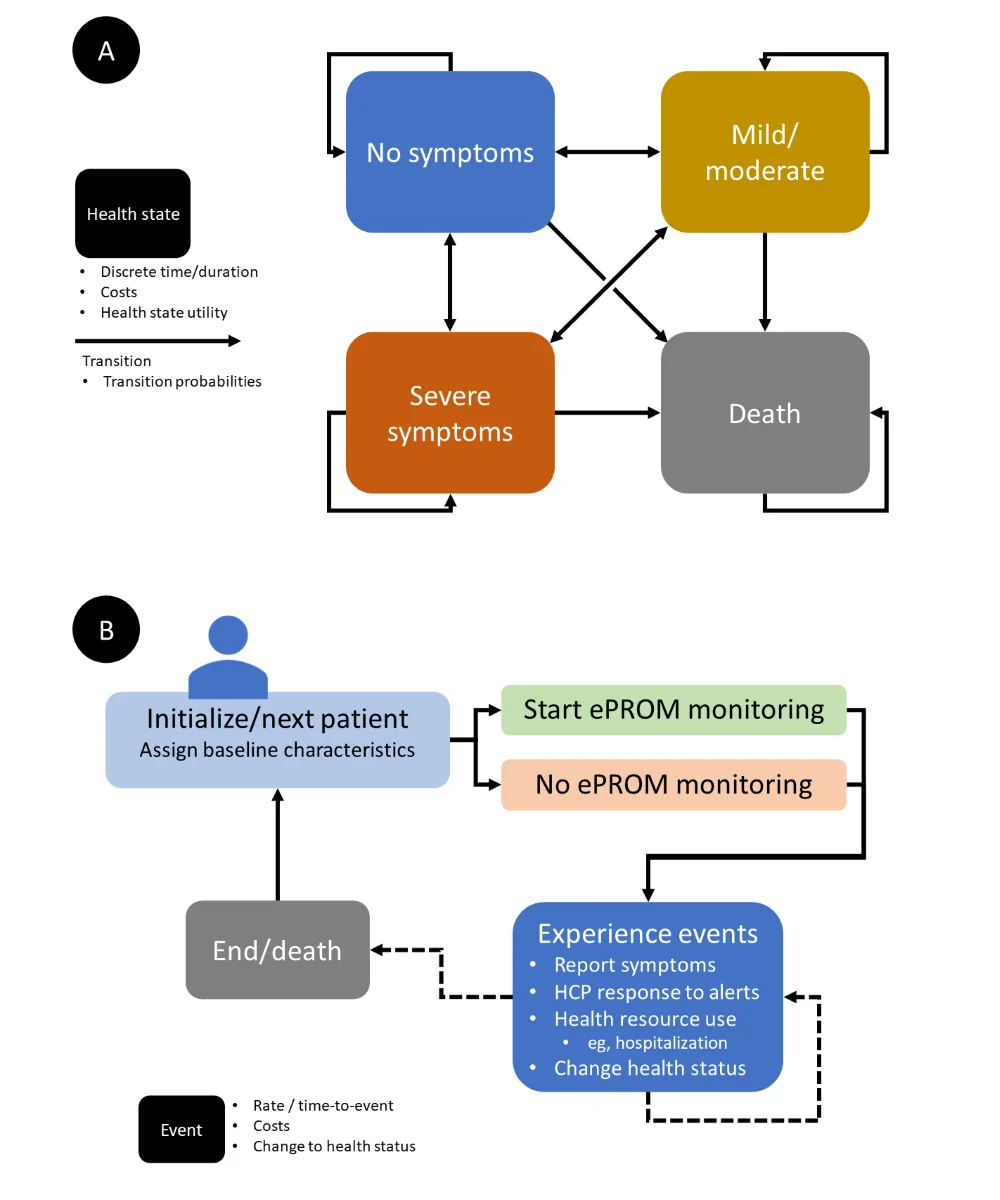
Potential health economic model diagrams for electronic patient-reported outcome measure (ePROM) interventions. HCP: health care professional.

To help structure the modeling parameter extraction, we defined a health economic model structure appropriate to the topic. Health economic models usually take one of two approaches: cohort (Markov chain) models with discrete time cycles, and patient-level discrete event simulations, presented in Figures 1A and 1B, respectively [21]. Commonly published empirical results are rarely suitably matched to a model’s structure. Many measures can be recalculated and converted with the right assumptions to suit the model’s needs [22]. Both cases require (1) knowledge of health state utilities to calculate QALYs and (2) rates, frequencies, and/or quantities of health resource consumption to calculate costs. Since costs per health resource unit are usually available in national databases (eg, the United Kingdom’s National Cost Collection), this review focused on extracting granular HRU data rather than composite costs [23]. Intervention-specific costs (eg, one-off setup costs and long-term maintenance of ePROM web applications), however, are needed to calculate differences between treatment strategies.

Due to the nature of ePROMs, a blended or hybrid approach to modeling may be necessary. Distinct self-reported symptoms could be considered health states in a Markov model. However, they are nonmutually exclusive, and memory of prior symptoms may influence disease or HRU trajectories, potentially necessitating a discrete event simulation approach. A hybrid framework, such as the Discrete-Integrated Condition Event, may help capture the complexities of tracking (and responding to) multiple symptoms in parallel [24].

Consequently, we sought to extract data in 5 categories: HRU event rates and rate ratios, health state utilities, alert handling probabilities, intervention costs, and relationships between ePROM scores and economic outcomes. For certainty assessment, we aimed to report SEs and CIs alongside extracted point estimates; medians and IQRs are less useful for modeling. We assumed studies would be too heterogeneous for a meta-analysis and instead prioritized granular parameter reporting conducive to filtering and tailoring for future scenario and sensitivity analyses.

### Reporting Checklist

We have provided a completed PRISMA 2020 checklist for this review in the Multimedia Appendix 2 [19].

### Meta-Biases and Confidence in Cumulative Evidence

Meta-biases and confidence in cumulative evidence were reported in line with the Grading of Recommendations, Assessment, Development and Evaluation (GRADE) framework [25].

## Results

### Study Selection

We conducted the database searches on March 30, 2025, yielding 1197 records (649 from Ovid, 541 from Scopus, and 7 from INAHTA). Of the 1197 records, 399 were automatically deduplicated using Systematic Research Accelerator software [26]. We uploaded 798 records to the Rayyan platform, from which a further 29 duplicates were removed manually through Rayyan’s assisted duplicate screening functionality [27]. Through manual (cross-reference) citation searching, 10 additional records were identified and added to Rayyan, yielding a total of 780 deduplicated records to be screened. From these, we excluded 706 after title and abstract screening. From the remaining 74 records, we retrieved and assessed all publications for eligibility, from which most (n=29) were excluded due to a wrong publication type (eg, Saria and Kesari [28]), 6 did not report any outcomes related to the cost-effectiveness or HRU of ePROM interventions (eg, Brunelli et al [29]), and 5 were judged to report on interventions that did not integrate or rely on ePROM processing or remote monitoring (eg, van den Berg et al [30]). The final analysis data extraction thus included 34 publications from 27 studies, with 5 from the eRAPID study and 2 each from the Sentinel, CAPRI, and Symptom Tracking and Reporting (STAR) studies [4,6,31-39].

**Figure 2:**
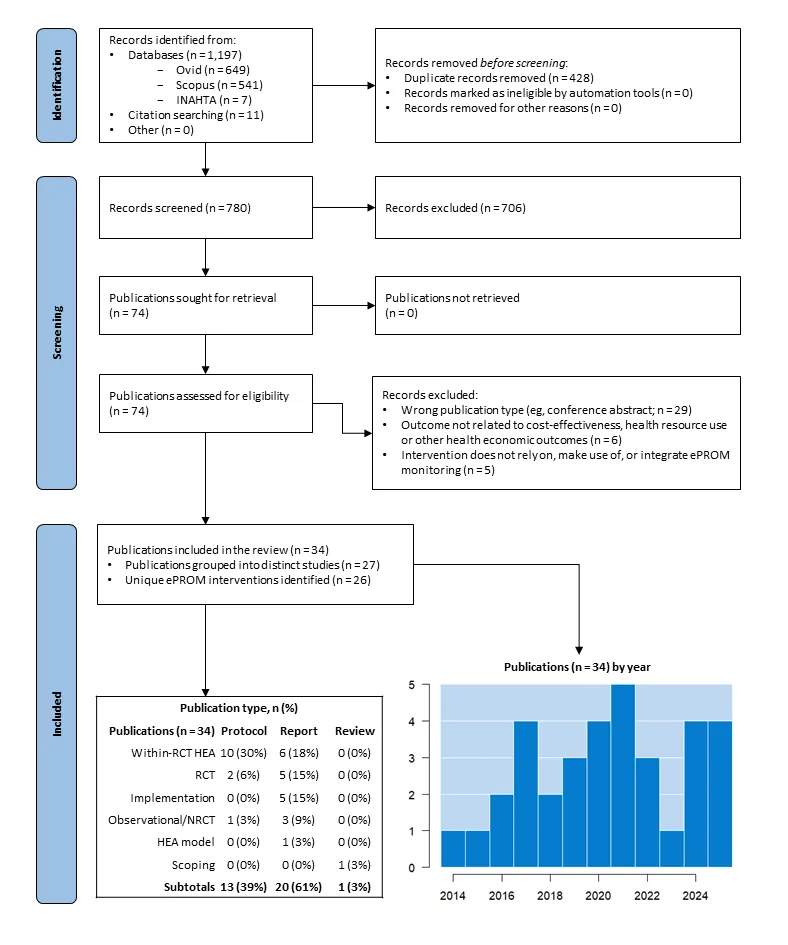
PRISMA (Preferred Reporting Items for Systematic Reviews and Meta-Analyses) diagram. ePROM: electronic patient-reported outcome measure; HEA: health economic evaluation; INAHTA: International Health Technology Assessment Database; NRCT: nonrandomized controlled trial; RCT: randomized controlled trial.

The PRISMA diagram in Figure 2 summarizes the search process [19]. In addition to the PRISMA flow, Figure 2 also presents a breakdown of publications by study type, publication type, and year of publication. The 34 included publications comprise 13 protocols of planned studies, 20 reports of completed study analyses, and 1 scoping review. The most common type of study reported in the included publications was a randomized controlled trial (RCT) with a health economic evaluation (HEA) component, mostly in the form of a cost-effectiveness analysis. These were followed by non-HEA RCTs that nevertheless reported important information about HRU (thus qualifying for inclusion), as well as implementation/feasibility or observational studies and non-RCTs. The years of publication range from 2014 to 2025, with a clear upward trend, peaking in 2021 at 5 entries, followed by a downturn in 2022-2023 in the aftermath of the COVID-19 pandemic, then reversed by a rebound of 4 publications in 2024 and Q1 of 2025.

### Study Characteristics

#### Overview

Within the 27 included studies, we identified 26 unique ePROM-integrated/self-monitoring intervention programs (either developed, planned, or simulated). The key characteristics of these interventions are (1) the country/setting, (2) the intended users (by one or multiple cancer types), (3) the ePROM tools or frameworks embedded in the intervention design, and (4) the symptoms monitored by a particular intervention. Table 1 displays a snapshot of the most commonly identified characteristics of each intervention, listed in order of the number and percentage of interventions with a particular characteristic. The following properties are non–mutually exclusive, as a particular ePROM program may have been developed for multiple countries and cancer populations, and/or use a combination of different ePROM tools, etc. A representative median ePROM intervention would be developed in a single country and use 1-2 validated ePROM tools to monitor around 6-7 symptoms. However, the symptom lists extracted from the studies may be incomplete and therefore undercounted. Most interventions catered to a patient population characterized either by a single cancer site (eg, breast) or by an umbrella term (eg, all cancers or solid cancers), but some interventions catered to up to 4 distinct cancer groups.

**Table 1:** Most common characteristics present by number of included ePROMa-integrated interventions (n=26).

Characteristic		Value
Countries/settings, n (%)		
	Netherlands	5 (19)
	United Kingdom	4 (15)
	Australia	3 (12)
	United States	3 (12)
	Italy	2 (8)
	Norway	2 (8)
	Ireland	2 (8)
	Greece	2 (8)
	Other	10 (38)
	N/A^b^	0 (0)
Number of countries/settings per ePROM intervention, median (range)		1 (1-5)
Cancer population, n (%)		
	Breast	9 (35)
	Colorectal	6 (23)
	Lung	4 (15)
	Prostate	4 (15)
	All (solid)	3 (12)
	Lymphoma	3 (12)
	Gynecologic	2 (8)
	All	2 (8)
	Other	5 (19)
	N/A	0 (0)
Number of cancer populations per ePROM intervention, median (range)		1 (1-4)
ePROM tools/frameworks, n (%)		
	Novel tools/frameworks	7 (27)
	PRO-CTCAE^c^ [40]	5 (19)
	EORTC QLQ-C30^d^ [41]	4 (15)
	Distress Thermometer [42]	3 (12)
	ESAS^e^ [43]	3 (12)
	PROMIS^f^ [44]	2 (8)
	BREAST-Q^g^ [45]	2 (8)
	ASyMS^h^ [46]	2 (8)
	Other	14 (54)
	N/A	1 (4)
Number of ePROM tools/frameworks per ePROM intervention, median (range)		1.5 (1-7)
Symptoms monitored, n (%)		
	Fatigue	14 (54)
	Pain	13 (50)
	Nausea	11 (42)
	Vomiting	7 (27)
	Constipation	7 (27)
	Appetite loss	7 (27)
	Anxiety/depression	7 (27)
	Diarrhea	6 (23)
	Other	26 (100)
	N/A	7 (27)
Number of symptoms monitored per ePROM intervention, median (range)		6.5 (1-18)

^a^ePROM: electronic patient-reported outcome measure.

^b^N/A: not available.

^c^PRO-CTCAE: Patient-Reported Outcomes Version of the Common Terminology Criteria for Adverse Events.

^d^EORTC QLQ-C30: European Organization for Research and Treatment of Cancer Quality of Life Questionnaire-C30.

^e^ESAS: Edmonton Symptom Assessment Scale.

^f^PROMIS: Patient-Reported Outcomes Measurement Information System.

^g^BREAST-Q: Breast Questionnaire.

^h^ASyMS: Advanced Symptom Management System.

Table 1 shows a variety of countries involved in the research and development of ePROM interventions, mostly comprising European Union members, the United Kingdom, the United States, and Australia. The types of cancer represented most within the identified interventions are breast and colorectal. Except for the 7 studies that developed novel ePROM frameworks, most interventions incorporated established published tools, such as Patient-Reported Outcomes Version of the Common Terminology Criteria for Adverse Events (PRO-CTCAE), European Organization for Research and Treatment of Cancer (EORTC) Quality of Life Questionnaire (QLQ)-C30, and the Distress Thermometer; 1 study did not report this information [40-42]. Most interventions were designed to monitor an extensive list of symptoms, most commonly including fatigue, pain, and nausea. The symptom lists included in the ePROM intervention were not identified in 7 studies.

#### Summary of ePROM Collection Instruments and Frameworks

We grouped the validated ePROM instruments embedded in the included studies by aim and functionality into the following 7 categories.

First, many of the instruments were symptom screening tools that measured (1) presence and severity, and (2) the distress or interference caused by each symptom. This category included the Advanced Symptom Management System (ASyMS), the Brief Pain Inventory (BPI), the Chemotherapy Symptom Assessment Scale (C-SAS), the Chemotherapy Toxicity Self-Assessment Questionnaire (CTAQ), the Memorial Symptom Assessment Scale (MSAS), the PRO-CTCAE, and the Patient-Reported Outcomes Measurement Information System (PROMIS) [40,44,47-51].

The second category differed subtly from the first by comprising symptom screening tools that measure only symptom presence and/or severity, but not the distress level or the impact of a symptom on daily life or experience. This category included the Checklist Individual Strength, the EORTC QLQs, the Edmonton Symptom Assessment Scale (ESAS), Hospital Anxiety and Depression Scale (HADS), the Personal Health Questionnaire (PHQ) Depression Scales (PHQ-8 and PHQ-9), STAR, and the World Health Organization Well-Being Scale-5 (WHO-5) [41,52-59].

Four categories consisted of measures that focus on broader health-related aspects than symptoms. First, some tools incorporated generic measures of overall health, HRQoL, or distress level and/or problem checklists. These included the Distress Thermometer and Short Form Health Survey-36 (SF-36) [42,60]. Second, some of the questionnaires measured satisfaction with treatment and support, unmet needs, fear of cancer recurrence, and/or other patient-reported experience measures. These measures included HRQoL and overlapped with other outcomes. This category included the Breast Questionnaire, the Cancer Worry Scale-6, the Holistic Needs Assessment (Health MOT; an “MOT” refers to a British vehicle safety test named after the now-defunct Ministry of Transport), and the Supportive Care Needs Survey-ST9 [45,61-63]. Third, some questionnaires measured patient-reported behaviors using nutrition questionnaires, smoking and alcohol abstinence scales, and the Short Questionnaire to Assess Health-Enhancing Physical Activity [64-66]. Finally, some questionnaires measured patients’ knowledge, ability, and understanding, such as the Digital Health Literacy Instrument and the European Health Literacy Survey [67,68].

It is important to note that the listed tools were not entirely separate from each other, as some of the instruments were based on a mix of previously developed frameworks; for example, ASyMS integrated the Common Terminology Criteria for Adverse Events grading scale with the C-SAS instrument [48].

#### Summaries of Included Studies

Table 2 presents an abridged version of the SLR study summary table, the corresponding detailed version of which is presented in Table S1 in Multimedia Appendix 1. The full supplementary table contains detailed summaries of the included studies (n=27), listed alphabetically either by the study or project name or by the authors’ names if the study name was not available. As such, for the studies with multiple publications included in this review, data were extracted collectively by cross-referencing the grouped publications, rather than extracting each publication separately. This helped the extraction of complementary information to gain insight into the link between detailed ePROM characteristics and health economic outcomes. For example, the Sentinel HEA report did not include the detailed information on the ePROM intervention design and procedures reported in the main RCT paper [34,35].

**Table 2:** Abridged version of the study summary table.

Author (year; n=34)	Study/intervention (n=27)	Country	Type of publication
Aapro et al (2020) [60]	N/A^a^: review	International	Scoping review
Ferre et al (2021) [69]	N/A: no name found	Italy	Protocol: observational/NRCT^b^
Qaderi et al (2021) [70]	Affordable Better	Netherlands	Report: implementation
Schougaard et al (2016) [71]	AmbuFlex	Denmark	Report: implementation
Sprave et al (2020) [72]	APCOT	Germany	Protocol: RCT^c^
Smits et al (2025) [65]	CAG	Netherlands	Protocol: within-RCT HEA^d^
Mir et al (2022) [36]	CAPRI	France	Report: RCT
Minvielle et al (2024) [37]	CAPRI	France	Report: within-RCT HEA
Storm et al (2024) [73]	eHealth@ Hospital-2-Home	Norway	Protocol: within-RCT HEA
Absolom et al (2017) [31]	eRAPID	United Kingdom	Protocol: within-RCT HEA
Holch et al (2018) [32]	eRAPID	United Kingdom	Protocol: within-RCT HEA
Velikova et al (2022) [33]	eRAPID	United Kingdom	Report: within-RCT HEA
Absolom et al (2021) [4]	eRAPID	United Kingdom	Report: RCT
Dawkins et al (2024) [6]	eRAPID	United Kingdom	Report: within-RCT HEA
Maguire et al (2018) [50]	eSMART	Austria, Greece, Ireland, Norway, United Kingdom	Protocol: within-RCT HEA
Nanton et al (2017) [74]	ICARE-P	United Kingdom	Report: observational/NRCT
Schmalz et al (2020) [75]	Kaiku Health	Germany, Switzerland, Finland	Report: implementation
Kearns et al (2022) [76]	LYSA	Ireland	Protocol: within-RCT HEA
Koumakis et al (2021) [77]	MyPal	Greece, Italy, the Czech Republic, Sweden	Report: implementation
van der Hout et al (2020) [78]	Oncokompas	Netherlands	Report: within-RCT HEA
Crafoord et al (2025) [79]	PHONEME/Interaktor	Sweden	Report: within-RCT HEA
Breen et al (2015) [80]	PRISMS	Australia	Protocol: within-RCT HEA
Webb et al (2024) [81]	PROMISE	Australia	Protocol: within-RCT HEA
Girgis et al (2020) [5]	PROMPT-Care	Australia	Report: observational/NRCT
Xia et al (2025) [82]	PRO-NET	China	Protocol: within-RCT HEA
Denis et al (2017) [35]	Sentinel	France	Report: RCT
Lizée et al (2019) [34]	Sentinel	France	Report: within-RCT HEA
Wheelock et al (2014) [83]	SIS.NET	United States	Report: RCT
Basch et al (2016) [58]	STAR^e^	Canada and United States	Report: RCT
Nixon et al (2018) [39]	STAR	Canada and United States	Report: HEA model
Billingy et al (2021) [84]	SYMPRO-Lung	Netherlands	Protocol: within-RCT HEA
Frankland et al (2019) [85]	The Programme	United Kingdom	Report: observational/NRCT
van Egdom et al (2019) [86]	VBHC-initiative	Netherlands	Report: implementation
Dibble et al (2025) [87]	YES	United States	Protocol: RCT

^a^N/A: not available.

^b^NRCT: nonrandomized controlled trial.

^c^RCT: randomized controlled trial.

^d^HEA: health economic evaluation.

^e^STAR: Symptom Tracking and Reporting.

### Results of Individual Studies

#### Intervention Design

This review identified several distinct design frameworks for ePROM-integrated interventions from studies with health economic components. However, there was a considerable variation in the level of technical detail with which the evaluated digital system was described. In most cases, the intervention was designed according to the following pattern: (1) the patient remotely reported symptoms and completed ePROMs; then (2) the ePROM information triggered alerts or was processed into an action recommendation; after which (3) the ePROM information, the alert, and/or the action recommendation were sent to a designated agent responsible for deciding on further actions. The systems can be categorized by the actor responsible for making decisions following ePROM processing (“active” vs “reactive”), the extent to which decisions are automated, the type of service offered to patients following a decision, and any additional functionality fostering patient-HCP communication (eg, within-app messaging or consultation scheduling). In addition to the active and reactive approaches, some interventions do not use alerts and instead treat ePROM information as supplementary to support HCPs in making treatment decisions or structuring consultations.

In the *active* approach to decision-making and alert handling, ePROMs and/or ePROM-triggered alerts are sent to HCPs for actioning, whereas the *reactive* approach may entail sending ePROM-tailored self-help information and/or guidance on recommended actions to patients [82,84]. Examples of strictly or predominantly active-approach interventions are AmbuFlex, eHealth@Hospital-2-Home, ICECARE-P, MyPal, PROMISE, PROMPT-Care, Sentinel, and STAR [5,35,38,71,73,74,77,81]. Reactive-approach interventions include Affordable Better, CAG, CAPRI, PRO-NET, and Oncokompas [36,65,70,78,82]. Some interventions blend both methods into a hybrid approach, usually to distinguish between alert severity. In these cases, low-priority alerts are handled reactively by sending patient self-help information tailored to their symptom reports. In contrast, high-priority alerts are actively sent to HCPs with urgent action recommendations. Such “hybrid” approaches are present in the eRAPID, eSMART, LYSA, PHONEME/Interaktor, PRISMS, SIS.NET, and YES interventions [33,50,76,79,80,83,87]. Interestingly, the SYMPRO-Lung protocol outlines a plan to explicitly compare the active and reactive approaches in separate trial subgroups. For interventions that provide self-help information and guidance, it is important to distinguish those that are predominantly active, but allow patients to access information should they choose to do so independently of ePROM results (eg, eHealth@Hospital-2-Home), from those that are “proactively” reactive and send patients information linked to or triggered by their ePROMs (eg, CAG and PRO-NET) [65,73,82]. The intervention presented by Ferre et al [69], as well as VBHC, did not use alert handling and instead treated ePROM data as supplementary information for HCPs [69,86]. In the publications from Kaiku Health and the Programme, it is not explicitly clear how/if alerts are handled [75,85].

Examples of additional functionality embedded in the ePROM interventions, which contribute to patient-HCP communication at the cost of increasing intervention complexity, are in-app/portal appointment scheduling (eg, APCOT), in-app/portal patient-HCP videocall and messaging (eg, Affordable Better), and automated ePROM data summary dashboards displayed to HCPs and/or patients (eg, CAPRI, Ferre et al [69]) [36,70,72].

#### Cost-Effectiveness, Budget Impact, and HRU

**Figure 3:**
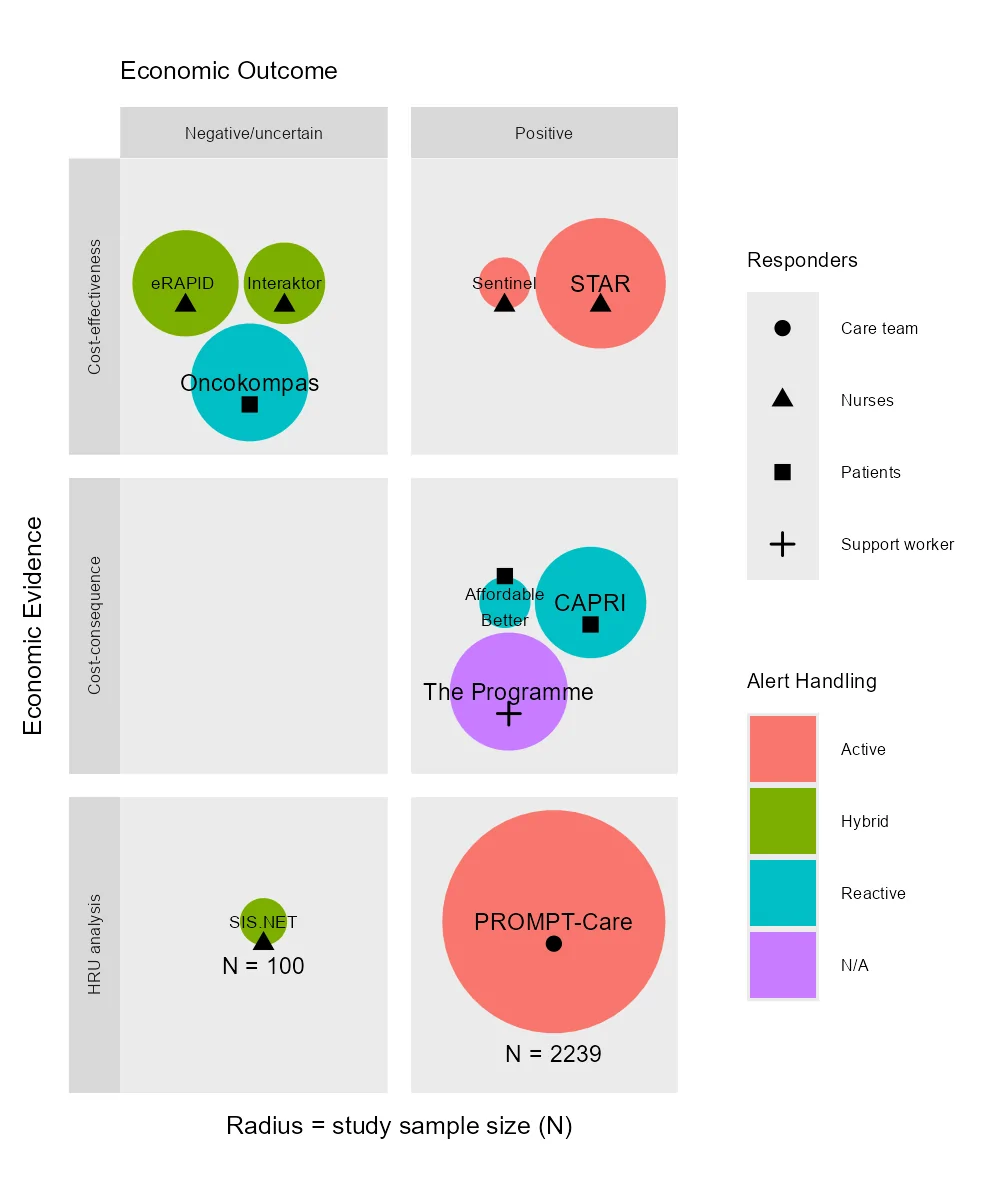
Map of economic evidence by economic outcome, analysis type, study sample size, and alert handling protocols. CAPRI: Impact of a Monitoring Device for Patients With Cancer Treated Using Oral Therapeutics trial; HRU: health resource use; STAR: Symptom Tracking and Reporting.

We identified 10 studies reporting economic results, grouped into full (cost-effectiveness), partial (cost-consequence), or HRU-only analyses. We visualized each study by (1) outcome, where “Positive” indicates ePROM-monitoring is associated with low incremental cost-effectiveness ratios (ICERs), cost savings, higher certainty, etc; (2) alert handling protocol, where “Responders” refers to the designated ePROM-informed decision maker; and (3) study sample size, shown in Figure 3.

Only 5 studies reported cost-effectiveness results, including ICERs (5/5 studies) and/or cost-effectiveness acceptability curves (3/5 studies), as shown in Table 3. From these, 2 studies (Sentinel and STAR) reported strong evidence for high ePROM cost-effectiveness relative to usual care, while 3 studies (eRAPID, PHONEME/Interaktor, Oncokompas) reported positive but more uncertain results [6,34,39,78,79].

**Table 3:** Extract of base-case health economic results.

Intervention (design, cancer)		Time horizon	Currency, year	Δ QALYs^a^/person (95% CI)	Δ Costs/person (95% CI)		Deterministic ICER^b^/result		WTP^c^: CE%^d^	DR^e^ (%)
Cost-effectiveness										
	eRAPID [6] (hybrid, multiple)	18 weeks	GBP, 2018	0.003 (–0.005 to 0.011)	–£25 (–£1241 to £1167)		eRAPID dominates		£20,000: 55%; £30,000: 58%	0
	STAR [39] (active, solid)	Lifetime (10 years)	CAD, —^f^	0.25	$3363		$13,450		$50,000: 90.8%	1.5
	Sentinel [34] (active, lung)	Trial (20 months)	EUR, —	Per-person: — (cohort-level: 4.59^g^)	€1963 (€1446 to €2480)		€20,912		€30,000: 97%; €90,000: 100%	0
	Interaktor [79] (hybrid, multiple)	6 months	EUR, 2022	0.0076	€1454		€13,213 to €202,368		—	0
	Oncokompas [78] (reactive, multiple)	6 months	EUR, 2017	0.0017 (–0.0121 to 0.0155)	–€163 (–€665 to €326)		Oncokompas dominates		—	—
Cost-consequence and budget impact										
	Affordable Better [70] (reactive, colorectal)	1 year	EUR, —	N/A^h^	Per-person: –€190 (Calculated from cohort-level: –€22,000^g^)		Cost-saving		N/A	N/A
	CAPRI [37] (reactive, multiple)	Trial (4.58 months)	EUR, 2022	N/A	–€377		Cost-saving		N/A	N/A
	The Programme [85] (N/A, prostate)	8 months	GBP, 2015	N/A	–£37		Cost-saving		N/A	N/A
Health resource use analyses										
	PROMPT-Care [5] (active, multiple)	Trial (30 months)	N/A	N/A	N/A		Emergency department visits reduced by 33% (*P*=.02), but no significant difference in outpatient HRU^i^	N/A		N/A
	SIS.NET [83] (hybrid, breast)	Trial (18 months)	N/A	N/A	N/A	No significant differences in rates of HRU (outpatient and test/scans)		N/A		N/A

^a^QALY: quality-adjusted life year.

^b^ICER: incremental cost-effectiveness ratio.

^c^WTP: willingness-to-pay (threshold).

^d^Probability of cost-effectiveness

^e^DR: discount rate.

^f^Not available.

^g^Summed values for entire cohort instead of per person estimates.

^h^N/A: not applicable.

^i^HRU: health resource use.

A total of 5 other studies (CAPRI, Affordable Better, the Programme, PROMPT-Care, and SIS.NET) reported reductions in costs or resource use within partial economic analyses alongside positive clinical or implementation results (Table 3). From these, 4/5 studies could have potentially yielded ePROM strategy dominance and/or high probabilities of cost-effectiveness following a full HEA [5,37,70,85]. On the other hand, 1 study (SIS.NET) did not support the hypothesized reduction in HRU for ePROMs; it thus might have yielded a result with a high and uncertain ICER and/or dominant standard care strategy following a full HEA [83].

We provided detailed study-by-study descriptive summaries of cost-effectiveness results accompanying Table 3 in the Multimedia Appendix 1. Table 3 also presents the alert-handling approach (see the “Intervention Design” section) and cancer type in each study.

In summary, evidence of QALY gains or health utility improvements from ePROM interventions is provided in Sentinel, STAR, and PHONEME/Interaktor. STAR, CAPRI, and PROMPT-Care report evidence that remote monitoring reduces emergency visits, hospitalizations, and/or hospitalization duration. The Sentinel and CAPRI trials demonstrated that the increased independence of ePROM-tracked patients shifts their health-related travel patterns from expensive (eg, ambulances and taxis) to cheaper (eg, walking and driving) modes. Conversely, significant differences across other HRU categories (eg, outpatient appointments and tests/scans) were not detected in the studies included in this review.

#### Qualitative Research

Twelve studies included qualitative research components (Table S2 in Multimedia Appendix 1 for the detailed extraction). Of these, most (7/12) conducted their qualitative research alongside or at the end of a pilot study or trial, while 3/12 did so during the design/planning stage, and 2/12 included multiple qualitative components throughout various study phases. A total of 11/12 studies conducted interviews, of which 7 used a semistructured framework, 1 used a think-aloud approach, while 3 did not specify the interviewing style. Overall, 3/12 studies organized focus groups and workshops, while 2/12 collected qualitative survey or feedback form data. Only 6/12 studies explicitly specified the type of qualitative analysis, which comprised thematic (n=5) and content (n=1) analyses.

For each of the 12 studies with qualitative components, we extracted the aims, participant numbers, and types (eg, patients, HCPs, or both), conclusions (if applicable), and a list of themes analyzed (Table S2 in Multimedia Appendix 1). In most instances, authors explicitly listed the qualitative themes present in their research. Still, we supplemented this in our extraction based on our reading of the studies or when thematic descriptions were limited. Only 1 study (VBHC) did not discuss or report its thematic approach [86].

We counted and categorized instances of each theme type in the 12 studies. The most common theme was “user experience,” included in 6 studies. This was followed by “acceptability,” “impact on care,” “self-management,” “adherence/adoption,” “implementation,” each of which featured in 3 studies. Then, “responses to alerts” and “usability” each appeared twice, while 7 studies included “other” themes. The “other” category consisted mostly of design- or implementation-related themes; for example, discussions on design recommendations or intervention fidelity (ie, “Was the intervention implemented as intended?”).

We evaluated the relevance of the identified themes to this review based on (1) how directly related they are to cost-effectiveness–related questions about ePROM integration and (2) how useful the insights from their analyses are in formulating health economic modeling assumptions and/or sensitivity/scenario analyses. Following our extraction of quantitative data, we formulated the following example questions that we hoped our qualitative extraction would address: How does ePROM integration impact time use and workloads? How/why does the design of the intervention (eg, active vs reactive alert handling) impact resource use?

However, the most common themes present in the included qualitative analyses were of limited health economic relevance and were instead related to app/platform users (eg, “user experience,” “acceptability,” and “usability”). These, in turn, involve discussions with users (eg, patients or HCPs) of an ePROM app or platform about its usefulness in health care–related communication, personalization, feedback features, and visual/interface design. In most of the identified papers discussing this theme, the authors report acceptance and enthusiasm from users related to these ePROM attributes, but do not link these findings to any health economic insights.

Two studies (VBHC and PHONEME/Interaktor) used qualitative methods to guide the quantitative outcome and HRU variables and questionnaire design [79,86].

Only 2 studies conducted qualitative research specifically related to health economics. In eRAPID, discussing the impact of the intervention on consultation time yielded mixed results, with HCP opinions divided between ePROMs being “possibly time saving in some instances” and ePROMs “adding time to the consultation.” However, the authors did not focus on this aspect or provide further detail in the report [33]. The PHONEME/Interaktor study qualitative components comprised (1) HRU variable identification (to guide and design the cost-effectiveness analysis) and (2) interviewing nurses about the impact of alert handling on workloads [79]. The latter component is by far the most relevant type of qualitative research to the aims of this SLR. Unfortunately, it is part of a separate, as-yet-unpublished manuscript, and therefore could not have been extracted for this review.

#### Impact of Setting and Intervention Design on Health Economic Results

Within the 10 studies reporting quantitative health-economic results, a high degree of heterogeneity in setting and intervention design obscured links between these characteristics and health-economic measures. The 2 studies that presented the most promising and complete evidence for ePROMs’ cost-effectiveness (Sentinel and STAR, see Table 3) were both active interventions, albeit set in different contexts (France and United States/Canada, respectively). Nevertheless, reactive interventions such as Affordable Better in the Netherlands and CAPRI in France demonstrated cost savings. As we discussed above, the evidence for other interventions (eg, hybrid-approach eRAPID) ranges from high uncertainty to a demonstration of ePROMs’ ineffectiveness.

We hoped to identify qualitative analyses explaining how and why active and reactive ePROM alert-handling approaches affect health outcomes and resource use. However, none were included in this review’s extraction. Therefore, we currently have limited insight into the effect of ePROM design on cost-effectiveness, but we have identified a study planning to address this gap in the future (SYMPRO-Lung) [84].

### Results of RoB and Reporting Quality Assessments

We conducted a detailed RoB and quality analysis for each study included in this review using appropriate validated checklists, the choice of which was guided by the National Institute for Health and Care Excellence guidance [88]. The full RoB and reporting quality descriptions and appraisal tables are included in the Multimedia Appendix 1. An abridged version of the appraisal process is presented in Table S16 in Multimedia Appendix 1, listing each publication by the checklist used, tool type (RoB vs reporting quality), and score or risk grade achieved.

This review did not include papers with a primary focus on qualitative research; as such, qualitative components were included in papers assessed with RoB tools designed for quantitative studies. However, we concluded that the qualitative research identified in this review was of basic quality and of limited use in informing health economic study design or modeling assumptions.

There is a risk of reporting bias in our SLR; only some of the clinical trials followed publicly accessible published protocols (eRAPID, CAPRI, and STAR), but we did not identify health economic analysis plans for any of the HEAs (Tables S17 and S18 in Multimedia Appendix 1) [31,36,38]. However, with only 20 heterogeneous reports of concluded studies (n≤6 per category, see Figure 2), this risk is difficult to assess.

In summary, the included studies in this review presented evidence of mixed quality and were inherently highly susceptible to contamination and diffusion biases due to the complex nature of ePROM-integrated interventions. Specifically, trials where HCPs treated both ePROM-monitored and usual care patients may have changed clinical practice for all participants due to being sensitized to previously undetected symptoms, thereby attenuating evidence supporting remote monitoring strategies [4,33].

### Model Parameter Extraction

#### Event Rates and Rate Ratios

Key parameters of interest for extraction in this review are HRU rates among patients using ePROM interventions, as well as corresponding rates and incidence rate ratios (IRRs) for comparison with standard (in-person) care. Ideally, these would be extracted from appropriately adjusted regression models from RCTs, but most studies did not report their results in this way. Papers predominantly reported raw, unadjusted counts of observed events per study group during follow-up, making comparisons across studies difficult.

However, using separately reported study follow-up information (eg, from the CONSORT [Consolidated Standards of Reporting Trials] diagrams), we calculated person-time and converted raw HRU counts into monthly rates for cross-compatibility and easy use in future economic modeling through the actuarial life-table approach given by 
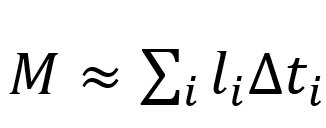
, where: [89].

- *M* denotes total person-time in months.
- *i* indexes the study time points/intervals.
- Δ*t* denotes the change in time (converted to months) between time points (interval length).
- l*i* denotes the average number at risk during the interval *i*, derived from:
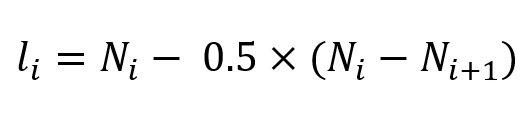
 using N*i* as the number of active patients in the study (ie, not deceased or withdrawn) at the *i*th time point.

For example, the CONSORT diagram presented in Absolom et al [4] shows the eRAPID arm having 256, 239, 222, and 214 patients on study at baseline, 6 weeks, 12 weeks, and 18 weeks, respectively, yielding 4176 person-weeks (≈960 person-months) when inserted into the corresponding equation. An excerpt of HRU and ePROM alert counts converted into monthly rates (events/person-month) is presented in Table 4; the full extraction is available in Table S3 in Multimedia Appendix 1.

**Table 4:** Abridged extractions of rates (events per person-month) and incidence rate ratios (IRRs).

Event				ePROM^a^		Control		ePROM/control, IRR (SE; 95% CI)
Affordable Better [70]: Colorectal cancer, posttreatment follow-up care (postsurgical)								
	Tests/scans			0.25		—^b^		—
	Parking (90 minutes)			4×10^–3^		—		—
	Transport			4×10^–3^		—		—
CAPRI [37]: Multiple cancers, commencing treatment (oral chemotherapy and/or molecular-targeted therapy)								
	Outpatient visits			4.08		4.13		0.99 (0.02; 0.95-1.03)
	Emergency visits			0.10		0.12		0.83 (0.12; 0.66-1.05)
	Hospitalizations			0.10		0.13		0.77 (0.12; 0.61-0.97)
	Trips (1-way)			1.07		1.27		0.84 (0.04; 0.78-0.91)
		Ambulance	0.12		0.14		0.86 (0.11; 0.69-1.06)	
		Light sanitary vehicle	0.26		0.35		0.74 (0.07; 0.64-0.86)	
		Taxi	0.69		0.78		0.88 (0.05; 0.81-0.97)	
	Death (adjusted HR^c^)			—		—		1.16 (0.17; 0.83-1.61)
eRAPID [4,90]: Multiple cancers, commencing treatment								
	ePROM-triggered alerts			3.45		—		—
		No problems (no alert)	0.11		—		—	
		Moderate (self-management)	2.83		—		—	
		Severe (prompt to contact HCPs^d^)	0.48		—		—	
		Emergency (alerts HCPs)	0.03		—		—	
PROMPT-Care [5]: Multiple cancers, active treatment or posttreatment follow-up care								
	Emergency visits			0.05		0.06		0.87 (0.06; 0.77-0.98)
	ePROM-triggered alerts			0.14		—		—
Sentinel [34,35]: Lung cancer, active treatment or posttreatment follow-up care								
	Consultations			0.31		0.22		1.38 (0.11; 1.11-1.72)
	Imaging tests			0.26		0.27		0.96 (0.11; 0.77-1.19)
	Trips (round)			0.41		0.29		1.41 (0.10; 1.17-1.72)
		Ambulance	3×10^–3^		0.02		0.17 (0.77; 0.04-0.78)	
		LSV^e^/taxi	0.19		0.16		1.21 (0.14; 0.93-1.59)	
		Private vehicle	0.19		0.11		1.73 (0.15; 1.28-2.34)	
		Public transport	0.03		0		—	
		On foot	0		3×10^–3^		—	
	Death (adjusted HR)			—		—		0.32 ± 0.38 (0.15, 0.67)
SIS.NET [83]: Breast cancer, posttreatment follow-up care (fully recovered from all acute serious side effects)								
	Physician visits			0.60		0.53		1.13
	Tests/scans			0.30		0.28		1.08
		Breast cancer-related	0.21		0.21		0.99	
		Non–breast cancer	0.11		0.07		1.56	
	Symptom reports			0.41		0.18		2.30
STAR^f^ [38]: Solid cancers, active treatment, mean (95% CI)								
	Death (adjusted rate)			0.024 (0.020-0.029)		0.031 (0.026-0.038)		N/A^g^
	Computer inexperienced			0.043 (0.028-0.062)		0.043 (0.028-0.062)		N/A
	Computer experienced			0.028 (0.022-0.036)		0.028 (0.022-0.036)		N/A

^a^ePROM: electronic patient-reported outcome measures.

^b^Not available.

^c^HR: hazard ratio.

^d^HCP: health care professional.

^e^LSV: light sanitary vehicle (non-ambulance medical transport).

^f^STAR: Symptom Tracking and Reporting.

^g^N/A: not applicable.

We calculated IRRs in Table 4 from available person-time and event rates or frequencies reported in the source paper. IRRs can be used to approximate hazard ratios if (1) the average follow-up is comparable between groups and (2) the relative hazards are constant [91]. A limitation of this approach in this context stems from a difficulty in verifying these assumptions (especially the latter) and the inability to account for competing risks using the cause-specific Cox proportional hazards model on individual-level data (as would be the case in a preplanned analysis) [92]. For example, the Sentinel trial followed patients in both arms for 587 days (from June 1, 2014, to January 9, 2016), broadly satisfying the first assumption despite an observed survival improvement in the ePROM arm resulting in slightly more person-months experienced than in the control arm (631.4 vs 601.4 person-months) [35]. However, HRU events reported by the study were discussed through the prism of differences between arms rather than over time, so this information is missing; it is reasonable to suspect that the amount of HRU events experienced by patients with lung cancer after or during treatment may change over time, thus violating the constant hazards assumption [34]. The method nevertheless yields approximations of useful modeling and sensitivity analysis parameters without access to sensitive patient-level trial data. We calculated SEs and CIs for IRRs using the following formulae (with *E* as the number of events observed in the ePROM=1 and control=0 groups) [93].

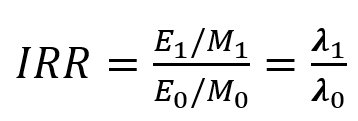
, where λ denotes the monthly rate.

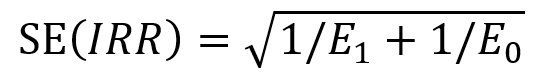

- 95% CI:
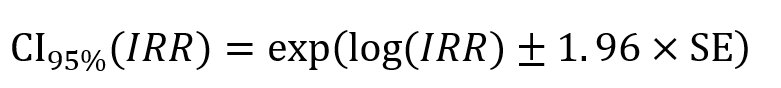

The IRRs derived from the HRU monthly event rates reported in the CAPRI trial are subject to additional uncertainty, as the authors did not report total event rates and were unable to provide them upon request [37]. However, the paper does state that the mean follow-up time used to calculate these rates was 4.58 months [37]. For crude IRR SE and CI estimation, we approximated the event count (*E*) values by multiplying the trial arm populations by the rate and the mean follow-up: N × λ × 4.56, a method endorsed by the authors (after contacting them). The estimated IRR CI widths broadly align with the reported *P* values for rate differences in the paper and support the authors’ conclusion of a significant reduction in hospitalizations with ePROM remote monitoring. However, IRRs are more useful than additive rate differences for economic model parameterization and sensitivity analysis.

The event rates IRRs from the SIS.NET study were not calculated on a per-person-time basis, as this was not reported in the paper’s CONSORT diagram [83]. Instead, the authors present mean per-patient 18-month values. The approximated monthly figures in Table 4 were derived by dividing the reported values by 18, so IRR SEs and confidence bounds could not be calculated.

The standard care arm and corresponding IRRs from the Affordable Better extraction are absent because the control arm was not empirical; the authors created a hypothetical counterfactual control based on the ePROM arm and assumptions about differences between hypothetical study arms in in-clinic and remote appointments [70].

The STAR monthly death rates were calculated from the reported adjusted 1-year survival results (*p*) using the probability-to-rate conversion formula, 
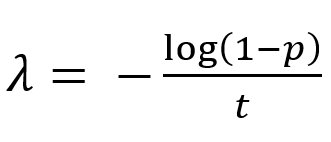
 where *t*=12 months (notes: constant hazard assumed, inverse CI bounds were swapped) [22,38]. The study presented statistically significant survival gains in the ePROM arm relative to control; the effect was greater in the computer-inexperienced subgroup [38].

#### Health Utility

Populating health modeling parameters requires health state utility values, rather than cumulative QALYs. EQ-5D–derived health utilities by trial arm and time point are reported in the eRAPID and STAR papers [33,38]. However, the eRAPID report only presented descriptive statistics (means and SDs) for EQ-5D scores, while the STAR paper reported CIs and adjusted between-arm differences and *P* values; STAR utilities were converted from their original 0-100 scale to a 0-1 scale in this extraction [33,38]. We presented extracted EQ-5D health utility data in Table 5.

**Table 5:** EQ-5D health utility scores.

Study and time point			ePROM^a^				Control				Between-group difference (*P* value)	
			EQ-5D score		Change from baseline		EQ-5D score		Change from baseline			
eRAPID [33] health utility (mapped to EQ-5D-3L), mean (SD; n)												N/A^b^
	Baseline		0.758 (0.185; 250)		N/A		0.753 (0.18; 248)		N/A			
	6 weeks		0.776 (0.175; 213)		–0.001 (0.183; 209)		0.752 (0.197); 226)		0 (0.176; 224)			
	12 weeks		0.747 (0.192; 202)		–0.028 (0.191; 196)		0.734 (0.18; 210)		–0.025 (0.178; 208)			
	18 weeks		0.739 (0.216; 189)		–0.052 (0.209; 184)		0.708 (0.213; 202)		–0.05 (0.212; 200)			
STAR [38] health utility (rescaled from 0-100 to 0-1), mean (95% CI)												
	Baseline (all), mean (95% CI)		0.862 (0.847 to 0.877)		N/A		0.866 (0.847 to 0.885)		N/A		N/A	
	6 months, mean (95% CI)		0.848 (0.832 to 0.864)		0.014 (–0.004 to 0.031)		0.795 (0.767 to 0.822)		0.071 (0.048 to 0.095)		N/A	
	Point drop difference (ePROM—control), mean (95% CI)		N/A		N/A		N/A		N/A		0.057 (<.001)	
	Computer inexperienced		N/A		N/A		N/A		N/A		0.065 (.01)	
	Computer experienced	N/A		N/A		N/A		N/A		0.057 (<.001)		

^a^ePROM: electronic patient-reported outcome measure.

^b^N/A: not applicable.

In the ePROM results, similar health utility patterns between arms are consistent with a lack of statistically significant QALY differences detected by the multivariate regression analysis. Conversely, STAR’s point drop differences can be interpreted as health utility gains due to effective ePROM monitoring; the increased (albeit less statistically powerful) difference in the computer-inexperienced patients is consistent with the subgroup’s overall more favorable results.

#### Alert Handling Statistics

Potentially important parameters for economic modeling of ePROM interventions include decision-tree or transition probabilities related to alert handling; for example, what is the probability that a patient is referred to the hospital after a medium/moderate alert? These are separate (but related) to the frequency rates of alerts presented in Table 4. Such probabilities can be derived from the results of the AmbuFlex and STAR reports.

AmbuFlex elicited 374 responses from patients with prostate cancer between September 2014 and December 2015, of which 38 (11%) were classed as “green” by the algorithm (ie, no further contact required), 128 (37%) were “yellow” (triage), and 181 (52%) were “red” (clinic referral); from the presented results, it is possible to infer that 52 (41%) of the “yellow” reports did not require further action, while 76 (59%) led to clinic referral [71].

The total number of individual symptom reports (n=84,212, of which 1431 were graded ≥3) is presented in the STAR intervention, but not the total number of email alerts to nurses (when symptoms worsened by ≥2 points or reached an absolute grade ≥3). However, simple percentages of nurses’ responses were reported: telephone counseling (77%), supportive medication initiation/change (12%), hospital/emergency referral (8%), chemotherapy dose modification (2%), and imaging/test orders (2%) [38]. Exact absolute values corresponding to these percentages are missing from the report and cannot be derived due to rounding; furthermore, these percentage sums slightly exceed 100% (also due to rounding) and would need to be adjusted for use in model parameterization.

In PROMPT-Care, authors present detailed breakdowns of the 877 alerts, with 44% (383/877) reviewed by HCPs, resulting in 496 actions. The action outcomes included no further follow-up (83/496, 17%), no response (111/496, 22%), or an in-clinic or remote follow-up appointment (302/496, 61%); follow-up actions comprised HCP-patient discussions (129/302, 43%), information provision (98/302, 32%), or onward referral (75/302, 25%) [5].

#### ePROM System Implementation and Maintenance

We identified 2 main cost categories of relevance to ePROM monitoring: (1) upfront setup, including IT, equipment, and web/app development, and (2) maintenance costs, comprising HCP wages, IT administration, etc. However, cost reporting was inconsistent across studies. We identified upfront cost estimates ranging from GBP 63-GBP 102/patient (GBP 1=US $1.3635 as of August 24, 2028) to GBP 141K (total), and running costs ranging from GBP 37/patient-year to GBP 217/patient-month (reported in 2024 GBP) [34,39,85,86].

We have included summaries of ePROM monitoring costs and details of currency conversion and inflation adjustments in the Multimedia Appendix 1.

#### Links Between Symptom Reports and HRU

For parametrizing a health economic model that incorporates ePROM-elicited information (eg, symptom severity, see Figure 1), information about the overall difference in rates of HRU between treatment strategies (as most concluded studies report) may be insufficient; it is also necessary to know the relationship between ePROM data and HRU (eg, given that a patient self-reported a high pain score, can they expect to be hospitalized sooner or more frequently?). However, this link is scarcely reported in the included studies.

The SIS.NET study reported the most direct link between ePROMs and HRU in the form of a direct positive correlation between symptom reporting rates and non–breast cancer–related appointments; however, only a *P* value (*P*=.0004) is provided, so no formula is available for use in a model [83].

**Figure 4:**
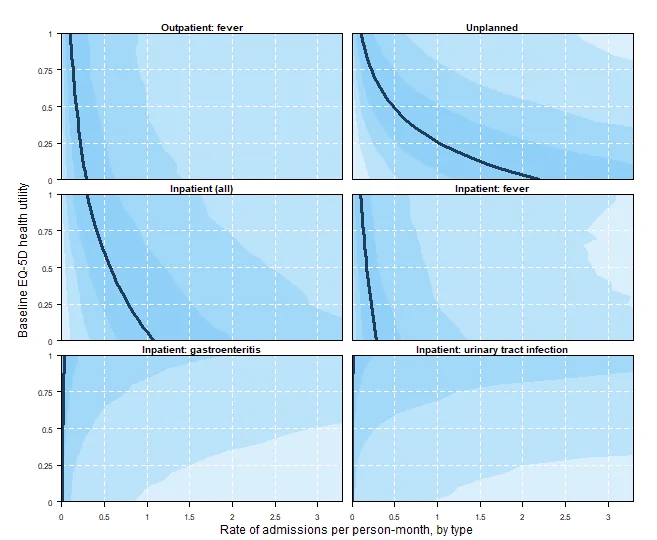
Probabilistic sensitivity analysis of monthly admission rate prediction formulae based on baseline EQ-5D.

A detailed, yet indirect, link between ePROMs and HRU rates is reported in the PHONEME/Interaktor study’s multivariate regression analyses [79]. The link is “indirect” because the patient-reported independent variable included is baseline EQ-5D health utility rather than disease-specific ePROM data (eg, symptom reports); with the right mapping algorithm and assumptions, a modeler may be able to use these formulae to predict HRU rates based on symptom scores. However, the regression tables only reported coefficient estimates without intercept values, rendering the formulae nonreconstructible; however, the authors provided the full results tables upon contact, which are provided in this paper’s Multimedia Appendix 1. Despite the absence of a covariance matrix, we performed a secondary analysis alongside this review based on the PHONEME/Interaktor formulae to demonstrate their use in a hypothetical model’s probabilistic sensitivity analysis. Example results of various HRU rate predictions for a patient with breast cancer aged 60 years after 2 neoadjuvant chemotherapy cycles with a Charlson Comorbidity Score of 1 are presented in Figure 4. The full formulae and the code used to perform this analysis are provided in the Multimedia Appendix 1.

## Discussion

### Summary of Key Findings

#### Literature Search

This review identified 27 unique complex digital interventions that embed patient-reported outcomes into clinical practice and decision-making. These elicit patient-reported information from either a variety of validated ePROM tools or novel frameworks. Most (but not all) systems include alert handling and/or automated decision support based on alert classification, using either an active or reactive approach to choosing an agent responsible for taking recommended actions.

We did not identify any form of guidance, methodology, or best practice specific to modeling oncological ePROM interventions.

#### Economic Evidence

There is mixed evidence on the cost-effectiveness of ePROM interventions, further complicated by the heterogeneity of intervention designs. However, out of the 5 studies that conducted cost-effectiveness analyses, 3 showed neutral or uncertain results, while 2 showed strong evidence in favor of integrating ePROMs into clinical practice.

Only 1 health economic model was identified in this review. However, it followed a simple 2-state Markov chain structure and is of limited use to potential future modelers aiming to adapt it or run more complex simulations or scenario analyses in different contexts. While such a model benefits from parsimony, its parameters are elicited from simple differences between ePROM and usual care trial arms and therefore could not be easily used in a simulation of a different intervention with an alternative design framework or alert handling mechanism.

#### Qualitative Evidence

Qualitative evidence that could support future economic modeling assumptions was searched within the included summaries. However, this was seldom present, with the qualitative focus centering more on user experience and intervention design-related themes. Only 1 study (eRAPID) explicitly reported limited economic-specific findings, indicating possible time savings for participating HCPs thanks to automated ePROM data summaries.

#### Parameter Extraction

The detailed parameter extraction we produced in this review, along with our recommendations for future research, could help modelers simulate the cost-effectiveness and test scenario analyses for a wide variety of ePROM interventions with heterogeneous design frameworks. For example, mapping relationships between self-reported symptoms and HRU patterns or generic HRQoL measures could enable the creation of early health economic models of upcoming ePROM systems that simulate the impact of different alert-handling and response mechanisms on long-term cost-effectiveness.

#### Cost-Effectiveness of ePROMs in Context

All of the economic evidence of ePROM monitoring we identified in this review comes from high-income countries where internet usage rates exceed 90% [94]. Most of these are European states with government-run or socialized health service providers.

With only 10 studies reporting heterogeneous economic results, it is difficult to infer the impact of other contextual aspects on PROM monitoring’s cost-effectiveness (eg, staffing or baseline service use). However, we did identify broadly favorable results for both active and reactive interventions with a variety of organizational setup structures (Figure 3), which designate the task of responding to ePROM-generated symptom reports to patients, nurses, or care teams, etc.

### Study Limitations

The main limitation of this review (especially for the data extraction) is the inclusion of the biases present in the included papers and some risk of reporting bias. Many of the RCTs from which the most robust cost-effectiveness evidence was extracted were especially prone to contamination and diffusion biases, which could potentially lead to an underestimation of the benefits of integrating ePROMs. However, these biases and reporting quality considerations were reported in detail and summarized so that future health economic modelers referencing this paper’s results can easily understand and acknowledge these limitations.

A secondary limitation is present in the secondary analyses of the included studies’ results, such as the calculation of ePROM vs standard care IRRs for HRU consumption, or the probabilistic sensitivity analysis of the PHONEME/Interaktor HRU prediction formulae. This is because, unlike the rest of the systematic review, these post hoc analyses were not prespecified in the methods section and therefore prone to selection bias or arbitrary method selection. However, the associated limitations are acknowledged and documented throughout this paper. Furthermore, it would have been difficult to predict the possibility or usefulness of these calculations before conducting this review and extracting the data.

Prospective health economic model developers should note the limitations of our crude, unadjusted IRR estimates derived from raw event counts. These estimates are useful only in the absence of methodologically superior adjusted estimates and/or Cox regression outputs, which our review did not identify; should more rigorous estimates be published, they should be preferred in model parametrization.

The quality and scope of our extractions and summaries may have been limited by our researchers’ (MA and AB) background in health economics. This was somewhat counteracted by our supervisors’ expertise in clinical oncology (PSH) and implementation science (KC), but future researchers in this area should form more interdisciplinary teams to support mixed methods syntheses.

Other limitations include restricted databases searched, positive publication bias, limited transferability across contexts, and an English-only search language; published evidence that could have affected our conclusions may have been available in other languages. Our inclusion of only 1 modeling paper further limits our ability to draw long-term conclusions about the cost-effectiveness of ePROMs.

### Integration of Findings With the Current Literature

This paper builds on prior reviews of PROM-monitoring interventions, offers a specific focus on the remote/digital aspect of complex intervention design and cost-effectiveness, and provides a much more detailed data extraction with secondary analyses. Our granular calculations of differences in HRU event rates bridge the gap between raw study results and usable estimates in a modeling input parameter table, while our analysis of PHONEME/Interaktor formulae provides an intermediate proxy of baseline EQ-5D for mapping ePROM self-reported symptoms onto different types of HRU events. The conclusions of our review are broadly consistent with those of Lizán et al [13], although areas of focus differ [13].

### Gaps in the Literature and Implications for Research and Practice

#### Effects of Integrating ePROMs on HRU

This review extracted and presented a heterogeneous set of HRU consumption rates, which could provide future modelers with the tools necessary to simulate differences in resource use and, therefore, costs between ePROM and standard care treatment strategies. Furthermore, this toolset was expanded by our secondary analysis of extracted results, in which we standardized all rates into comparable (monthly) units and produced IRRs. Event rates of patients with cancer by treatment strategy are well-evidenced and need not be a priority for future research relative to other gaps identified in this review.

The usefulness of the IRRs reported in Table 4 stems from enabling (1) the simulation of control populations from data collected in observational studies of ePROM interventions and (2) the construction of useful scenario analyses for economic models. For example, in a model scenario in which ePROM interventions increase patients’ independence in health-related travel (ie, more trips by public transport and fewer by ambulance), IRRs derived from the Sentinel study could be used. In contrast, CAPRI-derived IRRs could be used in scenarios where ePROM interventions lower hospitalization rates [34,37]. However, most of the IRRs in Table 4 were produced from this review’s secondary analysis of the included studies’ results and are therefore crude and unadjusted by any appropriate regression model.

We suggest that prospective modelers use our post hoc estimates with caution and care to match the context of interest. For example, IRR estimates from Sentinel may be most applicable to lung cancer populations in high-income settings with active alert-handling designs; extrapolating to other patient groups should be accompanied by transparent disclaimers of limitations and restricted to sensitivity/scenario rather than base case analyses.

To improve this evidence, future studies evaluating ePROMs and HRU consumption should include baseline-adjusted Cox proportional hazard or multivariate regression models using either ePROM study arm membership or granular self-reported symptom scores as predictors of HRU rates.

#### Health Utility and ePROM-Related Health States

The extraction of utility values revealed a limited availability of data needed to build a health economic model. Table 5 includes health utility values for ePROM and standard treatment strategies, not for different health states per se (Figure 1). To effectively parametrize a model that tracked or simulated patients’ ePROM data (eg, symptom severity), a mapping algorithm from disease-specific ePROMs into generic measures such as EQ-5D-5L–derived health utility would be needed. A targeted PubMed search revealed validated algorithms for some ePROM instruments used by interventions included in this review (eg, EORTC QLQ-C30 and ESAS), but a lack of evidence for others (eg, PROMIS and novel instruments).

While we are not aware of any other ongoing methodological work in this area, we echo the recommendations provided by an SLR of mapping algorithms in rare diseases (including some cancers) [95]. Its authors (Meregaglia et al [95]) encouraged the development of algorithms with broader generalizability, improved quality, and higher robustness; they also cautioned future researchers against reproducing common errors their review identified (eg, overestimating health utility for severe health states, especially when using ordinary least squares regressions).

Developing mapping algorithms for a broader set of measures and symptom scores for ePROM profiles should be a priority for quality-of-life research in cancer care. It would also help future modelers effectively assign health utility values to symptom-severity–stratified health states. More useful yet would be a calculation of health utility by overall alert severity grade, which was not identified in any of the studies included or elsewhere.

#### Implications for Further Qualitative Research

Overall, this review failed to identify the kinds of qualitative research it searched for (ie, qualitative insights that could inform economic modeling assumptions or explain *how* or *why* ePROMs change HRU patterns). As a result, modelers may not have sufficient information to properly structure a decision-analytic design. We summarized the gaps and proposed research needed to address them in this section.

The conclusions of the completed qualitative research (in 6/12 studies) reported broadly positive feedback, enthusiasm, and potential related to the implementation and design aspects of the ePROM interventions. Other than (to a very limited extent) eRAPID, none of the studies presented qualitative results that could constitute health economic model structure guidance or inform modeling assumptions. To address this, a mixed methods approach could be taken in a future study, such as including qualitative interviews within a time-motion study, as shown by Singh et al [96].

Furthermore, some areas in our quantitative extraction would benefit from qualitative insight. Chiefly, Lizée et al [34] found that ePROM-monitored patients used cheaper, more independent forms of travel (eg, more walking and fewer ambulance trips), but did not explain the mechanism behind this phenomenon.

To address the gaps we identified, we recommend focusing qualitative research on answering the following questions: (1) “How does ePROM monitoring impact time use and/or workload?,” (2) “How do different designs of ePROM monitoring frameworks and alert handling mechanisms impact HRU and/or HRQoL?,” and (3) “How/why does ePROM monitoring improve patients’ travel modes and independence or functioning?” Answering these questions through embedding time-motion studies within trials and/or using framework analysis focused on workflow and resource-use themes could help modelers validate model assumptions and more appropriately assign input parameters that match the intervention they wish to simulate.

#### Equity and Distributional Cost-Effectiveness

Equity concerns relevant to ePROMs include digital literacy, limited internet access, disability, language barriers, and limited caregiver support. However, our review has identified limited insights into these issues.

The main exception is the STAR trial, which demonstrated greater benefits from monitoring for patients without prior computer experience [38]. However, the intervention design was altered for those patients to an in-clinic-only schedule, rendering the ePROM collection nonremote for that subgroup.

None of the studies in our review conducted a distributional cost-effectiveness analysis (DCEA). We echo Meunier et al’s [97] recommendations for HTA agencies and governments to publish standardized DCEA methodological guidance and indices to enable future researchers to incorporate routine DCEA implementation in their studies. This would be especially useful in ePROM monitoring economic research.

### Conclusion

This review provides a comprehensive overview of the evidence on the cost-effectiveness of ePROM-integrated interventions in cancer care. There is some limited evidence that ePROMs *can* be cost-effective, but more research is needed to help with their economic evaluation. This review presents recommendations for both qualitative and quantitative research needed to improve the evidence base for these potentially transformative interventions. We recommend (1) quantitative research that can map specific ePROM data onto HRU patterns and generic HRQoL to inform future economic models and (2) qualitative research that provides insight into time use, workloads, the impact of ePROM design on HRU, and changes to patient-perspective resources (eg, health-related travel). We also provide detailed modeling parameter tables that serve as a handbook for researchers seeking to simulate the cost-effectiveness of complex digital interventions with remote symptom monitoring.

## Data Availability

All data and code used in this review’s analyses are present in the Multimedia Appendix 1.

## Appendix

Multimedia Appendix 1 Supplementary chapters, figures, tables, and code.

Multimedia Appendix 2 PRISMA checklist.

## Abbreviations

ASyMS: Advanced Symptom Management System
BPI: Brief Pain Inventory
CHEERS: Consolidated Health Economic Evaluation Reporting Standards
CONSORT: Consolidated Standards of Reporting Trials
C-SAS: Chemotherapy Symptom Assessment Scale
CTAQ: Chemotherapy Toxicity Self-Assessment Questionnaire
DCEA: distributional cost-effectiveness analysis
EORTC: European Organization for Research and Treatment of Cancer
ePROM: electronic patient-reported outcome measure
ESAS: Edmonton Symptom Assessment Scale
GRADE: Grading of Recommendations, Assessment, Development and Evaluation
HADS: Hospital Anxiety and Depression Scale
HCP: health care professional
HEA: health economic evaluation
HRQoL: health-related quality of life
HRU: health resource use
HTA: health technology assessment
ICER: incremental cost-effectiveness ratio
INAHTA: International Health Technology Assessment Database
IRR: incidence rate ratio
MSAS: Memorial Symptom Assessment Scale
PHQ: Personal Health Questionnaire
PRISMA: Preferred Reporting Items for Systematic Reviews and Meta-Analyses
PRISMA-P: Preferred Reporting Items for Systematic Review and Meta-Analysis Protocols
PRO-CTCAE: Patient-Reported Outcomes Version of the Common Terminology Criteria for Adverse Events
PROM: patient-reported outcome measure
PROMIS: Patient-Reported Outcomes Measurement Information System
QALY: quality-adjusted life year
QLQ: Quality of Life Questionnaire
RCT: randomized controlled trial
RoB: risk of bias
SF-36: Short Form Health Survey-36
SLR: systematic literature review
STAR: Symptom Tracking and Reporting
WHO-5: World Health Organization Well-Being Scale-5
