# Supplementary material for: Cost-Effectiveness of Electronic Patient-Reported Outcome Measure Interventions in Cancer: Systematic Review and Parameter Extraction for Economic Modeling
Source: J Med Internet Res. 2026 Sep 11;28:e100505. doi: 10.2196/100505 (PMC13615380; doi:10.2196/100505)
Supplement: Supplementary file 1 [file jmir_v28i1e100505_app1.docx]

# Appendix

Contents

[Appendix	1](#_Toc228962595)

[List of tables	2](#_Toc228962596)

[Database search strings	3](#_Toc228962597)

[Ovid (title, abstract, headings)	3](#_Toc228962598)

[Scopus	3](#_Toc228962599)

[International HTA database (title/abstract/keywords)	3](#_Toc228962600)

[Summaries of studies & ePROM interventions	4](#_Toc228962601)

[Summary of qualitative research components	16](#_Toc228962602)

[Parameter Extraction	18](#_Toc228962603)

[Phoneme/Interactor Multivariate Regression Details Sent by Authors	20](#_Toc228962604)

[R code used to produce secondary analysis of PHONEME/Interactor results	27](#_Toc228962605)

[Risk of Bias and Reporting Standards Checklists	33](#_Toc228962606)

[Appendix references	55](#_Toc228962607)

## List of tables

[Table 1: Full extraction table of included study summaries	5](#_Toc228962631)

[Table 2: Summaries of qualitative research components within included studies	16](#_Toc228962632)

[Table 3: Extractions of rates (events per person-month) and incidence rate ratios (IRRs)	18](#_Toc228962633)

[Table 4: Parameter Estimates Acute outpatient visits for fever	20](#_Toc228962634)

[Table 5: Parameter Estimates Unplanned admission to inpatient care	21](#_Toc228962635)

[Table 6: Parameter Estimates Acute inpatient visits all	22](#_Toc228962636)

[Table 7: Parameter Estimates Acute inpatient visits for fever	22](#_Toc228962637)

[Table 8: Parameter Estimates Acute inpatient visits for gastroenteritis	23](#_Toc228962638)

[Table 9: Parameter Estimates Acute inpatient visits for anaemia	23](#_Toc228962639)

[Table 10: Parameter Estimates acute inpatient visits for urinary tract infection	24](#_Toc228962640)

[Table 11: Parameter Estimates Outpatient care visits (all)	25](#_Toc228962641)

[Table 12: Parameter Estimates Patients with an acute outpatient visit yes/no	25](#_Toc228962642)

[Table 13: Parameter Estimates Patients with acute visit for urological problem	26](#_Toc228962643)

[Table 14: Variables in the Equation Patients with an unplanned admission to inpatient care	26](#_Toc228962644)

[Table 15: Parameter Estimates Patients with an inpatient visit yes/no	27](#_Toc228962645)

[Table 16: Risk of bias and reporting standards checklists	35](#_Toc228962646)

[Table 17: Consolidated Health Economic Evaluation Reporting Standards 2022 Checklist	36](#_Toc228962647)

[Table 18: Revised Cochrane risk-of-bias tool for randomised trials (RoB 2)	40](#_Toc228962648)

[Table 19: Risk Of Bias in Non-randomized Studies – of Interventions (ROBINS-I) assessment tool	44](#_Toc228962649)

[Table 20: Mixed Methods Appraisal Tool	51](#_Toc228962650)

[Table 21: Preferred Reporting Items for Systematic reviews and Meta-Analyses extension for Scoping Reviews (PRISMA-ScR) Checklist	52](#_Toc228962651)

## Database search strings

### Ovid (title, abstract, headings)

(("ePRO*" OR "e-PRO*") OR (("electronic" OR "web-based" OR "web ADJ based" OR "online" OR "digital" OR "remote") AND ("PRO" OR "PROs" OR "PROM" OR "PROMs" OR "patient-reported" OR "patient ADJ reported" OR "self-report*")))

AND

("cancer" OR "tumo*" OR "oncology" OR "neoplasm" OR "carcinoma")

AND

("econom*" OR "cost ADJ effect*" OR "cost-effect*" OR "cost ADJ utility" OR "cost-utility" OR "cost ADJ benefit" OR "cost-benefit" OR "cost ADJ consequence" OR "cost-consequence" OR "health ADJ resource" OR "HRU" OR "markov" OR "simulation" OR "decision ADJ analytic" OR "decision-analytic" OR "decision ADJ model")

### Scopus

TITLE-ABS-KEY ( ( ( "ePRO*" OR "e-PRO*" ) OR ( ( "electronic" OR "web-based" OR "web PRE/0 based" OR "online" OR "digital" OR "remote" ) AND ( "PRO" OR "PROs" OR "PROM" OR "PROMs" OR "patient-reported" OR "patient PRE/0 reported" OR "self-report*" ) ) ) AND ( "cancer" OR "tumo*" OR "oncology" OR "neoplasm" OR "carcinoma" ) AND ( "econom*" OR "cost PRE/0 effect*" OR "cost-effect*" OR "cost PRE/0 utility" OR "cost-utility" OR "cost PRE/0 benefit" OR "cost-benefit" OR "cost PRE/0 consequence" OR "cost-consequence" OR "health PRE/0 resource" OR "HRU" OR "markov" OR "simulation" OR "decision PRE/0 analytic" OR "decision-analytic" OR "decision PRE/0 model" ) )

### International HTA database (title/abstract/keywords)

(("ePRO*" OR "e-PRO*") OR (("electronic" OR "web-based" OR "web based" OR "online" OR "digital" OR "remote") AND ("PRO" OR "PROs" OR "PROM" OR "PROMs" OR "patient-reported" OR "patient reported" OR "self-report*")))

AND

("cancer" OR "tumo*" OR "oncology" OR "neoplasm" OR "carcinoma")

## Extraction: Summaries of studies & ePROM interventions

In Table 1, the “Study & Intervention” column provides a brief description of the ePROM intervention (or review aims), lists and cites the publication types included from the study, lists the countries involved along with the publication year(s), describes the anti-tumor treatment type and stage of the target cancer population, and provides the sample size(s) included in the study. The “ePROM Setup” column lists and cites the ePROM tools and frameworks included in the intervention design, describes (if available) the symptom scales used to elicit patient responses (e.g., traffic light system, Likert scales, etc.), and provides a description of the algorithm used to triage patients based on ePROM responses, manage alerts to HCPs, or issue self-help guidance or resources to patients. The following two columns present the key assumptions, processes, and findings or results related to (a) what actions the intervention in question expects from a given stakeholder, and (b) the intended or observed outcomes the intervention generates for that stakeholder. The stakeholders in question are the intended users, comprising patients whose symptoms are monitored, and HCPs tasked with responding to symptom scores and alerts. The “System/Organization” column presents information related to an ePROM intervention’s interaction with the health system in general and/or the organization within which it is implemented. The “HEA” column presents either a summary of findings specific to cost-effectiveness or a description of HEA components planned or conducted during the study. Where the information was available, the HEA column describes the economic evaluation design parameters; for example, the perspective is specified either as “healthcare [payer]”, usually implying the inclusion of costs directly incurred by a country’s National Health Service (NHS), or as a broader “societal” perspective, which also includes illness-related productivity costs and patient out-of-pocket costs.

Table 1: Full extraction table of included study summaries

| Study & Intervention | ePROM Setup | Patient | HCP | System/Organization | HEA |
| --- | --- | --- | --- | --- | --- |
| **Aapro et al. (2020)**  ***Review:*** To analyze the benefits & limitations of eHealth interventions in oncology supportive care  ***Publications:*** Scoping review^1^  ***Setting:*** International, 2020  ***Cancers:*** All  ***Sample:*** 66 full-text articles included | N/A | ***ePROM benefits:*** Greater patient-centricity, improved communication with HCPs, shared decision-making, better treatment adherence, continuous access to reliable clinical/treatment information, and reduced reliance on non-scientific web sources.  ***ePROM limitations:*** technology challenges, need for training, time-consuming, and depersonalization of care. | ***ePROM benefits:*** Better communication and shared decision-making, real-time data and toxicity management, visible patient improvements, effective time management, selective patient contact, improved supportive care, reduced HRU. ***ePROM limitations:*** Technology difficulties, need for training and workflow adaptation, extra time outside consultations (including patient/caregiver education), and resistance to altering established practices. | ***ePROM benefits:*** cost savings and reduced HRU from preventative care, more real-world data. ***ePROM limitations:*** need for training, lack of processes/ regulation, increased time and cost of evaluation. Implementation may be associated with HCPs learning new technologies, accepting and adapting to changes, and reorganizing multidisciplinary teams/treatment centers, and eHealth system management complexities. | The review finds mixed evidence on eHealth's effect on patients' HRU. 1 study shows no effect, while 4 studies show some benefits (reduction in patients' emergency & unscheduled visits, length of stay, outpatient visit frequency & duration, or travel-related costs). |
| **Affordable Better**  ***Intervention:*** Remote follow-up system  ***Publications:*** Cohort/ implementation^2^  ***Setting:*** The Netherlands, 2021  ***Cancers:*** Colorectal  ***Treatment:*** Biannual follow-up for ≤5 years with carcino- embryonic antigen monitoring, abdominal & chest scan schedule  ***Sample:*** N = 118 | ***Instruments*:** EORTC (QLQ-C30), CWS-6, HLS-EU, DHLI  ***Algorithm:*** No alerts or ePROM-based decision algorithms are described. | Patients use the intervention to access test results, self-help, telemedicine applications (video-consults, text messaging, and hospital telephone services). | HCPs sent test results remotely to patients through the digital system instead of discussing them at prescheduled visits. | Standard outpatient clinical visits were not prescheduled, leading to most patients not seeking contact after receiving test results. | The study compared the cost of the CRC follow-up for included patients against a hypothetical counterfactual (had the remote follow-up not been implemented) from a healthcare perspective and found €22,408 cost-savings for the patient group due to fewer in-person and remote clinical contacts. |
| **AmbuFlex**  ***Intervention:*** An ePROM-based monitoring and outpatient scheduling tool during follow-up in chronic and malignant disease  ***Publications:*** Implementation^3^  ***Setting:*** Denmark, 2016  ***Cancers:*** Prostate, colorectal (& multiple non-cancers)  ***Treatment:*** Various  ***Sample:*** 13,135 outpatients | ***Instruments*:** WHO-5, SF-36, HADS, EORTC QLQ-C30  ***Symptom scale:*** green, yellow, red  ***Algorithm:*** All green responses = no contact needed, ≥1 yellow response = clinician decides on action based on full ePROM report, ≥1 red response = patient must be seen/contacted. | ***ePROM completion:*** 3-monthly or at pre-specified time-points (3, 6, or 12 months), depending on disease  ***Design:*** Patients do not have scheduled outpatient visits and instead rely on AmbuFlex, which is therefore fully integrated and not optional.  ***Results:*** For colorectal cancer patients, 11% of responses were green and needed no follow-up (yellow = 37%, red = 52%), leading to 26% of responses requiring no further contact. | HCPs receive an alert list with tracked patients with red and yellow status and non-responders. HCPs access the graphical overview for clinical decision support. HCPs participated in the threshold design of the AmbuFlex decision support. | AmbuFlex includes ePROM data collection, an ePROM-based automated decision algorithm, and an ePROM-based graphical overview for clinical decision support. The authors state that up to half of the visits may be replaced by a less resource-demanding activity (depending on disease type) due to ePROM-based decision support.  For colorectal cancer, ePROMs were used to assess pre-chemo health to prescribe chemotherapy in advance. | No standard HEA or HRU reporting is included. However, the paper presents a distribution of ePROM alerts (green, yellow, red), further tabulated by percentages, leading to a decision outcome (no action, contact with clinic referral). This could be very useful in decision tree model parameterization. |
| **APCOT**  ***Intervention:*** App monitoring during chemo/radiotherapy  ***Publications:*** Trial protocol^4^  ***Setting:*** Germany, 2020  ***Cancers:*** Head and neck  ***Treatment:*** Current chemo/radiotherapy + weekly HCP appointments & blood tests  ***Sample:*** 50 per group target | ***Instruments*:** EORTC (H&N35)  ***Algorithm:*** No exact algorithm or alert system discussed beyond regular monitoring & in-app appointment scheduling. | ***ePROM completion:*** daily  ***Design:*** Provided with a mobile app on a tablet computer before each radiation treatment fraction. Patients may request HCP appointments in-app. | The radiation technologist is informed by an automatic notification system before applying the daily treatment and will schedule a consultation with the treating physician on that day. | ePROM patients are hypothesized to be scheduled for earlier medical interventions. ePROMs may allow patients to report information from outside the center (data completeness). Selection bias is discussed for users more technically adept. | No planned HEA, but the study will report frequency and total duration of consultations (ePROM vs control), which will be very useful for economic modelling parameterization. |
| **CAG**  ***Intervention:*** Blended intervention (ePROM-based self-help + consultations)  ***Publications:*** Trial protocol^5^  ***Setting:*** The Netherlands, 2025  ***Cancers:*** All  ***Treatment:*** Completed primary treatment of cancer (e.g., radio/chemo/surgery) between 6 weeks and 3 years prior and are in watchful waiting condition  ***Sample:*** 188 per group target | ***Instruments*:** SQUASH, HADS, CIS, EORTC (QLQ-C30), Smoking/alcohol abstinence scales, Nutrition questionnaires  ***Algorithm:*** The personalized Module Referral Advice (MRA) is created from baseline responses, referring patients to the highest-priority self-help modules to complete and discuss with HCPs. | ***ePROM completion:*** 6-monthly  ***Design:*** Participants receive a personalized Module Referral Advice (MRA) from baseline responses, access and complete relevant online modules at home, share their MRA with their HCP, discuss and select focus areas in the first consultation, work on these for 4–6 weeks, review progress in 2 follow-up consultations, and retain CAG access for up to 6 months | HCPs conduct shared decision-making, motivate participants to choose at least 1 module of focus for the next 4-6 weeks, discuss progress, and help with advice interpretation. | Authors discuss organizational aspects of recruitment for intervention through general practice centers (e.g., treatment initiated by patient request, usually complaint-driven), minimizing non-response, and detail organizational/set-up costs included in the HEA. | Not concluded (protocol); the study will conduct a within-trial HEA from the societal perspectives |
| **CAPRI**  ***Intervention:*** Nurse-led remote patient monitoring, symptom reporting, messaging, and self-help  ***Publications:*** RCT^6^, HEA^7^  ***Setting:*** France, 2024  ***Cancers:*** All  ***Treatment:*** Oral chemotherapy and/or molecular-targeted therapy + oncology consultations.  ***Sample:*** N = 559 (intervention = 272, control = 287) | ***Instruments*:** Novel (CAPRI)  ***Algorithm:*** Automatic alerts are based on ePROM thresholds. Manual alert options are provided via nurse navigators during follow-ups or by any HCP during a message/call appointment. Authors do not describe an exact algorithm, except that nurse navigators evaluate alerts and take actions based on NCI-CTCAE classifications and guidance. | Patients use CAPRI to report & track symptoms, message nurse navigators, and access self-help resources. Results show CAPRI patients have reduced hospitalizations and shorter length of stay, fewer monthly uses of light medical vehicle transportation, but more GP consultations | HCPs access dashboard linked to ePROMs (symptom AEs and alerts). Nurse navigators monitor alerts and report actions (give advice, make referrals, or arrange appointments/hospitalization). Nurse navigators can use the system to communicate with other HCPs. | Two Nurse Navigators monitor CAPRI 9am-5pm on workdays. The system tracks nurse navigator action reports. Authors report CAPRI program costs in two perspectives: healthcare payer = €103 /patient/month (€75/month for the digital solution + €28/month for the care structure, including the nurse navigator’s salary), societal = €87,23/patient/month based on annual subscription of €10K and nurse navigator salary - detailed calculations are unclear. | CAPRI estimates a monthly saving of €82.37 from a healthcare perspective, mainly due to reduced hospitalizations (and shorter length of stay). Savings compensated for monitoring costs. No increase in outpatient HRU due to ePROM monitoring was observed, except for GP visits. Detailed HRU and cost breakdowns (means and differences) are presented in the paper, which could offer many useful parameters for economic modelling. |
| **eHealth@Hospital-2-Home**  ***Intervention:*** Nurse-assisted remote monitoring of vital signs; self-reported symptoms, health and well-being  ***Publications:*** Trial protocol^8^  ***Setting:*** Norway, 2024  ***Cancers:*** Colorectal  ***Treatment:*** Curative surgery  ***Sample:*** 120 per group target (heart failure = 90, colorectal cancer = 30) | ***Instruments*:** Novel (Dignio)  ***Algorithm:*** Each participant profile will have personalized pre-defined thresholds (green, yellow, red). Yellow/red = 48h response; Red = request for extra clinical measures to confirm correctness, followed by advice to contact GP or emergency department (no automated emergency service). | ***ePROM completion:*** daily  Patients monitor vital signs via medical devices connected to an issued tablet (heart rate, blood pressure, etc.), self-report symptoms, access self-help or educational resources, and can message or videochat with HCPs if "vital signs exceed the acceptable range". | Nurse "navigator" operates a non-acute, alert, and communication-based service; they must respond to patients' questions and give feedback and advice. The CEA includes nurse navigators' weekly work hours spent connected to MyDignio patients or following up with patients through DignioPrevent. | ***Logic model:*** The intervention is hypothesized to increase health condition awareness, prompt health-seeking behavior, facilitate patient-HCP communication, and increase self-management/efficacy. | Not concluded (protocol); the study will conduct a within-trial HEA. The perspective is not specified, but the authors describe direct medical costs and discuss intervention costs, such as digital equipment (e.g., tablet, scale) and HCPs’ project working hours (e.g., patient follow-up via Dignio). |
| **eRAPID**  ***Intervention:*** Symptom/AE reporting & alert platform  ***Publications****:* Trial protocols^9,10^, RCT^11^, HTA^12^, HEA^13^  ***Setting:*** UK, 2017-2024  ***Cancers:*** Colorectal, breast, gynecologic, pelvic  ***Treatment:*** Systemic anticancer or radiation therapy  ***Sample:*** N = 508 (eRAPID = 256, Usual Care = 252) | ***Instruments:*** Novel (eRapid)  ***Symptom scale:*** 0 = none, 1 = mild, 2 = moderate, 3 = severe.  ***Algorithm:*** no problems reported = no advice, mild/moderate symptoms = self-management advice, ≥3 moderate problems or improved severe = contact team when convenient, severe = contact hospital now (activated emergency alerts to the nursing team). | ***ePROM completion:*** *weekly*  ***Methods:*** The study calculates time off work using the human capital method in the societal perspective, and estimates time spent on ePROMS.  ***Results:*** Patients reported better self-efficacy and confidence in self-managing their symptoms, and improved HRQoL at the end of the chemotherapy. | Additional HCP time is not included in the analysis, but eRAPID is assumed to reduce patient-facing clinician time by reducing unnecessary consultations. | The system provides immediate severity-graded advice based on clinical algorithms to guide self-management or hospital contact. AE data are transferred to electronic patient records for review by HCPs. | QALY & cost differences were small and not statistically significant, but out-of-pocket costs were lower in eRAPID, indicating eRAPID may help patients access support needed within the NHS. |
| **eSMART**  ***Intervention:*** Advanced symptom management system (ASyMS)  ***Publications:*** Trial protocol^14^  ***Setting:*** Austria, Greece, Ireland, Norway, UK, 2017  ***Cancers:*** Breast, colorectal, lymphoma  ***Treatment:*** Commencing first-line chemotherapy  ***Sample:*** 1,108 total target | ***Instruments*:** ASyMS, CTAQ  ***Symptom scale:***  *Severity* = [mild, moderate, severe];  *Distress* = [not at all, a little, quite a bit, very much]  ***Algorithm:*** Severity descriptions are based on CTCAE. ePROMs are recorded in a secure server, from which clinically concerning reports are processed into alerts (amber = 8-hour response, red = 30-minute response). | ***ePROM completion:*** daily  ***Design:*** Patients receive ASyMS handset and a tympanic thermometer. Each time, they (1) record their location (home/hospital), (2) indicate the presence of each symptom (yes/no) + severity + distress level, and (3) record their temperature. Patients immediately receive automated, tailored self-care advice, HCP & support contact details, and symptom data visualization.  ***Assumptions:*** ASyMS is hypothesized to increase patients' HRHRQoL and self-care, while reducing their symptom burden, supportive care needs, anxiety, and work limitations. | HCPs take shifts as the dedicated 'alert handler'. An alert handler will be active at all times and receive alerts on a dedicated ASyMS clinician handset. HCPs will review real-time ePROMs, contact patients, and initiate interventions. | Patient & HCP training is described in detail by the authors, along with information technology systems. Both receive ASyMS handsets & instructions of use, including protocols in case of technical failures. HCPs receive pre-recruitment training sessions. No details given on including training & set-up costs in the HEA. Changes in clinical practice as a result of the ASyMS will be evaluated. | Not concluded (protocol); the study will conduct a within-trial HEA. Perspective and other parameters not specified. |
| **Ferre et al. (2021)**  ***Intervention:*** Web-based data  Collection, monitoring, and supportive automatic analysis.  ***Publications:*** Cohort study protocol^15^  ***Setting:*** Italy 2021  ***Cancers:*** Breast  ***Treatment:*** Mastectomy and immediate breast reconstruction  ***Sample:*** 220 women | ***Instruments*:** BREAST-Q  ***Algorithm:*** No alerts or ePROM-based decision algorithms are described. Instead, ePROM data is analyzed automatically, giving HCPs supplementary information in care provision. | ***ePROM completion:*** specific timepoints (baseline, 3m, 1y)  ***Design:*** Surveys are sent directly to the patients via email or text message, containing a link to the questionnaires. | HCPs receive dashboards (tables and bar charts) generated from ePROMs to help inform decision-making and performance/quality discussions. | Authors discussed the intervention's dependence on HCPs' need to adequately inform and enroll patients, along with institutional levers to incentivize HCPs' cooperation. | The study will not conduct a full HEA, but will collect and report limited HRU (surgical) data. |
| **ICARE-P**  ***Intervention:*** Online holistic needs assessment, linking the patient and the health care team  ***Publications:*** Feasibility^16^  ***Setting:*** UK, 2024  ***Cancers:*** Prostate  ***Treatment:*** Community-based, stable prostate clinical care pathway  ***Sample:*** N = 160 (intervention = 125, control = 35) | ***Instruments*:** HNA, EORTC (QLQ-C30)  ***Algorithm:*** This system shows a “red flag” if a patient reports having any physical or psychological symptoms of serious concern (e.g., suicidal thoughts, urinal bleeding). | ***ePROM completion:*** baseline, 3 & 6 months  ***Design:*** After the ePROM assessment, patients complete a personalized care plan with a nurse. "Red flag" system alerts HCPs and prompts the patient to urgently see a GP. | HCPs can access ePROMs and specific "Red flag" system alerts. GPs & practice nurses are encouraged to perform opportunistic reinforcement for agreed actions by surgery patients. | The system provides structured access to assessment summaries and care plans for HCPs. After patients complete ePROMs, the system autocompletes care plan sections. Intervention-specific training is provided to primary care-based nurses and an IT peer supporter. | Not concluded; the HEA will consider HRU data & completion rates, hospital/community/social care referrals. |
| **Kaiku Health**  ***Intervention:*** Real-time remote symptom monitoring and management  ***Publications:*** Pilot/ implementation^17^  ***Setting:*** Germany, 2020  ***Cancers:*** Lung  ***Treatment:*** Immunotherapy (monotherapy or with chemotherapy)  ***Sample:*** N = 51 survey respondents | ***Instruments*:** PRO-CTCAE  ***Symptom scale:*** 0 = none, 1 = mild, 2 = moderate, 3 = severe, 4 = very severe  ***Algorithm:*** Alerts triggered by symptoms are mentioned by authors, but the details and algorithm are not discussed. | ***ePROM completion:*** weekly ***Results:*** Remote monitoring empowered patients and increased their feeling of safety during treatment. Patients spent a median of 3.5 hours viewing self-help resources. Some patients reported reduced unscheduled outpatient appointments and telephone consultations due to remote monitoring. | HCPs found the tool improved efficiency and was well integrated into their daily clinical workflow. HCPs invested up to 30 minutes in introducing patients to the tool, but half of them saved 5 minutes or more per consultation, leading to long-term time saving. | Time savings were sometimes compromised by interoperability issues, such as a lack of tool integration into the IT system of the clinic and, consequently, data having to be gathered from both the web-based Kaiku Health platform and the clinic's IT system. | The authors conducted survey research to investigate time use by patients & HCPs, which is useful for economic modelling. Time-use-related surveys use quantitative ordinal variables, without qualitative summaries. |
| **LYSA**  ***Intervention:*** Nurse‑led clinic targeting symptom management through ePROM-based alerts  ***Publications:*** Trial protocol^18^  ***Setting:*** Ireland, 2022  ***Cancers:*** breast, gynecologic  ***Treatment:*** Within 12 months of completion of primary curative therapy  ***Sample:*** 200 total target | ***Instruments*:** PRO-CTCAE, PROMIS  ***Symptom scale:*** 1 = Not at all, 2 = A little bit, 3 = Somewhat, 4 = Quite a bit, 5 = Very much  ***Algorithm:*** Specific thresholds/triggers are not specified by the authors. Triggers notify the nurse and/or dietician. High PRO-CTCAE responses = worse functioning, high PROMIS T-score = more symptoms. | ***Design:*** Patients receive study resources and pathway information based on baseline ePROMs. Then, they may complete regular ePROMs between routine care consultations and trigger alerts. | Triggers notify the nurse and/or dietician, prompting evaluation and management in between routine clinic visits. Support can include self-help, advice in-clinic, or referral. | The system specifies symptom-specific pathways following trigger alerts. Patients who do not trigger any alerts are followed up to confirm their healthy status. | Not concluded (protocol); the study will conduct a within-trial HEA. The perspective, or other detailed HEA parameters and assumptions, are not discussed. |
| **MyPal**  ***Intervention:*** ePlatform with spontaneous symptoms reporting, self-help, notifications  ***Publications:*** Implementation^19^  ***Setting:*** Greece, Italy, Czech Republic, Sweden, 2021  ***Cancers:*** All (blood)  ***Treatment:*** Standard treatment for chronic lymphocytic leukemia or small lymphocytic lymphoma  ***Sample:*** N/A | ***Instruments*:** ESAS, BPI, Distress thermometer  ***Algorithm:*** Exact algorithm not presented. Authors state that MyPal notifies HCPs of completed or missed ePROMs by the patients. | ***ePROM completion:*** weekly  ***Design:*** In addition to completing ePROMs, patients are notified by the MyPal mobile app to complete monthly HRQoL surveys, as well as study support surveys for measuring engagement and treatment support surveys for measuring risk factors for non-adherence at three time points (baseline, 3 months, 6 months). Patients can use MyPal as a self-help search engine and to enter medications & reminders. | HCPs' MyPal functions include receiving notifications, viewing aggregated & individual ePROM dashboards, a discussion tool, CRF cost form (for HEA), and uploading medical documents. | MyPal infrastructure includes a database to store MyPal data, an authentication server, and a clinical site-specific administrator who adds new users. MyPal, as a decision aid, is intended to recognize clinical complications earlier, improve patients’ knowledge, reduce anxiety, promote shared decision-making, and reduce decision conflicts. | The authors mention a planned within-study HEA, but the perspective or other details are unspecified. It will be important to observe how the authors approach conducting a HEA of an implementation study (rather than, e.g., an RCT). |
| **Oncokompas**  ***Intervention:* e**Health self-management app to support cancer survivors in monitoring HRQoL  ***Publications:*** RCT  ***Setting:*** The Netherlands, 2021  ***Cancers:*** Head & neck, colorectal, breast, lymphoma  ***Treatment:*** None/single (<45%) or multimodal (>55%)  ***Sample:*** N = 625 (intervention = 320, control = 305) | ***Instruments*:** Novel (Oncokompas)  Symptom scales and exact alert/decision algorithms are not presented. However, the authors discuss algorithms linked to tailored information and advice, stating that self-help options are provided for elevated well-being risks, while professional healthcare options are offered for seriously elevated risks. | The frequency of reporting is not specified, but the authors state that Oncokompas can be used independently of HCPs, implying as-needed use. After completing ePROMs, patients receive tailored advice based on ePROMs, self-help, and further healthcare for serious risks. Patients can also indicate topics they would like to address. | HCPs offer professional healthcare options only following seriously elevated well-being risks. Otherwise, self-help options are prioritized/presumed. | Oncokompas was developed according to a participatory design approach and consists of 3 modules: (1) Measure (collect ePROMs), (2) Learn (real-time ePROM analysis), and (3) Act (overview of healthcare options) | Societal perspective HEA: Oncokompas is more or less equally effective in terms of utilities and costs as usual. Findings potentially limited by EQ-5D ceiling effects and a lack of long-term potential cost-savings. |
| **PhONEME/Interaktor**  ***Intervention:*** ePROM app for supportive care during cancer treatment  ***Publications:*** HEA^20^  ***Setting:*** Sweden, 2025  ***Cancers:*** Breast, prostate  ***Treatment:*** The study contains two RCTs. B-RCT: neoadjuvant chemotherapy; P-RCT: radiotherapy.  ***Sample:*** N = 299 [B-RCT (N = 149 breast cancer patients, intervention = 74, control = 75) and P-RCT (N = 150 prostate cancer patients, n = 75 per group)] | ***Instruments*:** Novel (PhONEME/Interaktor)  ***Symptom scale:*** 1 = not at all, 2 = a little, 3 = somewhat, 4 = very much  ***Algorithm:*** The exact algorithm is not presented. Nurses manage alerts and are automatically classified into yellow (1-hour response) and red (1-day response) by a built-in risk assessment model for symptoms of concern. | ***ePROM completion:*** daily (weekdays)  ***Design:*** Patients use Interaktor to fill in ePROMs, view symptom graphs, and access self-care information.  ***Results:*** Patients gain a small QALY improvement by using the app. | Nurses view ePROMs via the interface, receive SMS alerts to which they respond within 1 hour or 1 day (depending on severity). Additional expenses for nurses handling alerts were not included based on unpublished qualitative research. | A researcher was available to assist with any technical questions or issues. The system sent reminders to patients who did not fill out forms. | The study presents a healthcare payer perspective HEA. ePROM and interactive support via an app generated a small improvement in QALYs at a low intervention cost and may be cost-effective, depending on the costs considered. Considerable variability in patient health care costs introduced uncertainty around the estimates, preventing a robust determination of cost-effectiveness. |
| **PRISMS**  ***Intervention:*** Telehealth  monitoring of chemotherapy side-effects  ***Publications:*** Trial protocol^21^  ***Setting:*** Australia, 2015  ***Cancers:*** Lymphoma, leukemia  ***Treatment:* ≥**2 cycles of chemotherapy + self-help resources, contact to HCPs  ***Sample:*** 100 per group target | ***Instruments*:** Novel (PRISMS), ASyMS, C-SAS  ***Symptom scale:***  *Severity* = [mild, moderate, severe];  *Distress* = [not at all, a little, quite a bit, very much]  ***Algorithm:*** Severity descriptions are based on CTCAE. ePROMs are recorded in a secure server, from which clinically concerning reports are processed into alerts (amber = 8-hour response, red = 15-minute response). Non-completion = 'missing alert' & 8-hour response. | ***ePROM completion:*** twice/day  ***Design:*** Patients receive an Android tablet with remote monitoring enabled. Patients can complete questionnaires when prompted or at will. After completing ePROMs, patients obtain tailored self-care and symptom graphs.  ***Assumptions:*** PRISMS is hypothesized to lower patients' nausea, mucositis, constipation, and fatigue symptoms. | Intervention nurses receive email alerts on a dedicated mobile phone (monitored at all times), access tabulated and graphical ePROM data, contact patients, and record actions based on triage and assessment algorithms included in the intervention. PRISMS includes structured nursing support (6 in-person or phone consultations). | The ePROM tool was based on ASyMS and developed based on consultation with hematology nurses, clinicians, and patients. The authors also discuss diffusion (ePROM arm HCPs influencing control arm care). | Not concluded (protocol); the study will conduct a within-trial HEA from both healthcare and societal perspectives. The authors discuss intervention delivery costs, which will be sourced from research team records (e.g., PRISMS website, data transmission costs, training time/materials), excluding R&D costs. |
| **PROMISE**  ***Intervention:*** Combined symptom/distress tracking, alerts, and management pathways  ***Publications:*** Protocol (effectiveness & implementation trial)^22^  ***Setting:*** Australia, 2024  ***Cancers:*** All (solid)  ***Treatment:*** Systemic anticancer or radiation therapy  ***Sample:*** 286 per group target | ***Instruments*:** Distress thermometer, ESAS  ***Symptom scale:*** 0 = no distress, 10 or 100 = extreme/worst possible symptom.  ***Algorithm:*** Hospital-specific protocols codesigned with HCPs set out alert frequency, thresholds, pathways, and actions/recommendations. Alerts are automatically grouped by urgency (low, medium, high, urgent). | ***ePROM completion:*** weekly, fortnightly, or monthly  **Hypotheses:** routine self-reporting of ePROMs will lower symptom burden, unmet supportive care, and HRU (emergency use & unplanned hospitalization, outpatient visits), but improve HR-HRQoL and survival. | Alerts are routinely monitored by HCPs. The clinical team also receives contact from patients reporting urgent symptoms outside monitoring hours, who receive a contact instruction. | If the symptom score > threshold, the system alerts the relevant discipline. Mixed methods using the RE-AIM framework will be used to assess organizational, implementation, and adoption aspects. This includes ePROM completion rates, frequency of clinician access to ePROM data, outpatient appointment length, proportions of HCPs/centers using ePROMs at study end, etc. | Not concluded (protocol); the study will conduct a within-trial HEA from a healthcare perspective. |
| **PROMPT-Care**  ***Intervention:*** ePROM monitoring, alert, and self-help system  ***Publications:*** Nonrandomized trial^23^  ***Setting:*** Australia, 2020  ***Cancers:*** All (solid)  ***Treatment:*** Active treatment or follow-up care.  ***Sample:*** PROMPT-Care = 328, Control = 1911, Matched Control = 1312 | ***Instruments*:** Distress thermometer, ESAS, SCNS-ST9  ***Symptom scale:*** 0 = worse, 10 = better  ***Algorithm:*** No central algorithm is provided. Instead, each participating site followed its cancer center’s standard clinical care pathway in response to alerts. | ***ePROM completion:*** monthly  ***Design:*** In addition to symptoms and unmet needs, patients complete a problem checklist and access domain-specific self-management resources related to the above-threshold ePROMs. | A designated HCP reviews email alerts at least 1/day. With an average of 31 alerts per month, reviewing alerts fits within the existing workflows of HCPs without too much extra burden. | The automated clinical alerts prompted the majority of clinical report reviews, highlighting the importance of embedded triggers for above-threshold ePROMs. | No cost or cost-utility analysis was provided, but the authors report a detailed HRU breakdown useful for extraction. Emergency visits were significantly lower by 33% in the intervention group, but no difference in chemotherapy time or allied health referrals. |
| **PRO-NET**  ***Intervention:*** Symptom tracking, integrated with reactive alerts and nudges  ***Publications:*** Trial protocol^24^  ***Setting:*** China, 2025  ***Cancers:*** Lung  ***Treatment:*** Immunotherapy  ***Sample:*** 150 per group target | ***Instruments*:** PRO-CTCAE, PROMIS  ***Symptom scale:*** 0 = none, 5 = as worst as you can imagine  ***Algorithm:*** Alert is triggered if symptom grade is ≥3, or if symptom worsens by ≥2 grades in two consecutive weeks, or if 'chills/fever' or 'fallen' grade >0. | ***ePROM completion:*** fortnightly  ***Design:*** Patients receive alerts and advice, and are responsible for initiating the following action (reactive approach) | The role of HCPs in reading or responding to alerts is not explicitly clarified, but "actionable information for clinicians to evaluate whether a symptom requires further investigation" is based on using a consistent severity measure throughout the ePROMs. | The intervention uses a reactive approach by sending alerts to patients (as opposed to 'active' sending alerts to clinicians), as this is assumed to lower intervention costs. | Not concluded (protocol); the study will conduct a within-trial HEA from a healthcare & societal perspective. |
| **Sentinel**  ***Intervention:*** ePROM monitoring during routine cancer treatment  ***Publications:*** RCT^25^, HEA^26^  ***Setting:*** France, 2017-2019  ***Cancers:*** Lung  ***Treatment:*** Surgery or radio/chemotherapy <3 months before randomization  ***Sample:*** N = 121 (experimental = 60, control = 61) | ***Instruments*:** Novel (Sentinel)  ***Symptom scale:*** Some symptoms (e.g., fatigue) were graded by severity (0 = no symptom to 3 = major symptoms), while others (e.g., swelling) were indicated by a yes/no variable.  ***Algorithm:*** ePROMs triggers alert if: weight loss ≥3kg/2 weeks, ≥2 grade-3 symptoms, or total score ≥6. Then, the nurse calls the patient to confirm symptoms. Upon confirmation, the oncologist calls the patient and arranges an in-clinic visit within 8 days if needed. | ***ePROM completion:*** weekly  ***Design:*** ePROM arm patients do not follow the CT scan regular schedule in the comparator arm.  ***Assumptions:*** Patients benefit from earlier relapse detection by reporting ePROMs.  ***Results:*** Intervention effectiveness varied with planned visits in the subgroup analysis: improved survival for IIIB/IV patients (3-monthly visits), but not the maintenance therapy group (3-weekly visits). More patients attended unscheduled visits in the experimental arm than in placebo (58.3% vs 24.6%). | HCPs receive a table of color-coded symptom scores and alerts, based on which they make decisions on follow-up. The mean weekly time spent by the oncologist to manage all the web alerts was 15 minutes for 60 simultaneous users. Presence or role of decision support not discussed. | Study staff give 5 minutes to participating patients, who are provided with login details. In the experimental arm, CT scans were less frequent than in the control due to reliable relapse detection via ePROM reports. Implementation, set-up, and training are not discussed in detail. The e-PROM system cost was estimated at €200 per patient per month, covering technical components and HCP time use, based on similar systems and prior studies. | ePROMs reduced the follow-up costs and are a cost-effective strategy. There were more consultations in the ePROM arm (193) vs control (133), but fewer CT/PET-CT scans (83 vs 90). ePROMs resulted in more round trips vs control (257 vs 173), but more transport autonomy (private care & public transport vs taxis & ambulances). OWSA shows results sensitive to ePROM system costs, end-of-life care (obtained from prior research), and anti-cancer treatment costs. The authors report a healthcare perspective despite transport cost inclusion. |
| **SIS.NET**  ***Intervention:*** ePROM-based symptom management & alerts  ***Publications:*** RCT^27^  ***Setting:*** USA, 2016  ***Cancers:*** Breast  ***Treatment:*** 6 months out from chemotherapy completion, 3 months out from hormonal therapy initiation, and 3 months out from surgery and radiotherapy.  ***Sample:*** N = 100 (SIS.NET = 59, control = 41) | ***Instruments*:** SF-36, PHQ-8, MSAS  Symptom scales and algorithms for alerts or automated decisions are present but not discussed in detail. | ***ePROM completion:*** 3-monthly  ***Design:*** Patients attended 3 routine follow-up visits, between which they received email reminders to complete ePROMs and could request interim questionnaires for new symptoms between the 3-monthly regular completions. | HCPs receive auto-generated symptom reports, based on which HCPs call patients to discuss any new or changed symptoms based on a comparison with the baseline and suggest further action. HCPs reviewed ePROMs before the clinic appointment. | ePROMs can also lead to support resource auto-referrals. Authors discuss that ePROMs may help patients report symptoms, which may allow for additional opportunities to improve symptom management and HRQoL, at the cost of additional HRU. | No HEA was conducted, but the study hypothesized that SIS.NET would reduce HRU (specifically cancer-related clinic visits), but this was not supported by the results, which showed no statistically significant difference. However, SIS.NET patients reported more symptoms vs SOC (7.36 vs 3.2) overall. The study contains extractable HRU data for economic modelling. |
| **STAR**  ***Intervention:*** ePROM monitoring & alert system during routine outpatient chemotherapy  ***Publications:*** RCT^28^, HEA (model)^29^  ***Setting:*** USA, 2016  ***Cancers:*** All (solid)  ***Treatment:*** Routine outpatient chemotherapy for advanced solid tumors  ***Sample:*** N = 766 (STAR = 441, usual care = 325); Subgroups: IT experienced = 539, IT inexperienced = 227. | ***Instruments*:** Novel (STAR)  ***Symptom scale:*** 0 = not present, 4 = disabling  ***Algorithm:*** STAR triggered e-mail alerts to nurses whenever a patient-reported symptom worsened by ≥ 2 points or reached an absolute grade ≥ 3.  ***Model:*** 2-state (dead & alive) Markov model with 6-month cycles and a 10-year (lifetime) horizon. Transition, utility, and HRU were sourced from a previous study.^28^ | ***ePROM completion:*** weekly (IT experienced) or in-clinic/non-remote (IT inexperienced)  ***Design:*** Patients received training before commencing (e.g., call about concerning symptoms outside of business hours, when ePROMs are not monitored).  ***Results (RCT):*** On average, 73% of STAR participants completed ePROMs at any given clinic visit. Patient’s HRQoL was improved.  ***Model:*** The model assumed a 70% uptake rate of STAR. | ***RCT:*** No specific guidance for HCPs on responses to ePROMs, so discretion was used. Authors report percentage breakdowns of the types of actions (e.g., phone counselling, onward referral) taken in response to ePROMs. No difference in the number of nurse calls was reported between arms.  ***Model:*** The model assumed that HCPs receive ePROM symptom reports printed out at regular clinic visits, based on Basch et al. (2016).^28^ | At enrolment, nonclinical study staff trained participants to use STAR. After business hours, emails were not monitored, and patients were encouraged to call about concerning symptoms. STAR did not allow skipped questions or free-text responses. The authors tabulate symptom scores by grade and conclude that ePROMs augment existing symptom management mechanisms. | ***RCT:*** No full HEA was provided, but the authors report a detailed HRU breakdown, which is useful for extraction. STAR resulted in better HRQoL, fewer emergency visits, fewer hospitalizations, a longer duration of palliative chemotherapy, and superior quality-adjusted survival.  ***Model:*** With a cost increase of CA$3,360, the use of a PRO tool for symptom monitoring yields an additional 0.25 QALYs, which is cost-effective under a CA$50K WTP (healthcare payer perspective) |
| **SYMPRO-Lung**  ***Intervention:*** Symptom tracking with one of two alert approaches (active and reactive)  ***Publications:*** Trial protocol^30^  ***Setting:*** The Netherlands, 2021  ***Cancers:*** Lung  ***Treatment:*** Starting radiotherapy, surgery, chemotherapy, immunotherapy, and/or targeted therapy.  ***Sample:*** 84 per group target | ***Instruments*:** PRO-CTCAE  ***Symptom scale:*** 0 = Not at all, 1 = A little bit, 2 = Somewhat, 3 = Quite a bit, 4 = Very much  ***Algorithm:*** An alert is triggered if symptom grade is ≥3, or if symptom worsens by ≥2 grades in two consecutive weeks, or if non-PRO-CTCAE triggers (fever, hemoptysis, weight change, loss of ≥3 kg in 1 or 2 weeks) are recorded. | ***ePROM completion:*** at least 1/week and at most 1/day  ***Design:*** Patients are encouraged to use a web-app to complete ePROMs (enabled via smartphone, laptop, computer, or tablet). Adherence is encouraged by reminders through a push notification or email. Patients can download their ePROM reports. | HCPs receive a report of weekly ePROMs, including graphs of symptoms over time, an overview of the symptoms that triggered an alert, the sum of symptom scores, and responses to symptoms. The HCPs take and record actions to trigger alerts (e.g., no action, extra phone consultation, onward referral). | The system contains two approaches to alert handling: active (HCPs instructed to contact patients via email and to provide triage, advice, or intervention) and reactive (patients advised to contact HCP via notification). | Not concluded (protocol); the study will conduct a within-trial HEA. Perspective and other parameters not specified, but using “clinical healthcare costs data” indicates healthcare payer.  The cost of intervention is mentioned as part of HEA, but not specified in detail - this could show active vs reactive differences. |
| **The Programme**  ***Intervention:*** Personalized survivorship care through remote monitoring and self-help  ***Publications:*** Historical control^31^  ***Setting:*** UK, 2019  ***Cancers:*** Prostate  ***Treatment:*** Radical prostatectomy, radiotherapy, or primary androgen deprivation therapy  ***Sample:*** N = 627 (the Programme = 293, comparator = 334) | ***Instruments*:** Holistic Needs Assessment (Health MOT)  ***Algorithm:*** The authors do not discuss the Health MOT or action triggers in detail. | ***ePROM completion:*** at baseline, 6 months after treatment, and then annually  ***Design:*** Men used the online system to view test results, personal information, treatment summaries, and care plans. On the online system, men completed a Holistic Needs Assessment (Health MOT) and messaged HCPs. | Support worker manages the Programme day-to-day and acts as a first point of contact. The team reviews Health MOTs during ‘virtual clinics’, and contact is made with the man if there is any concern. | Men attend a 4-hour face-to-face group self-management workshop to prepare them for the Programme. There are no scheduled urology/oncology follow-up appointments; Instead, follow-up care is facilitated through a bespoke digital system. Authors discuss provisions in the system for men who do not use IT. | The authors report a within-study cost-consequence analysis, which found that the cost of delivering the Programme was slightly greater than standard care, but reduced service use offset these additional expenses. However, the authors state that an evaluation of costs over a longer time period would spread the upfront costs of the Programme. |
| **VBHC-initiative**  ***Intervention:*** A supportive ePROM integration platform  ***Publications:*** Pilot/ implementation^32^  ***Setting:*** The Netherlands, 2019  ***Cancers:*** Breast  ***Treatment:*** Neoadjuvant systemic treatment + mastectomy + autologous breast reconstruction.  ***Sample:*** N = 239 patients enrolled | ***Instruments*:** EORTC-QLQ-Core, BREAST-Q, EORTC-QLQ Breast  Cancer, EQ-5D-5L, Distress Thermometer, CarerHRQoL-7D  ***Symptom scale:*** 0 = worse, 100 = better  ***Algorithm:*** No alerts or ePROM-based decision algorithms are described. | ***ePROM completion:*** baseline, following the last course of neoadjuvant systemic therapy, 6 months after surgery, and annually post-surgery  Patients report that the use of ***Results:*** ePROMs helps them to prepare for the upcoming appointment, which makes it more tailored to their needs. 20 minutes per patient were needed to complete ePROMs. | ***Design:*** HCPs use the ePROM system as a diagnostic tool, change care strategy or intervention, and select treatment pathways.  ***Results:*** HCPs reported that ePROMs provide a more complete view of care provision. | The authors developed an in-house open-source ePROM tool, which was linked to the EHR, enabling the review of the collected data for individual patients at the outpatient clinic. The tool sent email notifications to patients to complete ePROMs. | The authors discussed value-based care and generic measure comparison, but did not conduct a HEA. However, the authors collected disease-specific ePROM data alongside generic EQ-5D-5L data, which (provided data sharing by the authors) could be used to produce a Dutch mapping algorithm from included measures into EQ-5D health state utility. |
| **YES**  ***Intervention:*** Interactive portal for self-monitoring and managing concerns and symptoms outside of a clinical setting  ***Publications:*** Trial protocol^33^  ***Setting:*** USA, 2025  ***Cancers:*** Breast  ***Treatment:*** Usual care for female patients (age 15–39, stage 0-III breast cancer)  ***Sample:*** N/A | ***Instruments*:** PRO-CTCAE, PHQ-9  ***Algorithm:*** PHQ-9 responses are monitored by pre-programmed automatic alerts. If PHQ-9 ≥ 15/27 = HCPs follow up within 1 business day; if suicidal ideation is reported, staff notify principal investigator/covering clinician, then HCPs review EHR, call participants as soon as possible, and triage if necessary. PRO-CTCAE is for self-monitoring only and does not trigger alerts. | ***ePROM completion:*** monthly  ***Design:*** Patients can use YES to self-monitor symptoms over time, access chatrooms, and view self-help information.  ***Assumptions:*** YES is hypothesized to improve QOL and adolescent & young adult breast cancer concerns and symptoms. | HCPs only monitor and respond to psychosocial distress & suicidal ideation, not PRO-CTCAE symptoms. | Optional features include participant chatroom journaling, participant-triggered self-help & advice. The system generates ePROM trend graphs. Patients are prompted and reminded by text messages, mobile push notifications, and/or email. Limited discussion of set-up, implementation, or training. | The study will collect and report EQ-5D-5L data by trial arm, which will be useful for modelling parametrization (QALYs). No HEA components aside from this are discussed. |

(N)RCT = (Non-)Randomized Controlled Trial, HEA = Health Economic Analysis, HTA = Health Technology Assessment [report], AE = Adverse Event, HCP = healthcare professional, ePROMs = electronic Patient-Reported Outcomes, WTP = Willingness-to-Pay, IT = Information Technology, QALY = Quality-Adjusted Life-Year, OWSA, SLR, HRQoL, NHS = National Health Service,

## Summaries of cost-effectiveness results

The eRAPID intervention was acceptable to patients and staff, associated with better HRQoL and self-efficacy in patients receiving curative systemic treatment, and shown to have the potential for dominant cost-effectiveness for the UK NHS. ^13^ The uncertainty is due to small differences in costs and HRQoL compared to standard care, and high variability around the ICER, with cost-effectiveness probability estimates above 50% but below 60% at UK Willingness-to-Pay (WTP) thresholds of £20K-30K/QALY.

The Sentinel study showed a strong QALY and cost increase in the ePROM arm, yielding a low ICER of €20.9K/QALY and a high probability of cost-effectiveness (>95%) at €30K/QALY and €90K/QALY WTP thresholds. However, these figures include transport costs despite specifying a healthcare payer perspective. ^26^ Furthermore, Sentinel presented an interesting finding suggesting that ePROM-arm patients’ travel modes were more autonomous and cheaper (e.g., more walking, driving) versus control (e.g., more ambulance and taxi trips). ^26^

The STAR study demonstrated reductions in emergency visits and improvements in HRQoL for the ePROM arm, although the benefits were greater in the computer-inexperienced subgroup, which completed symptom reports in-clinic instead of at home; the authors explained that computer-inexperienced patients had worse overall health communication skills and therefore benefited more from a structured program. ^28^ The STAR-based model developed by Nixon et al. (2018) reported a high probability of cost-effectiveness (>90%) and a low ICER of CA$13. 5K relative to the Canadian WTP threshold of CA$50K/QALY. ^28,34^

The PHONEME/Interaktor study found its intervention to increase QALYs in both its breast and colorectal cancer groups. ^20^ However, it concurrently observed a high variability in costs and therefore ICERs, which range from €13.2K/QALY to €202.3K/QALY. The authors compared the ICERs against Swedish WTP definitions (“low” ≤ €9.7K/QALY, “high” ≥ €48.4K/QALY, “very high” ≥ €96.8K/QALY) but did not produce Cost-Effectiveness Acceptability Curves (CEACs) to calculate probabilities of cost-effectiveness. ^20^

The Oncokompas study reported positive but uncertain results; the base case ICER results pointed to the ePROM strategy being dominant relative to standard care, but confidence intervals for cost and QALY differences both encompassed 0. The authors did not present cost-effectiveness acceptability curves or conduct any WTP analysis. However, they performed a probabilistic analysis showing that Oncokompas has a 60% probability of being more effective, a 73% probability of being less costly, and a 47% probability of being both more effective and less costly.

The five studies with promising results but lacking HEA completeness are Capri, Affordable Better, the Programme, PROMPT-CARE, and SIS.NET. ^2,7,23,27,31^ The Capri within-trial budget impact analysis demonstrated the intervention to be cost-saving from a healthcare perspective. ^7^ Given the intervention’s RCT demonstrated positive effects on PREMs, hospitalizations, and toxicity, a full HEA would likely find it to be a dominant strategy relative to standard care. ^6^ The Affordable Better study estimated a €22.4K cohort cost-saving from ePROM monitoring over one year compared to standard care, attributed in part to greater self-organization of care. ^2^ Furthermore, the intervention’s patients experienced higher treatment satisfaction and lower worry about recurrence following its use, which could also translate into higher QALYs and therefore a dominating strategy within a full HEA. ^2^ Similarly, the Programme study reported statistically significant improvements in measures addressing unmet needs and improving HRQoL, as well as a per-participant 8-month cost savings of £38.6 compared to standard care. ^31^ PROMPT-CARE reported a 33% reduction in emergency hospitalizations in the ePROM arm relative to matched controls, alongside a high likelihood of adoption and implementation in routine care, but no associations in allied health referrals or chemotherapy duration. ^23^

The SIS.NET study reported no statistically significant differences between the ePROM and standard care arms in several health resource use categories, including oncology-related appointments, physician visits, and tests/scans. The authors concluded that integration of ePROM monitoring facilitated symptom reporting and may provide a means of convenient symptom assessment, despite the lack of HRU impact. ^27^

## Summary of qualitative research components

Supplementary Table 2 categorizes information into the following columns: (1) “Study & Methods”, (2) “Aim(s)”, (3) “Themes”, and (4) “Conclusion”. The first column describes the study/intervention name, the qualitative methods employed by the study (e.g., semi-structured interviews, thematic analysis), and the sample populations of qualitative research participants. All 12 studies specified the qualitative methods used, but only 5 studies specified the achieved or intended qualitative-specific number of participants. The middle two columns list the aims and themes of the qualitative components, while the final column summarizes qualitative summaries reported in completed studies.

Table 2: Summaries of qualitative research components within included studies

| Study & Methods | Aim(s) | Themes | Conclusion |
| --- | --- | --- | --- |
| **eRAPID:**  semi-structured interviews, feedback forms (patients = 45, HCPs = 19) | Gathering patient and staff feedback to explore how the system was used, received, and influenced cancer care, providing context to the trial’s main findings. | user experience, acceptability, personal value, impact on care | eRAPID is easy, accessible, and useful. It improves patient-hospital connection, HCPs' tracking symptoms over time, and identifying issues not otherwise discussed. Staff feedback on eRAPID's impact on consultation time was mixed. A mobile app and expanded HCP and patient training could improve eRAPID further. |
| **PRO-NET:**  semi-structured interviews (patients, HCPs, n = N/A) | Understanding patient preferences for the frequent collections of PROs to minimize the potential burden; assessing patients’ responses to alerts, self-management, satisfaction, burden, benefits, adherence, and user-friendliness, while also identifying barriers, facilitators, sustainability, and scalability of PRO-NET | data collection frequency, responses to alerts, self-management, adherence/adoption, user experience, sustainability, scalability | [not concluded]  Preliminary interviews led to having only one measure across symptoms (severity) to reduce patient burden and simplify decision-making |
| **PROMISE:**  semi-structured interviews (patients, HCPs, carers; n = 30) | Assessing RE-AIM indicators (Reach, Adoption, Implementation and Maintenance), reasons for successes and implementation challenges. | user experience, adherence/adoption, implementation | [not concluded] |
| **eHealth@Hospital-2-Home:** interviews (unspecified), thematic analysis (n = N/A) | Exploring the intervention experience and informing the results of the intervention effects and future implementation. | usability, acceptability | [not concluded] |
| **LYSA:**  semi-structured interviews, focus groups, thematic analysis (patients, HCPs, stakeholders, n = N/A) | Gathering opinions of involvement in the cancer survivorship clinic and discussing perceived facilitators and barriers to implementation. | user experience, implementation | [not concluded] |
| **SYMPRO-Lung:** think-aloud interviews, thematic analysis (patients, HCPs) | To evaluate the app and assess the implementation fidelity. | implementation fidelity | [not concluded] |
| **ICARE-P:**  semi-structured interviews, thematic analysis (patients = 10, HCPs = 8) | Determining variation in delivery and experience of usual care, identifying barriers/facilitators to intervention implementation, and assessing the views of patients & HCPs on HNA. | user experience, implementation, impact on care, self-management | Phase 1 demonstrates enthusiasm from patients & HCPs, including from men with little IT experience; designers responded to this by providing an Internet-enabled tablet computer to an IT assistant. GPs in particular expressed the importance of a brief output from the HNA; designers prioritized conciseness & interpretation. |
| **YES:**  semi-structured interview, exit interview (semi-structured: n = 30, exit: n = intervention arm patients) | Understanding how young breast cancer survivors prefer to self-manage survivorship needs, concerns, and symptoms, as well as understanding ways that the YES portal might support their self-management. | self-management | [not concluded] |
| **PhONEME/ Interaktor:** content analysis, interviews (unspecified) (N/A) | The authors used a conventional and summative approach to create HRU variables related to breast & prostate cancer treatments based on ICD codes. As part of separate (and unpublished) research, nurses were interviewed about the impact of alert handling on workloads. | HRU variable identification, responses to alerts, impact on workload/time use | HRU variables were composed of categories corresponding to groups of ICD codes and incorporated into the cost-effectiveness analysis. Additional expenses for nurses handling alerts were not included based on unpublished qualitative research. |
| **MyPal:** focus groups, participatory design, virtual validation workshops (patients = 9, HCPs = N/A) | To elaborate on the use cases and identify potential restrictions and end-user requirements of MyPal. Workshops validated the HCP web application. | user experience, usability, acceptability, design recommendations | There is broad enthusiasm among patients and HCPs for MyPal, but there is a need for usability, personalization, and feedback features. MyPal was modified based on end-user feedback: consistent menus, intuitive visual cues, and notification messages improve experience and engagement. |
| **Kaiku Health:** semi-structured interview, thematic analysis (nurses =13, physicians = 11, patients = 27) | Assessing ePROM adoption, impact on clinical care, alert system perceived usefulness, and perceived ease of use. | impact on workload/time use, adherence/adoption, impact on care, feasibility | User acceptance of the tool was high, with usefulness and communication being the most appreciated attributes. Participants indicated more efficient and focused communication between patients and HCPs. HCPs believed that the standalone tool was well integrated in their daily clinical workflow and improved efficiency within the health care team. Some HCPs reported time savings and reduced need for unscheduled outpatient appointments and telephone consultations. The tool showed high feasibility. |
| **VBHC-initiative:** interviews, focus groups, surveys (patients, HCPs, n = N/A) | Defining outcomes, selection and capturing multidisciplinary provider and patient perspectives. | N/A | Interviews with patients helped select the outcome questionnaires, but details on qualitative analysis are lacking. |

## ePROM Monitoring Costs

Any health economic model would have to include the costs of the intervention itself, not just the difference in health resource use costs between treatment strategies. In the context of ePROMs, we identified two cost categories: (1) initial or upfront setup costs, which could include the costs of information technology (IT), web/app development, providing equipment (e.g., ePROM-dedicated tablet), integrating the intervention into an organization, and training patients and HCPs in its use, and (2) regular maintenance costs, which could include wages of IT administration professionals and dedicated HCPs tasked with supervising or managing the ePROM platform. We found that cost categories are not consistently reported in the studies included in this review.

For available estimates, we presented rounded extracted costs alongside conversions to 2024 GBP in brackets using *exchangerates.org.uk* and Bank of England tools; we used the average calendar year values for both conversions, as specified by the tools. ^35,36^ We used generic consumer price indices (CPIs) over health-specific ones, because we judged ePROM monitoring costs to be composites of both health-related and non-health cost categories (e.g., software, hardware, staff, etc.). If the currency year was not reported, the publication year was used for conversions.

Nixon et al. (2018) list input costs provided by a company (Cambian®), with upfront setup costs of CA$193,000 (£141,011) and per-patient-year maintenance costs of CA$50 (£37). ^29^ The CAPRI authors report running program costs from two perspectives: ^7^

- *Healthcare payer* = €103/patient-month (£97): €75/month (£70) for the digital solution + €28/month (£26) for the care structure, including the nurse navigator’s salary, and
- *Societal* = €87/patient-month (£82) based on an annual subscription of €10,000 (£9,378) and the nurse navigator’s salary. However, the detailed calculations are unclear.

In the Sentinel study, ePROM system costs were estimated at €200 (£217) per patient-month, covering technical components and HCP time use, based on similar systems and prior studies. ^26^

Some studies reported time spent on the ePROM system by patients or HCPs, which could, in turn, be used as input to calculate running costs. In the VBHC-initiative, completing a set of ePROMs took 20 minutes per patient. ^32^ In Sentinel, the mean weekly time spent by the oncologist to manage all the web alerts was 15 minutes for 60 simultaneous users. ^26^

The Programme reported detailed cost breakdowns of screening and set-up, preparatory workshop delivery, and HCP & patient electronic messages. ^31^ The mean direct cost of the Programme (excluding service use) was £102 (£137) [SD = £21 (£28)], within which training workshops cost £63 (£82) [SD = £12 (£16)] per patient to deliver. ^31^

Except for The Programme, none of the reported results related to system setup or maintenance were accompanied by standard errors or confidence intervals, so future modelers using these input parameters would have to make assumptions about uncertainty in their sensitivity analyses. Furthermore, these costs are likely to vary widely across ePROM interventions, depending on existing infrastructure and organizational or health system contexts.

## Parameter Extraction

Table 3: Extractions of rates (events per person-month) and incidence rate ratios (IRRs)

| Event | ePROM | Control | IRR ± SE (95% CI), ePROM/Control |
| --- | --- | --- | --- |
| Affordable Better^2^ Colorectal cancer | | | |
| Tests/scans | 0.25 | - | - |
| - Carcinoembryonic antigen | 0.14 | - | - |
| - Ultrasound | 0.07 | - | - |
| - X-ray (chest) | 0.03 | - | - |
| - CT (thorax/abdomen) | 0.01 | - | - |
| Outpatient visits | 0.14 | - | - |
| - In-clinic | 0.00 | - | - |
| - Telephone | 0.02 | - | - |
| - ePROM app | 0.01 | - | - |
| - Remote | 0.11 | - | - |
| Parking (90 minutes) | 4×10^-3^ | - | - |
| Transport | 4×10^-3^ | - | - |
| CAPRI^7^ Multiple cancers | | | |
| Outpatient visits | 4.08 | 4.13 | 0.99 ± 0.02 (0.95, 1.03) |
| - General practice | 0.80 | 0.70 | 1.14 ± 0.05 (1.04, 1.25) |
| - Oncology | 0.73 | 0.71 | 1.03 ± 0.05 (0.94, 1.13) |
| - Oncology OCC | 0.09 | 0.09 | 1.00 ± 0.13 (0.77, 1.29) |
| - Other consultations | 0.09 | 0.11 | 0.82 ± 0.13 (0.64, 1.05) |
| - Nurse | 1.39 | 1.56 | 0.89 ± 0.03 (0.84, 0.95) |
| - Physiotherapy | 0.77 | 0.78 | 0.99 ± 0.04 (0.90, 1.08) |
| - Psychology | 0.16 | 0.15 | 1.07 ± 0.10 (0.88, 1.30) |
| - Nutritionist | 0.05 | 0.03 | 1.67 ± 0.20 (1.12, 2.48) |
| Emergency visits | 0.10 | 0.12 | 0.83 ± 0.12 (0.66, 1.05) |
| Hospitalizations | 0.10 | 0.13 | 0.77 ± 0.12 (0.61, 0.97) |
| Trips (one-way) | 1.07 | 1.27 | 0.84 ± 0.04 (0.78, 0.91) |
| - Ambulance | 0.12 | 0.14 | 0.86 ± 0.11 (0.69, 1.06) |
| - Light sanitary vehicle | 0.26 | 0.35 | 0.74 ± 0.07 (0.64, 0.86) |
| - Taxi | 0.69 | 0.78 | 0.88 ± 0.05 (0.81, 0.97) |
| Death (adjusted HR) | - | - | 1.16 ± 0.17 (0.83-1.61) |
| eRAPID^11,37^ Multiple cancers | | | |
| ePROM-triggered alerts | 3.45 | - | - |
| - No problems [no alert] | 0.11 | - | - |
| - Moderate | 2.83 | - | - |
| - Severe | 0.48 | - | - |
| - Emergency | 0.03 | - | - |
| PROMPT-Care^23^ Multiple cancers | | | |
| Emergency visits | 0.05 | 0.06 | 0.87 ± 0.06 (0.77, 0.98) |
| Sentinel^25,26^ Lung cancer | | | |
| Consultations | 0.31 | 0.22 | 1.38 ± 0.11 (1.11, 1.72) |
| Imaging tests | 0.26 | 0.27 | 0.96 ± 0.11 (0.77, 1.19) |
| Trips (round) | 0.41 | 0.29 | 1.41 ± 0.10 (1.17, 1.72) |
| - Ambulance | 0.00 | 0.02 | 0.17 ± 0.77 (0.04, 0.78) |
| - LSV/taxi | 0.19 | 0.16 | 1.21 ± 0.14 (0.93, 1.59) |
| - Private vehicle | 0.19 | 0.11 | 1.73 ± 0.15 (1.28, 2.34) |
| - Public transport | 0.03 | 0 | - |
| - On foot | 0 | 3×10^-3^ | - |
| Death (adjusted HR) | - | - | 0.32 ± 0.38 (0.15, 0.67) |
| SIS.NET^27^ Breast Cancer | | | |
| Physician visits | 0.60 | 0.53 | 1.13 |
| - Breast Care Centre | 0.24 | 0.23 | 1.03 |
| - Other venue | 0.36 | 0.30 | 1.20 |
| Tests/scans | 0.30 | 0.28 | 1.08 |
| - Breast cancer-related | 0.21 | 0.21 | 0.99 |
| - Non-breast cancer | 0.11 | 0.07 | 1.56 |
| Symptom reports | 0.41 | 0.18 | 2.30 |

| STAR^28^ Solid cancers | ePROM, mean (95% CI) | Control, mean (95% CI) |
| --- | --- | --- |
| Adjusted death rate | 0.024 (0.029, 0.02) | 0.031 (0.038, 0.026) |
| - computer inexperienced | 0.025 (0.034, 0.018) | 0.043 (0.062, 0.028) |
| - computer experienced | 0.023 (0.030, 0.018) | 0.028 (0.036, 0.022) |

OCC = clinic outside cancer center; HRU = health resource use; IRR = incidence rate ratio; HR = hazard ratio; CI = confidence interval; CT = computerized tomography

Supplementary Table 1: Prediction formulae coefficients for monthly rates of health resource use

| Prediction formulae coefficients for monthly rates of HRU, converted from 6 months, log-scale mean ± SE (95% CI):  $\boldsymbol{\lambda=}\frac{\boldsymbol{1}}{\boldsymbol{6}}\boldsymbol{\times}\exp\boldsymbol{(}\boldsymbol{\beta}_{\boldsymbol{0}}\boldsymbol{+}\boldsymbol{\beta}_{\boldsymbol{1}}\boldsymbol{\times ePROM+}\boldsymbol{\beta}_{\boldsymbol{2}}\boldsymbol{\times age+}\boldsymbol{\beta}_{\boldsymbol{3}}\boldsymbol{\times}{\boldsymbol{EQ}\boldsymbol{5}\boldsymbol{D}}_{\boldsymbol{b}}\boldsymbol{+}\boldsymbol{\beta}_{\boldsymbol{4}}\boldsymbol{\times CCI+}\boldsymbol{\beta}_{\boldsymbol{5}}\boldsymbol{\times NACT)}$ | | | | | |
| --- | --- | --- | --- | --- | --- |
|  |  | |  | | **Source & Cancer** |
| Coefficients | **Outpatient visits: fever** | | **Unscheduled admissions** | | Interaktor^20^ |
| Intercept $\boldsymbol{\beta}_{\boldsymbol{0}}$ | -0.33 ± 2 (-4.25, 3.59) | | 1.6 ± 1.76 (-1.85, 5.06) | | *Breast* |
| ePROM arm $\boldsymbol{\beta}_{\boldsymbol{1}}$ | -0.2 ± 0.32 (-0.84, 0.43) | | -0.22 ± 0.3 (-0.81, 0.37) | |  |
| Age $\boldsymbol{\beta}_{\boldsymbol{2}}$ | 0.01 ± 0.02 (-0.04, 0.06) | | 0.01 ± 0.02 (-0.03, 0.05) | |  |
| Base EQ5D $\boldsymbol{\beta}_{\boldsymbol{3}}$ | -1.04 ± 1.94 (-4.84, 2.76) | | -3.05 ± 1.76 (-6.5, 0.4) | |  |
| CCI $\boldsymbol{\beta}_{\boldsymbol{4}}$ | -0.13 ± 0.23 (-0.59, 0.32) | | 0.03 ± 0.19 (-0.35, 0.41) | |  |
| NACT $\boldsymbol{\beta}_{\boldsymbol{5}}$ | -0.01 ± 0.07 (-0.14, 0.12) | | -0.04 ± 0.06 (-0.16, 0.08) | |  |
|  |  | |  | |  |
| Coefficients | **Acute inpatient visits: all** | | **Acute inpatient visits: fever** | |  |
| Intercept $\boldsymbol{\beta}_{\boldsymbol{0}}$ | 0.92 ± 1.63 (-2.28, 4.11) | | -0.33 ± 2 (-4.25, 3.59) | |  |
| ePROM arm $\boldsymbol{\beta}_{\boldsymbol{1}}$ | -0.18 ± 0.26 (-0.7, 0.34) | | -0.2 ± 0.32 (-0.84, 0.43) | |  |
| Age $\boldsymbol{\beta}_{\boldsymbol{2}}$ | 0.01 ± 0.02 (-0.03, 0.04) | | 0.01 ± 0.02 (-0.04, 0.06) | |  |
| Base EQ5D $\boldsymbol{\beta}_{\boldsymbol{3}}$ | -1.29 ± 1.63 (-4.49, 1.91) | | -1.04 ± 1.94 (-4.84, 2.76) | |  |
| CCI $\boldsymbol{\beta}_{\boldsymbol{4}}$ | 0.06 ± 0.17 (-0.28, 0.39) | | -0.13 ± 0.23 (-0.59, 0.32) | |  |
| NACT $\boldsymbol{\beta}_{\boldsymbol{5}}$ | -0.07 ± 0.06 (-0.18, 0.04) | | -0.01 ± 0.07 (-0.14, 0.12) | |  |
|  |  | |  | |  |
| Coefficients | **Acute inpatient visits: GE** | | **Acute inpatient visits: UTI** | |  |
| Intercept $\boldsymbol{\beta}_{\boldsymbol{0}}$ | -4.76 ± 3.42 (-11.46, 1.95) | | -9.17 ± 6.13 (-21.18, 2.84) | |  |
| ePROM arm $\boldsymbol{\beta}_{\boldsymbol{1}}$ | 0.66 ± 0.53 (-0.38, 1.69) | | -0.09 ± 0.84 (-1.73, 1.55) | |  |
| Age $\boldsymbol{\beta}_{\boldsymbol{2}}$ | 0.02 ± 0.04 (-0.06, 0.1) | | 0.03 ± 0.05 (-0.08, 0.14) | |  |
| Base EQ5D $\boldsymbol{\beta}_{\boldsymbol{3}}$ | 1.83 ± 3.35 (-4.73, 8.4) | | 4.01 ± 6.39 (-8.52, 16.54) | |  |
| CCI $\boldsymbol{\beta}_{\boldsymbol{4}}$ | -0.31 ± 0.41 (-1.12, 0.51) | | 0.29 ± 0.44 (-0.56, 1.15) | |  |
| NACT $\boldsymbol{\beta}_{\boldsymbol{5}}$ | 0 ± 0.1 (-0.19, 0.2) | | 0.08 ± 0.14 (-0.2, 0.36) | |  |
|  |  | |  | |  |
| Coefficients | **Outpatient visits** | |  | | Interaktor^20^ |
| Intercept $\boldsymbol{\beta}_{\boldsymbol{0}}$ | 3.9 ± 1.44 (1.08, 6.72) | |  | | *Prostate* |
| ePROM arm $\boldsymbol{\beta}_{\boldsymbol{1}}$ | 0.19 ± 0.17 (-0.15, 0.53) | |  | |  |
| Age $\boldsymbol{\beta}_{\boldsymbol{2}}$ | 0.02 ± 0.02 (-0.01, 0.05) | |  | |  |
| Base EQ5D $\boldsymbol{\beta}_{\boldsymbol{3}}$ | -3.44 ± 1.14 (-5.66, -1.21) | |  | |  |
| CCI $\boldsymbol{\beta}_{\boldsymbol{4}}$ | 0.01 ± 0.07 (-0.13, 0.15) | |  | |  |
|  |  |  |  |  |  |

CI = confidence interval; SE = standard error; EQ5D_b_ = baseline EQ5D health utility score; CCI = Charlson morbidity index; NACT = neoadjuvant chemotherapy cycles; GE = gastroenteritis

## Phoneme/Interactor Multivariate Regression Details Sent by Authors

The tables in this section are copied directly from the document sent by the authors.

| Table 4: Parameter Estimates Acute outpatient visits for fever | | | | | | | | | | |
| --- | --- | --- | --- | --- | --- | --- | --- | --- | --- | --- |
| Parameter | B | Std. Error | 95% Wald Confidence Interval | | Hypothesis Test | | | Exp(B) | 95% Wald Confidence Interval for Exp(B) | |
|  |  |  | Lower | Upper | Wald Chi-Square | df | Sig. |  | Lower | Upper |
| (Intercept) | -,332 | 2,0015 | -4,254 | 3,591 | ,027 | 1 | ,868 | ,718 | ,014 | 36,278 |
| [Grupp=0] | -,204 | ,3228 | -,837 | ,429 | ,399 | 1 | ,527 | ,815 | ,433 | 1,535 |
| [Grupp=1] | 0^a^ | . | . | . | . | . | . | 1 | . | . |
| Age at inclusion | ,010 | ,0233 | -,035 | ,056 | ,192 | 1 | ,662 | 1,010 | ,965 | 1,057 |
| EQ5DP_baseline | -1,041 | 1,9377 | -4,839 | 2,757 | ,289 | 1 | ,591 | ,353 | ,008 | 15,750 |
| Charlson comorbidity score | -,132 | ,2311 | -,585 | ,321 | ,327 | 1 | ,567 | ,876 | ,557 | 1,378 |
| antal kurer NACT | -,012 | ,0659 | -,141 | ,117 | ,033 | 1 | ,856 | ,988 | ,868 | 1,124 |
| (Scale) | 1^b^ |  |  |  |  |  |  |  |  |  |
| (Negative binomial) | 1^b^ |  |  |  |  |  |  |  |  |  |
| Dependent Variable: Acute inpatient visits fever  Model: (Intercept), Grupp, Age at inclusion, EQ5DP_baseline, Charlson comorbidity score, antal kurer NACT | | | | | | | | | | |
| a. Set to zero because this parameter is redundant. | | | | | | | | | | |
| b. Fixed at the displayed value. | | | | | | | | | | |

| Table 5: Parameter Estimates Unplanned admission to inpatient care | | | | | | | | | | |
| --- | --- | --- | --- | --- | --- | --- | --- | --- | --- | --- |
| Parameter | B | Std. Error | 95% Wald Confidence Interval | | Hypothesis Test | | | Exp(B) | 95% Wald Confidence Interval for Exp(B) | |
|  |  |  | Lower | Upper | Wald Chi-Square | df | Sig. |  | Lower | Upper |
| (Intercept) | 1,604 | 1,7630 | -1,852 | 5,059 | ,827 | 1 | ,363 | 4,970 | ,157 | 157,429 |
| [Grupp=0] | -,220 | ,3019 | -,811 | ,372 | ,530 | 1 | ,467 | ,803 | ,444 | 1,451 |
| [Grupp=1] | 0^a^ | . | . | . | . | . | . | 1 | . | . |
| Age at inclusion | ,010 | ,0213 | -,031 | ,052 | ,239 | 1 | ,625 | 1,010 | ,969 | 1,054 |
| EQ5DP_baseline | -3,048 | 1,7599 | -6,497 | ,401 | 2,999 | 1 | ,083 | ,047 | ,002 | 1,494 |
| Charlson comorbidity score | ,029 | ,1934 | -,350 | ,408 | ,023 | 1 | ,880 | 1,030 | ,705 | 1,504 |
| antal kurer NACT | -,036 | ,0606 | -,155 | ,083 | ,353 | 1 | ,553 | ,965 | ,857 | 1,086 |
| (Scale) | 1^b^ |  |  |  |  |  |  |  |  |  |
| (Negative binomial) | 1^b^ |  |  |  |  |  |  |  |  |  |
| Dependent Variable: oplanerad inskrivning till slutenvård, antal  Model: (Intercept), Grupp, Age at inclusion, EQ5DP_baseline, Charlson comorbidity score, antal kurer NACT | | | | | | | | | | |
| a. Set to zero because this parameter is redundant. | | | | | | | | | | |
| b. Fixed at the displayed value. | | | | | | | | | | |

| Table 6: Parameter Estimates Acute inpatient visits all | | | | | | | | | | |
| --- | --- | --- | --- | --- | --- | --- | --- | --- | --- | --- |
| Parameter | B | Std. Error | 95% Wald Confidence Interval | | Hypothesis Test | | | Exp(B) | 95% Wald Confidence Interval for Exp(B) | |
|  |  |  | Lower | Upper | Wald Chi-Square | df | Sig. |  | Lower | Upper |
| (Intercept) | ,915 | 1,6288 | -2,278 | 4,107 | ,316 | 1 | ,574 | 2,497 | ,103 | 60,789 |
| [Grupp=0] | -,182 | ,2636 | -,698 | ,335 | ,474 | 1 | ,491 | ,834 | ,497 | 1,398 |
| [Grupp=1] | 0^a^ | . | . | . | . | . | . | 1 | . | . |
| Age at inclusion | ,006 | ,0187 | -,031 | ,042 | ,094 | 1 | ,759 | 1,006 | ,969 | 1,043 |
| EQ5DP_baseline | -1,291 | 1,6340 | -4,493 | 1,912 | ,624 | 1 | ,430 | ,275 | ,011 | 6,768 |
| Charlson comorbidity score | ,055 | ,1702 | -,278 | ,389 | ,106 | 1 | ,745 | 1,057 | ,757 | 1,476 |
| antal kurer NACT | -,068 | ,0568 | -,179 | ,043 | 1,430 | 1 | ,232 | ,934 | ,836 | 1,044 |
| (Scale) | 1^b^ |  |  |  |  |  |  |  |  |  |
| (Negative binomial) | 1^b^ |  |  |  |  |  |  |  |  |  |
| Dependent Variable: Acute inpatient visits  Model: (Intercept), Grupp, Age at inclusion, EQ5DP_baseline, Charlson comorbidity score, antal kurer NACT | | | | | | | | | | |
| a. Set to zero because this parameter is redundant. | | | | | | | | | | |
| b. Fixed at the displayed value. | | | | | | | | | | |

| Table 7: Parameter Estimates Acute inpatient visits for fever | | | | | | | | | | |
| --- | --- | --- | --- | --- | --- | --- | --- | --- | --- | --- |
| Parameter | B | Std. Error | 95% Wald Confidence Interval | | Hypothesis Test | | | Exp(B) | 95% Wald Confidence Interval for Exp(B) | |
|  |  |  | Lower | Upper | Wald Chi-Square | df | Sig. |  | Lower | Upper |
| (Intercept) | -,332 | 2,0015 | -4,254 | 3,591 | ,027 | 1 | ,868 | ,718 | ,014 | 36,278 |
| [Grupp=0] | -,204 | ,3228 | -,837 | ,429 | ,399 | 1 | ,527 | ,815 | ,433 | 1,535 |
| [Grupp=1] | 0^a^ | . | . | . | . | . | . | 1 | . | . |
| Age at inclusion | ,010 | ,0233 | -,035 | ,056 | ,192 | 1 | ,662 | 1,010 | ,965 | 1,057 |
| EQ5DP_baseline | -1,041 | 1,9377 | -4,839 | 2,757 | ,289 | 1 | ,591 | ,353 | ,008 | 15,750 |
| Charlson comorbidity score | -,132 | ,2311 | -,585 | ,321 | ,327 | 1 | ,567 | ,876 | ,557 | 1,378 |
| antal kurer NACT | -,012 | ,0659 | -,141 | ,117 | ,033 | 1 | ,856 | ,988 | ,868 | 1,124 |
| (Scale) | 1^b^ |  |  |  |  |  |  |  |  |  |
| (Negative binomial) | 1^b^ |  |  |  |  |  |  |  |  |  |
| Dependent Variable: Acute inpatient visits fever  Model: (Intercept), Grupp, Age at inclusion, EQ5DP_baseline, Charlson comorbidity score, antal kurer NACT | | | | | | | | | | |
| a. Set to zero because this parameter is redundant. | | | | | | | | | | |
| b. Fixed at the displayed value. | | | | | | | | | | |

| Table 8: Parameter Estimates Acute inpatient visits for gastroenteritis | | | | | | | | | | |
| --- | --- | --- | --- | --- | --- | --- | --- | --- | --- | --- |
| Parameter | B | Std. Error | 95% Wald Confidence Interval | | Hypothesis Test | | | Exp(B) | 95% Wald Confidence Interval for Exp(B) | |
|  |  |  | Lower | Upper | Wald Chi-Square | df | Sig. |  | Lower | Upper |
| (Intercept) | -4,756 | 3,4215 | -11,462 | 1,950 | 1,932 | 1 | ,165 | ,009 | 1,053×10^−5 | 7,031 |
| [Grupp=0] | ,658 | ,5284 | -,378 | 1,694 | 1,551 | 1 | ,213 | 1,931 | ,686 | 5,440 |
| [Grupp=1] | 0^a^ | . | . | . | . | . | . | 1 | . | . |
| Age at inclusion | ,019 | ,0391 | -,058 | ,095 | ,229 | 1 | ,633 | 1,019 | ,944 | 1,100 |
| EQ5DP_baseline | 1,832 | 3,3484 | -4,731 | 8,395 | ,299 | 1 | ,584 | 6,245 | ,009 | 4422,840 |
| Charlson comorbidity score | -,305 | ,4133 | -1,115 | ,505 | ,545 | 1 | ,460 | ,737 | ,328 | 1,657 |
| antal kurer NACT | ,004 | ,0995 | -,192 | ,199 | ,001 | 1 | ,972 | 1,004 | ,826 | 1,220 |
| (Scale) | 1^b^ |  |  |  |  |  |  |  |  |  |
| (Negative binomial) | 1^b^ |  |  |  |  |  |  |  |  |  |
| Dependent Variable: Acute inpatient visits gastroenteritis  Model: (Intercept), Grupp, Age at inclusion, EQ5DP_baseline, Charlson comorbidity score, antal kurer NACT | | | | | | | | | | |
| a. Set to zero because this parameter is redundant. | | | | | | | | | | |
| b. Fixed at the displayed value. | | | | | | | | | | |

| Table 9: Parameter Estimates Acute inpatient visits for anaemia | | | | | | | | | | |
| --- | --- | --- | --- | --- | --- | --- | --- | --- | --- | --- |
| Parameter | B | Std. Error | 95% Wald Confidence Interval | | Hypothesis Test | | | Exp(B) | 95% Wald Confidence Interval for Exp(B) | |
|  |  |  | Lower | Upper | Wald Chi-Square | df | Sig. |  | Lower | Upper |
| (Intercept) | -8,661 | 5,055 | -18,568 | 1,246 | 2,936 | 1 | ,087 | <,001 | 8,633×10^−9 | 3,477 |
| [Grupp=0] | -,10 | ,74 | -1,561 | 1,351 | ,020 | 1 | ,89 | ,901 | ,210 | 3,863 |
| [Grupp=1] | 0^a^ | . | . | . | . | . | . | 1,0 | . | . |
| Age at inclusion | ,10 | ,07 | -,038 | ,229 | 1,984 | 1 | ,16 | 1,101 | ,963 | 1,258 |
| EQ5DP_baseline | 1,85 | 4,74 | -7,442 | 11,138 | ,152 | 1 | ,70 | 6,346 | ,001 | 68734,141 |
| Charlson comorbidity score | -,94 | ,73 | -2,377 | ,491 | 1,662 | 1 | ,20 | ,389 | ,093 | 1,633 |
| antal kurer NACT | ,03 | ,14 | -,248 | ,305 | ,041 | 1 | ,84 | 1,029 | ,780 | 1,357 |
| (Scale) | 1^b^ |  |  |  |  |  |  |  |  |  |
| (Negative binomial) | 1^b^ |  |  |  |  |  |  |  |  |  |
| Dependent Variable: Acute inpatient visits anaemia  Model: (Intercept), Grupp, Age at inclusion, EQ5DP_baseline, Charlson comorbidity score, antal kurer NACT | | | | | | | | | | |
| a. Set to zero because this parameter is redundant. | | | | | | | | | | |
| b. Fixed at the displayed value. | | | | | | | | | | |

| Table 10: Parameter Estimates acute inpatient visits for urinary tract infection | | | | | | | | | | |
| --- | --- | --- | --- | --- | --- | --- | --- | --- | --- | --- |
| Parameter | B | Std. Error | 95% Wald Confidence Interval | | Hypothesis Test | | | Exp(B) | 95% Wald Confidence Interval for Exp(B) | |
|  |  |  | Lower | Upper | Wald Chi-Square | df | Sig. |  | Lower | Upper |
| (Intercept) | -9,173 | 6,1270 | -21,181 | 2,836 | 2,241 | 1 | ,134 | <,001 | 6,325×10^−10 | 17,046 |
| [Grupp=0] | -,093 | ,8371 | -1,733 | 1,548 | ,012 | 1 | ,912 | ,911 | ,177 | 4,702 |
| [Grupp=1] | 0^a^ | . | . | . | . | . | . | 1 | . | . |
| Age at inclusion | ,030 | ,0544 | -,077 | ,136 | ,299 | 1 | ,584 | 1,030 | ,926 | 1,146 |
| EQ5DP_baseline | 4,009 | 6,3934 | -8,522 | 16,540 | ,393 | 1 | ,531 | 55,108 | ,000 | 15250488 |
| Charlson comorbidity score | ,293 | ,4351 | -,560 | 1,145 | ,452 | 1 | ,501 | 1,340 | ,571 | 3,144 |
| antal kurer NACT | ,077 | ,1416 | -,200 | ,355 | ,297 | 1 | ,586 | 1,080 | ,818 | 1,426 |
| (Scale) | 1^b^ |  |  |  |  |  |  |  |  |  |
| (Negative binomial) | 1^b^ |  |  |  |  |  |  |  |  |  |
| Dependent Variable: Acute inpatient visits urinary tract infection  Model: (Intercept), Grupp, Age at inclusion, EQ5DP_baseline, Charlson comorbidity score, antal kurer NACT | | | | | | | | | | |
| a. Set to zero because this parameter is redundant. | | | | | | | | | | |
| b. Fixed at the displayed value. | | | | | | | | | | |

| Table 11: Parameter Estimates Outpatient care visits (all) | | | | | | | | | | |
| --- | --- | --- | --- | --- | --- | --- | --- | --- | --- | --- |
| Parameter | B | Std. Error | 95% Wald Confidence Interval | | Hypothesis Test | | | Exp(B) | 95% Wald Confidence Interval for Exp(B) | |
|  |  |  | Lower | Upper | Wald Chi-Square | df | Sig. |  | Lower | Upper |
| (Intercept) | 3,900 | 1,4406 | 1,077 | 6,724 | 7,330 | 1 | ,007 | 49,418 | 2,935 | 832,048 |
| [Group=0] | ,190 | ,1719 | -,147 | ,527 | 1,225 | 1 | ,268 | 1,210 | ,864 | 1,694 |
| [Group=1] | 0^a^ | . | . | . | . | . | . | 1 | . | . |
| age | ,019 | ,0155 | -,011 | ,049 | 1,493 | 1 | ,222 | 1,019 | ,989 | 1,051 |
| EQ5D(P) baseline | -3,439 | 1,1351 | -5,664 | -1,214 | 9,179 | 1 | ,002 | ,032 | ,003 | ,297 |
| Charlson comorbidity score | ,007 | ,0718 | -,134 | ,148 | ,010 | 1 | ,922 | 1,007 | ,875 | 1,159 |
| (Scale) | 1^b^ |  |  |  |  |  |  |  |  |  |
| (Negative binomial) | 1^b^ |  |  |  |  |  |  |  |  |  |
| Dependent Variable: OVR_n_besök_ALLT  Model: (Intercept), Group, age, EQ5D(P) baseline, Charlson comorbidity score | | | | | | | | | | |
| a. Set to zero because this parameter is redundant. | | | | | | | | | | |
| b. Fixed at the displayed value. | | | | | | | | | | |

| Table 12: Parameter Estimates Patients with an acute outpatient visit yes/no | | | | | | | | | | |
| --- | --- | --- | --- | --- | --- | --- | --- | --- | --- | --- |
| Parameter | B | Std. Error | 95% Wald Confidence Interval | | Hypothesis Test | | | Exp(B) | 95% Wald Confidence Interval for Exp(B) | |
|  |  |  | Lower | Upper | Wald Chi-Square | df | Sig. |  | Lower | Upper |
| (Intercept) | -,841 | 2,8410 | -6,409 | 4,727 | ,088 | 1 | ,767 | ,431 | ,002 | 113,006 |
| [Group=0] | -,017 | ,3502 | -,703 | ,670 | ,002 | 1 | ,962 | ,983 | ,495 | 1,953 |
| [Group=1] | 0^a^ | . | . | . | . | . | . | 1 | . | . |
| age | ,017 | ,0300 | -,041 | ,076 | ,333 | 1 | ,564 | 1,017 | ,959 | 1,079 |
| EQ5D(P) baseline | ,078 | 2,3702 | -4,568 | 4,724 | ,001 | 1 | ,974 | 1,081 | ,010 | 112,574 |
| Charlson comorbidity score | ,078 | ,1496 | -,215 | ,371 | ,274 | 1 | ,601 | 1,081 | ,807 | 1,450 |
| (Scale) | 1^b^ |  |  |  |  |  |  |  |  |  |
| Dependent Variable: OVR_AKUT_JA_NEJ  Model: (Intercept), Group, age, EQ5D(P) baseline, Charlson comorbidity score | | | | | | | | | | |
| a. Set to zero because this parameter is redundant. | | | | | | | | | | |
| b. Fixed at the displayed value. | | | | | | | | | | |

| Table 13: Parameter Estimates Patients with acute visit for urological problem | | | | | | | | | | |
| --- | --- | --- | --- | --- | --- | --- | --- | --- | --- | --- |
| Parameter | B | Std. Error | 95% Wald Confidence Interval | | Hypothesis Test | | | Exp(B) | 95% Wald Confidence Interval for Exp(B) | |
|  |  |  | Lower | Upper | Wald Chi-Square | df | Sig. |  | Lower | Upper |
| (Intercept) | -3,647 | 4,8077 | -13,070 | 5,776 | ,576 | 1 | ,448 | ,026 | 2,107×10^−6 | 322,325 |
| [Group=0] | -,127 | ,6197 | -1,341 | 1,088 | ,042 | 1 | ,838 | ,881 | ,261 | 2,968 |
| [Group=1] | 0^a^ | . | . | . | . | . | . | 1 | . | . |
| age | -,022 | ,0568 | -,134 | ,089 | ,156 | 1 | ,693 | ,978 | ,875 | 1,093 |
| EQ5D(P) baseline | 8,368 | 3,5323 | 1,444 | 15,291 | 5,611 | 1 | ,018 | 4304,883 | 4,239 | 4371829,666 |
| Charlson comorbidity score | ,142 | ,2835 | -,414 | ,697 | ,249 | 1 | ,618 | 1,152 | ,661 | 2,008 |
| (Scale) | 1^b^ |  |  |  |  |  |  |  |  |  |
| Dependent Variable: OVR_AKUT_URO_JA_NEJ  Model: (Intercept), Group, age, EQ5D(P) baseline, Charlson comorbidity score | | | | | | | | | | |
| a. Set to zero because this parameter is redundant. | | | | | | | | | | |
| b. Fixed at the displayed value. | | | | | | | | | | |

| Table 14: Variables in the Equation Patients with an unplanned admission to inpatient care | | | | | | | | | |
| --- | --- | --- | --- | --- | --- | --- | --- | --- | --- |
|  | | B | S.E. | Wald | df | Sig. | Exp(B) | 95% C.I.for EXP(B) | |
|  |  |  |  |  |  |  |  | Lower | Upper |
| Step 1^a^ | Group(1) | -,081 | ,593 | ,019 | 1 | ,892 | ,922 | ,288 | 2,951 |
|  | Age at inclusion | ,070 | ,060 | 1,351 | 1 | ,245 | 1,072 | ,953 | 1,206 |
|  | EQ5DP_baseline | -5,133 | 3,480 | 2,175 | 1 | ,140 | ,006 | ,000 | 5,410 |
|  | Charlson comorbidity score | -,032 | ,253 | ,016 | 1 | ,900 | ,969 | ,590 | 1,590 |
|  | Constant | -2,662 | 4,934 | ,291 | 1 | ,590 | ,070 |  |  |
| a. Variable(s) entered on step 1: Group, Age at inclusion, EQ5DP_baseline, Charlson comorbidity score. | | | | | | | | | |

| Table 15: Parameter Estimates Patients with an inpatient visit yes/no | | | | | | | | | | |
| --- | --- | --- | --- | --- | --- | --- | --- | --- | --- | --- |
| Parameter | B | Std. Error | 95% Wald Confidence Interval | | Hypothesis Test | | | Exp(B) | 95% Wald Confidence Interval for Exp(B) | |
|  |  |  | Lower | Upper | Wald Chi-Square | df | Sig. |  | Lower | Upper |
| (Intercept) | 1,403 | 4,0237 | -6,483 | 9,290 | ,122 | 1 | ,727 | 4,069 | ,002 | 10826,391 |
| [Group=0] | ,027 | ,4866 | -,927 | ,980 | ,003 | 1 | ,957 | 1,027 | ,396 | 2,665 |
| [Group=1] | 0^a^ | . | . | . | . | . | . | 1 | . | . |
| age | -,065 | ,0486 | -,161 | ,030 | 1,805 | 1 | ,179 | ,937 | ,852 | 1,030 |
| EQ5D(P) baseline | 6,017 | 2,9175 | ,299 | 11,735 | 4,254 | 1 | ,039 | 410,398 | 1,349 | 124898,645 |
| Charlson comorbidity score | -,064 | ,1977 | -,451 | ,324 | ,104 | 1 | ,747 | ,938 | ,637 | 1,382 |
| (Scale) | 1^b^ |  |  |  |  |  |  |  |  |  |
| Dependent Variable: SLV_ALLT_JA_NEJ  Model: (Intercept), Group, age, EQ5D(P) baseline, Charlson comorbidity score | | | | | | | | | | |
| a. Set to zero because this parameter is redundant. | | | | | | | | | | |
| b. Fixed at the displayed value. | | | | | | | | | | |

## R code used to produce secondary analysis of PHONEME/Interactor results

```
# SETUP ------------------------------------------------------------------------
library(tidyr)
library(dplyr)
library(stringr)
library(scales)
library(arrayhelpers)

set.seed(123)
options(scipen = 999)

# LOAD DATA --------------------------------------------------------------------

coef_df <- read.csv("R/coef.csv")
n_v <- 1:length(rownames(coef_df))

coef_v <- unique(coef_df$coef)
coef_n <- length(coef_v)
events_v <- unique(coef_df$event)
ev_n <- length(events_v)

# SIM PARAMETERS ---------------------------------------------------------------
psa_n <- 1000
psa_v <- paste("psa", 1:psa_n, sep = "_")
psall_v <- c("mu", psa_v)
psall_n <- psa_n + 1

psim_v <- c("incpt" = 1, "arm" = 1, "age" = 60, "u" = NA, "cci" = 1, "nact" = 2)
u_v <- 0:20/20 # test baseline utility
u_n <- 21
ul_v <- paste("U", u_v * 100, sep ="_")

interv_n <- 4
interv_v <- paste(round(1:interv_n / interv_n * 100), "%", sep = "")

# remove non eq5d variables from psa [option]
# coef_df[coef_df$coef != "eq5d", "se"] <- 0


# SAMPLE -----------------------------------------------------------------------

coef_a <- array(
  NA, dim = c(coef_n, 2, ev_n), dimnames = list(
    "Coefficient" = coef_v, "Est" = c("mu", "se"), "Event" = events_v
  )
)

sigma_a <- array(
  NA, dim = c(6, 6, ev_n), dimnames = list(1:6, 1:6, "Event" = events_v)
)

psa_a <- array(
  NA, dim = c(coef_n, psall_n, ev_n), dimnames = list(
    "Coef" = coef_v, "Run" = psall_v, "Event" = events_v)
) # psa_a[,1:10,]


rate_a <- array(
  NA, dim = c(u_n, psall_n, ev_n), dimnames = list(
    "U" = ul_v, "Run" = psall_v, "Event" = events_v
  ) # rate_a[,1:10,]
)

rategr_a <- array(
  NA, dim = c(u_n, 2, interv_n, ev_n), dimnames = list(
    "U" = ul_v, "Bound" = c("l", "u"),
    "Interval" = interv_v, "Event" = events_v)
)

for(e in events_v){
  coef_a[,,e] <- filter(coef_df, event == e)[c("mu", "se")] %>% as.matrix
  sigma_a[,,e] <- diag(coef_a[,"se",e]^2)
  psa_a[,psa_v,e] <- MASS::mvrnorm(
    psa_n, mu = coef_a[,"mu",e], Sigma = sigma_a[,,e]) %>% t
}

psa_a[,"mu",] <- coef_a[,"mu",]

for(u in 1:u_n){
  psim_v["u"] <- u_v[u]
  rate_a[ul_v[u], ,] <- 0.2558 * exp(apply(psa_a * psim_v, 2:3, sum))
}

for(e in events_v){
  for(p in 1:interv_n){
    dev_n <- p * 1/interv_n/2
    qtl_v <- c(0.5 + c(-1, 1) * dev_n)

    rategr_a[,,p,e] <- rate_a[,psa_v,e] %>%
      apply(1, function(i) quantile(i, qtl_v)) %>% t

  }
} # rategr_a


# PLOTS ------------------------------------------------------------------------

blue_v <- c("#e8eef1", "#43b0f1", "#057dcd", "#1e3d58")

par(mfrow = c(3, 2), oma = c(4, 6, 2, 2), mar = c(0, 0.5, 1.5, 0.5))
xtick_v <- 0:10/10 * 5
ytick_v <- 0:4/4

for(e in events_v){
  matplot(
    y = u_v, x = rate_a[, "mu", e],  main = e,
    xlab = NA, ylab = NA, ylim = 0:1,  xaxs = "i", yaxs = "i",
    xlim = c(0, 1.5 * max(rate_a[, "mu",])), xaxt = 'n', yaxt = 'n',
    panel.first = {
      for(p in interv_n:1){
        polygon(
          x = c(rategr_a[,"l",interv_v[p], e],
                rev(rategr_a[,"u",interv_v[p], e])),
          y = c(u_v, rev(u_v)), lty = 0,
          col = blue_v[2] %>% alpha(0.2)
        )
      }
      abline(h = ytick_v, v = xtick_v, col = "white", lty = 2)
    },
    type = "l", lty = 1, lwd = 3, col = blue_v[4] #  %>% alpha(0.1)
  )

  if(e %in% events_v[c(1, 3, 5)]) axis(
    2, at = ytick_v, labels = ytick_v, las = 1)
  if(e %in% events_v[5:6]) axis(1, at = xtick_v, labels = xtick_v)
  if(e=="unplanned") {
    text(
      x = 2.5, y = c(0.1, 0.3, 0.6, 0.95), interv_v, col = "#057dcd", cex = 1.5)
    text(x=1, y=0.25, "deterministic estimate", adj = 0)
    text(x=2.25, y=0.5, "probabilistic estimates (share)", col = "#057dcd", srt = 90)
  }
}

mtext(outer = T, side = 1, line = 2.5,
      "Rate of Admissions by Type per Person-Month")
mtext(outer = T, side = 2, line = 2.5, "Baseline EQ-5D Health Utility")
```

## Risk of Bias and Reporting Standards Checklists

Checklists were assigned to the included publications based on the title and primary objective; for example, standalone HEA papers were evaluated using the CHEERS 2022 checklist, while reports of RCTs containing a secondary within-trial HEA or HRU section were assessed using the Cochrane Risk of Bias (RoB) 2 checklist.^38,39^ Non-randomized clinical trials (NRCTs) and observational studies were evaluated through the “Risk Of Bias In Non-randomized Studies of Interventions” (ROBINS-I) assessment tool.^40^ Implementation studies were grouped into three categories: (1) two studies using predominantly quantitative methods were evaluated using ROBINS-I, (2) two studies were mainly qualitative evaluations “concerned with the development and implementation of social policy, programmes and practice” and therefore evaluated using the UK Cabinet Office’s dedicated appraisal tool, and (3) one study was evaluated using the Mixed Methods Appraisal Tool.^40–43^ The scoping review by Aapro et al. (2020) was evaluated with the Preferred Reporting Items for Systematic reviews and Meta-Analyses checklist for scoping reviews (PRISMA-ScR).^1,44^

For RCTs, reports and protocols relating to the same analysis were grouped for RoB 2 assessment.

Health economic reporting in this review generally followed CHEERS, with points deducted mainly for lack of patient/participant engagement and the absence of a dedicated health economic analysis plan (separate from the study protocol).

The risk of bias assessments of the included RCT reports and protocols suggests a mixed quality of evidence. However, this was mostly due to aspects of the complex intervention process inherent in ePROM interventions; such interventions are impossible to blind and conceal from either participants or delivering HCPs. While Smits et al. (2025) cite evidence of a minimal selection bias effect in studies of cancer survivors aware of randomization, the key risks stem from contamination bias and diffusion, as discussed (respectively) in the eRAPID and PRISMS reports.^5,11,21^ In the case of eRAPID, the authors discuss in detail the limitations stemming from a single HCP group providing care to patients in both trial arms and identified two problems: (1) HCPs may have been sensitized by ePROM reports and initiated more in-depth symptom screening in the control arm (i.e., contamination), and (2) HCPs treating mostly control arm patients were less effectively engaged in performing their role within the ePROM intervention system; these biases are likely to attenuate evidence of ePROM benefits.^11,12^ Outright contamination bias was avoided in the PRISMS intervention through separate trial-arm allocation of HCPs; furthermore, the authors prepared for and showed evidence of preventing a diffusion effect, whereby HCPs from the intervention arm influence usual care by sharing insight with control-arm colleagues.^21^

RCTs which did not discuss or evaluate contamination and diffusion risk were downgraded in their risk of bias judgements, consistent with Section 2 of the RoB 2 appraisal algorithm (deviations due to trial context).^38^ The SYMPRO-Lung study was judged to avoid this risk thanks to its stepped-wedge design, which is more tailored to complex intervention implementation contexts.^30^ Similarly, the CAPRI trial achieved a low risk of contamination bias through designating “nurse navigators” to deliver the ePROM intervention without contact with control arm patients, although diffusion may still have been possible. RCTs reporting on strictly reactive systems without regular ePROM monitoring and management by HCPs (e.g., YES) were not judged to be at a significant risk of contamination or diffusion biases. Low-risk judgements did not apply to reports of reactive interventions that failed to clarify HCPs’ roles in delivering the interventions (e.g., PRO-NET).

The contamination and diffusion risks were lower in the included NRCT studies, each for different reasons: (1) Ferre et al. (2021) presented a descriptive-only cohort study without a control arm to contaminate, (2) ICECARE-P separated centers through cluster-randomized trial design, (3) PROMPT-CARE’s low enrolment led to its intervention arm (n = 328) being dwarfed by matched controls (n = 1,312) and eligible patients (n = 3,699), reducing but not removing a potential effect, and (4) the Programme trial employed a historical control.^15,16,23,31^

The included implementation studies were also characterized by mixed reliability and bias risk. The Affordable Better study was judged to present a serious risk of bias, especially its cost-consequence analysis component; this is mainly due to (1) not controlling for any potentially confounding variables and (2) simulating the standard care group with a hypothetical counterfactual without specifying or justifying the assumptions used to do so. AmbuFlex was judged to be at low risk of bias, due to its reporting of descriptive-only statistics specific to its ePROM system’s implementation (e.g., numbers and proportions of automated alerts by severity grade). The Kaiku Health mixed-methods pilot study suffered less from risk of bias but more from inadequate data collection for its quantitative component, i.e., time-motion data collected through quantitative-only end-of-study recall surveys, which is an important contributor to the study's conclusions.^17^ However, as a pilot study, it may prove helpful in planning more rigorous mixed-methods time motion research, which could be very important to health economic modelling of ePROM interventions.^45^

No shortcomings in any of the Cabinet Office appraisal domains were identified in the MyPal and VBHC Initiative implementation papers; both included clear and well-presented findings, sound study design and sample composition, coherent and consistent completed or proposed analyses and reporting, and thorough discussion of assumptions, ethical considerations, and auditability.

For checklists, the “✓” icon represents the number of items successfully addressed as a fraction of the total. Conversely, the “X” shows the fraction of items the authors failed to address. The “N/A” column shows the fraction of checklist sections not applicable to each publication. The risk grade shows a singular composite final judgment for tools guided by algorithms, where the positive and negative implications of individual yes/no answers differ depending on the component question (e.g., RoB 2).^38^

Table 16: Risk of bias and reporting standards checklists

| Study | Publication | Checklist | Type | Score/Grade | | |
| --- | --- | --- | --- | --- | --- | --- |
|  |  |  |  | ✓ | X | N/A |
| N/A | Aapro et al. (2020)^1^ | PRISMA-ScR^44^ | Reporting quality | 19/22 | 2/22 | 1/22 |
| N/A | Ferre et al. (2021)^15^ | ROBINS-I^40^ | Risk of Bias | Low risk | | |
| Affordable Better | Qaderi et al. (2021)^2^ | ROBINS-I^40^ | Risk of Bias | Serious risk | | |
| AmbuFlex | Schougaard et al. (2016)^3^ | ROBINS-I^40^ | Risk of Bias | Serious risk | | |
| APCOT | Sprave et al. (2020)^4^ | RoB 2^38^ | Risk of Bias | Some concerns | | |
| CAG | Smits et al. (2025)^5^ | RoB 2^38^ | Risk of Bias | Some concerns | | |
| CAPRI | Mir et al. (2022)^6^ | RoB 2^38^ | Risk of Bias | Low risk | | |
|  | Minvielle et al. (2024)^7^ | CHEERS^39^ | Reporting quality | 19/28 | 5/28 | 4/28 |
| eHealth@Hospital-2-Home | Storm et al. (2024)^8^ | RoB 2^38^ | Risk of Bias | Low risk | | |
| eRAPID | Absolom et al. (2017)^9^ | RoB 2^38^ | Risk of Bias | High risk | | |
|  | Velikova et al. (2022)^12^ |  | Risk of Bias |  |  |  |
|  | Absolom et al. (2021)^11^ |  | Risk of Bias |  |  |  |
|  | Holch et al. (2018)^10^ | RoB 2^38^ | Risk of Bias | Some concerns | | |
|  | Dawkins et al. (2024)^13^ | CHEERS^39^ | Reporting quality | 24/28 | 3/28 | 1/28 |
| eSMART | Maguire et al. (2018)^46^ | RoB 2^38^ | Risk of Bias | Some concerns | | |
| ICARE-P | Nanton et al. (2017)^16^ | ROBINS-I^40^ | Risk of Bias | Moderate risk | | |
| Kaiku Health | Schmalz et al. (2020)^17^ | MMAT^43^ | Reporting quality | 14/25 | 2/25 | 11/25 |
| LYSA | Kearns et al. (2022)^18^ | RoB 2^38^ | Risk of Bias | Some concerns | | |
| MyPal | Koumakis et al. (2021)^19^ | Cabinet Office^42^ | Reporting quality | N/A | | |
| Oncokompas | van der Hout et al. (2020)^47^ | CHEERS^39^ | Reporting quality | 22/28 | 5/28 | 1/28 |
| PhONEME/Interaktor | Crafoord et al. (2025)^20^ | CHEERS^39^ | Reporting quality | 23/28 | 4/28 | 1/28 |
| PRISMS | Breen et al. (2015)^21^ | RoB 2^38^ | Risk of Bias | Low risk | | |
| PROMISE | Webb et al. (2024)^22^ | RoB 2^38^ | Risk of Bias | Some concerns | | |
| PROMPT-Care | Girgis et al. (2020)^23^ | ROBINS-I^40^ | Risk of Bias | Moderate risk | | |
| PRO-NET | Xia et al. (2025)^24^ | RoB 2^38^ | Risk of Bias | High risk | | |
| Sentinel | Denis et al. (2017)^48^ | RoB 2^38^ | Risk of Bias | High risk | | |
|  | Lizée et al. (2019)^26^ | CHEERS^39^ | Reporting quality | 22/28 | 5/28 | 1/28 |
| SIS.NET | Wheelock et al. (2014)^27^ | RoB 2^38^ | Risk of Bias | High risk | | |
| STAR | Basch et al. (2016)^49^ | RoB 2^38^ | Risk of Bias | High risk | | |
|  | Nixon et al. (2018)^29^ | CHEERS^39^ | Reporting quality | 21/28 | 6/28 | 1/28 |
| SYMPRO-Lung | Billingy et al. (2021)^30^ | RoB 2^38^ | Risk of Bias | Low risk | | |
| The Programme | Frankland et al. (2019)^31^ | ROBINS-I^40^ | Risk of Bias | Low | | |
| VBHC-initiative | van Egdom et al. (2019)^32^ | Cabinet Office^42^ | Reporting quality | N/A | | |
| YES | Dibble et al. (2025)^33^ | RoB 2^38^ | Risk of Bias | Low risk | | |

RoB 2 = Cochrane Risk of Bias 2, CHEERS = Consolidated Heal

| Table 17: Consolidated Health Economic Evaluation Reporting Standards 2022 Checklist | | | Nixon et al. (2018) | Minvielle et al. (2024) | Dawkins et al. (2024) | van der Hout et al. (2020) | Crafoord et al. (2025) | Lizée et al. (2019) |
| --- | --- | --- | --- | --- | --- | --- | --- | --- |
| Title | 1 | Identify the study as an economic evaluation and specify the interventions being compared. | ✓ | ✓ | ✓ | ✓ | ✓ | ✓ |
| Abstract | 2 | Provide a structured summary that highlights context, key methods, results, and alternative analyses. | ✓ | ✓ | ✓ | ✓ | ✓ | ✓ |
| Introduction: Background and objectives | 3 | Give the context for the study, the study question, and its practical relevance for decision making in policy or practice. | ✓ | ✓ | ✓ | ✓ | ✓ | ✓ |
| Methods |  |  |  |  |  |  |  |  |
| Health economic analysis plan | 4 | Indicate whether a health economic analysis plan was developed and where available. | x | x | x | x | x | x |
| Study population | 5 | Describe characteristics of the study population (such as age range, demographics, socioeconomic, or clinical characteristics). | ✓ | ✓ | ✓ | ✓ | ✓ | ✓ |
| Setting and location | 6 | Provide relevant contextual information that may influence findings. | ✓ | ✓ | ✓ | ✓ | ✓ | ✓ |
| Comparators | 7 | Describe the interventions or strategies being compared and why chosen. | ✓ | ✓ | ✓ | ✓ | ✓ | ✓ |
| Perspective | 8 | State the perspective(s) adopted by the study and why chosen. | ✓ | ✓ | ✓ | ✓ | ✓ | ✓ |
| Time horizon | 9 | State the time horizon for the study and why appropriate. | ✓ | ✓ | ✓ | ✓ | ✓ | ✓ |
| Discount rate | 10 | Report the discount rate(s) and reason chosen. | ✓ | x | ✓ | ✓ | ✓ | ✓ |
| Selection of outcomes | 11 | Describe what outcomes were used as the measure(s) of benefit(s) and harm(s). | ✓ | N/A | ✓ | ✓ | ✓ | ✓ |
| Measurement of outcomes | 12 | Describe how outcomes used to capture benefit(s) and harm(s) were measured. | ✓ | N/A | ✓ | ✓ | ✓ | ✓ |
| Valuation of outcomes | 13 | Describe the population and methods used to measure and value outcomes. | ✓ | N/A | ✓ | ✓ | ✓ | ✓ |
| Measurement and valuation of resources and costs | 14 | Describe how costs were valued. | ✓ | ✓ | ✓ | ✓ | ✓ | ✓ |
| Currency, price date, and conversion | 15 | Report the dates of the estimated resource quantities and unit costs, plus the currency and year of conversion. | x | ✓ | ✓ | ✓ | ✓ | x |
| Rationale and description of model | 16 | If modelling is used, describe in detail and why used. Report if the model is publicly available and where it can be accessed. | ✓ | N/A | N/A | N/A | N/A | N/A |
| Analytics and assumptions | 17 | Describe any methods for analysing or statistically transforming data, any extrapolation methods, and approaches for validating any model used. | ✓ | ✓ | ✓ | ✓ | ✓ | ✓ |
| Characterising heterogeneity | 18 | Describe any methods used for estimating how the results of the study vary for subgroups. | x | x | ✓ | x | ✓ | ✓ |
| Characterising distributional effects | 19 | Describe how impacts are distributed across different individuals or adjustments made to reflect priority populations. | N/A | ✓ | ✓ | ✓ | ✓ | ✓ |
| Characterising uncertainty | 20 | Describe methods to characterise any sources of uncertainty in the analysis. | ✓ | ✓ | ✓ | ✓ | ✓ | ✓ |
| Approach to engagement with patients and others affected by the study | 21 | Describe any approaches to engage patients or service recipients, the general public, communities, or stakeholders (such as clinicians or payers) in the design of the study. | x | x | x | x | x | x |
| Results |  |  |  |  |  |  |  |  |
| Study parameters | 22 | Report all analytic inputs (such as values, ranges, references) including uncertainty or distributional assumptions. | ✓ | ✓ | ✓ | x | x | ✓ |
| Summary of main results | 23 | Report the mean values for the main categories of costs and outcomes of interest and summarise them in the most appropriate overall measure. | ✓ | ✓ | ✓ | ✓ | ✓ | ✓ |
| Effect of uncertainty | 24 | Describe how uncertainty about analytic judgments, inputs, or projections affect findings. Report the effect of choice of discount rate and time horizon, if applicable. | ✓ | ✓ | ✓ | ✓ | ✓ | ✓ |
| Effect of engagement with patients and others affected by the study | 25 | Report on any difference patient/service recipient, general public, community, or stakeholder involvement made to the approach or findings of the study | x | x | x | x | x | x |
| Discussion |  |  |  |  |  |  |  |  |
| Study findings, limitations, generalisability, and current knowledge | 26 | Report key findings, limitations, ethical or equity considerations not captured, and how these could affect patients, policy, or practice. | ✓ | ✓ | ✓ | ✓ | ✓ | ✓ |
| Other relevant information | | |  |  |  |  |  |  |
| Source of funding | 27 | Describe how the study was funded and any role of the funder in the identification, design, conduct, and reporting of the analysis | x | ✓ | ✓ | ✓ | ✓ | x |
| Conflicts of interest | 28 | Report authors conflicts of interest according to journal or International Committee of Medical Journal Editors requirements. | ✓ | ✓ | ✓ | ✓ | ✓ | ✓ |
|  |  | Successfully addressed | 21 | 19 | 24 | 22 | 23 | 22 |
|  |  | Failed to address | 6 | 5 | 3 | 5 | 4 | 5 |
|  |  | N/A | 1 | 4 | 1 | 1 | 1 | 1 |

| Table 18: Revised Cochrane risk-of-bias tool for randomized trials (RoB 2) | Sprave et al. (2020) | Smits et al. (2025) | Mir et al. (2022) | Storm et al. (2024) | Abslom et al. (2021) | Absolom et al. (2017) | Velikova et al. (2022) | Holch et al. (2018) | Maguire et al. (2017) | Kearns et al. (2022) | Breen et al. (2015) | Webb et al. (2024) | Xia et al. (2025) | Denis et al. (2017) | Wheelock t al. (2014) | Basch et al. (2016) | Billingy et al. (2021) | Dibble et al. (2025) |
| --- | --- | --- | --- | --- | --- | --- | --- | --- | --- | --- | --- | --- | --- | --- | --- | --- | --- | --- |
| 1.1 Was the allocation sequence random? | Y | Y | Y | Y | Y | Y | Y | Y | Y | Y | Y | Y | Y | Y | Y | Y | Y | Y |
| 1.2 Was the allocation sequence concealed until participants were enrolled and assigned to interventions? | PY | Y | Y | Y | Y | Y | Y | Y | Y | Y | Y | Y | Y | Y | Y | Y | Y | Y |
| 1.3 Did baseline differences between intervention groups suggest a problem with the randomization process? | NI | NI | N | NI | N | N | N | PN | PN | PN | PN | PN | PN | N | PN | N | PN | PN |
| Risk-of-bias judgement | Low | Low | Low | Low | Low | Low | Low | Low | Low | Low | Low | Low | Low | Low | Low | Low | Low | Low |
| 2.1. Were participants aware of their assigned intervention during the trial? | Y | Y | Y | Y | Y | Y | Y | Y | Y | Y | Y | Y | Y | Y | Y | Y | Y | Y |
| 2.2. Were carers and people delivering the interventions aware of participants' assigned intervention during the trial? | Y | Y | Y | Y | Y | Y | Y | Y | Y | Y | Y | Y | Y | Y | Y | Y | Y | Y |
| 2.3. If Y/PY/NI to 2.1 or 2.2: Were there deviations from the intended intervention that arose because of the trial context? | NI | NI | N | PN | Y | Y | Y | NI | NI | NI | PN | NI | PY | PY | Y | PY | PN | PN |
| 2.4 If Y/PY to 2.3: Were these deviations likely to have affected the outcome? | NI | NI | NA | PN | Y | Y | Y | NI | NI | NI | PN | NI | PY | PY | Y | PY | PN | PN |
| 2.5. If Y/PY/NI to 2.4: Were these deviations from intended intervention balanced between groups? | PY | PY | NA | PY | N | N | N | PY | NI | PY | PN | PY | NI | NI | N | NI | PY | NI |
| 2.6 Was an appropriate analysis used to estimate the effect of assignment to intervention? | Y | Y | Y | Y | Y | Y | Y | Y | Y | Y | Y | Y | Y | Y | Y | Y | Y | Y |
| 2.7 If N/PN/NI to 2.6: Was there potential for a substantial impact (on the result) of the failure to analyse participants in the group to which they were randomized? | NA | NA | N | NA | N | N | N | NA | NA | NA | NA | NA | NA | N | PN | N | NA | NA |
| Risk-of-bias judgement | SC | SC | Low | Low | HR | HR | HR | SC | SC | SC | Low | SC | HR | HR | HR | HR | Low | Low |
| 3.1 Were data for this outcome available for all, or nearly all, participants randomized? | NA | NA | Y | NA | N | N | N | NA | NA | NA | NA | NA | NA | Y | N | N | NA | NA |
| 3.2 If N/PN/NI to 3.1: Is there evidence that the result was not biased by missing outcome data? | NA | NA | Y | NA | Y | Y | Y | NA | NA | NA | NA | NA | NA | N | Y | N | NA | NA |
| 3.3 If N/PN to 3.2: Could missingness in the outcome depend on its true value? | NA | NA | PN | NA | N | N | N | NA | NA | NA | NA | NA | NA | PN | PN | Y | NA | NA |
| 3.4 If Y/PY/NI to 3.3: Is it likely that missingness in the outcome depended on its true value? | NA | NA | PN | NA | N | N | N | NA | NA | NA | NA | NA | NA | PN | PN | Y | NA | NA |
| Risk-of-bias judgement | NA | NA | Low | NA | Low | Low | Low | NA | NA | NA | NA | NA | NA | Low | Low | HR | NA | NA |
| 4.1 Was the method of measuring the outcome inappropriate? | N | N | N | N | N | N | N | N | N | N | N | N | N | N | N | Y | Y | Y |
| 4.2 Could measurement or ascertainment of the outcome have differed between intervention groups? | PN | PN | N | PN | PN | PN | PN | PN | PN | PN | PN | PN | N | N | N | N | PN | PN |
| 4.3 If N/PN/NI to 4.1 and 4.2: Were outcome assessors aware of the intervention received by study participants? | NI | N | NI | N | NI | NI | NI | NI | NI | NI | N | N | Y | NI | NI | NI | NI | PN |
| 4.4 If Y/PY/NI to 4.3: Could assessment of the outcome have been influenced by knowledge of intervention received? | PN | PN | PN | NA | PN | PN | PN | PN | PN | PN | PN | PN | PN | PN | PN | PN | PN | PN |
| 4.5 If Y/PY/NI to 4.4: Is it likely that assessment of the outcome was influenced by knowledge of intervention received? | PN | PN | PN | NA | PN | PN | PN | PN | PN | PN | PN | PN | PN | PN | PN | PN | PN | PN |
| Risk-of-bias judgement | Low | Low | Low | Low | Low | Low | Low | Low | Low | Low | Low | Low | Low | Low | Low | Low | Low | Low |
| 5.1 Were the data that produced this result analysed in accordance with a pre-specified analysis plan that was finalized before unblinded outcome data were available for analysis? | NA | NA | Y | NA | Y | Y | Y | NA | NA | NA | NA | NA | NA | NA | NI | Y | NA | NA |
| Is the numerical result being assessed likely to have been selected, on the basis of the results, from... |  |  |  |  |  |  |  |  |  |  |  |  |  |  |  |  |  |  |
| 5.2. ... multiple eligible outcome measurements (e.g. scales, definitions, time points) within the outcome domain? | NA | NA | N | NA | N | N | N | NA | NA | NA | NA | NA | NA | NA | N | N | NA | NA |
| 5.3 ... multiple eligible analyses of the data? | NA | NA | N | NA | N | N | N | NA | NA | NA | NA | NA | NA | NA | N | N | NA | NA |
| Risk-of-bias judgement | NA | NA | Low | NA | Low | Low | Low | NA | NA | NA | NA | NA | NA | NA | SC | Low | NA | NA |
| Overall risk of bias | SC | SC | Low | Low | HR | HR | HR | SC | SC | SC | Low | SC | HR | HR | HR | HR | Low | Low |

NA = not applicable, NI = not indicated, Y = yes, N = no, PY = probably yes, PN = probably no

|  | Table 19: Risk Of Bias in Non-randomized Studies – of Interventions (ROBINS-I) assessment tool | Ferre et al. (2021) | Nanton et al. (2017) | Girgis et al. (2020) | Frankland et al. (2019) | Qaderi et al. (2021) | Schougaard et al. (2016) |
| --- | --- | --- | --- | --- | --- | --- | --- |
| Bias due to confounding | |  |  |  |  |  |  |
|  | 1.1 Is there potential for confounding of the effect of intervention in this study? | N | Y | Y | Y | Y | N |
|  | **If N/PN to 1.1:** the study can be considered to be at low risk of bias due to confounding and no further signalling questions need be considered |  |  |  |  |  |  |
|  | **If Y/PY to 1.1**: determine whether there is a need to assess time-varying confounding: |  |  |  |  |  |  |
|  | 1.2. Was the analysis based on splitting participants’ follow up time according to intervention received? |  | N | N | N | N |  |
|  | **If N/PN**, answer questions relating to baseline confounding (1.4 to 1.6) |  |  |  |  |  |  |
|  | **If Y/PY**, go to question 1.3. |  |  |  |  |  |  |
|  | 1.3. Were intervention discontinuations or switches likely to be related to factors that are prognostic for the outcome? |  | NA | NA | NA | NA |  |
|  | **If N/PN**, answer questions relating to baseline confounding (1.4 to 1.6) |  | NA | NA | NA | NA |  |
|  | **If Y/PY**, answer questions relating to both baseline and time-varying confounding (1.7 and 1.8) |  | NA | NA | NA | NA |  |
|  | **Questions relating to baseline confounding only** |  |  |  |  |  |  |
|  | 1.4. Did the authors use an appropriate analysis method that controlled for all the important confounding domains? |  | Y | Y | Y | PN |  |
|  | 1.5. **If Y/PY to 1.4**: Were confounding domains that were controlled for measured validly and reliably by the variables available in this study? |  | PY | Y | Y | NI |  |
|  | 1.6. Did the authors control for any post-intervention variables that could have been affected by the intervention? |  | N | N | N | NI |  |
|  | **Questions relating to baseline and time-varying confounding** |  |  |  |  |  |  |
|  | 1.7. Did the authors use an appropriate analysis method that controlled for all the important confounding domains and for time-varying confounding? |  | NA | NA | NA | NI |  |
|  | 1.8. **If Y/PY to 1.7**: Were confounding domains that were controlled for measured validly and reliably by the variables available in this study? |  | NA | NA | NA | NI |  |
|  | **Risk of bias judgement** |  | Moderate | Low | Low | Serious |  |
| Bias in selection of participants into the study | |  |  |  |  |  |  |
|  | 2.1. Was selection of participants into the study (or into the analysis) based on participant characteristics observed after the start of intervention? |  | N | N | N | N |  |
|  | **If N/PN to 2.1:** go to 2.4 |  |  |  |  |  |  |
|  | 2.2. **If Y/PY to 2.1**: Were the post-intervention variables that influenced selection likely to be associated with intervention? |  | NA | NA | NA | NA |  |
|  | 2.3 **If Y/PY to 2.2**: Were the post-intervention variables that influenced selection likely to be influenced by the outcome or a cause of the outcome? |  | NA | NA | NA | NA |  |
|  | 2.4. Do start of follow-up and start of intervention coincide for most participants? |  | Y | Y | Y | Y |  |
|  | 2.5. **If Y/PY to 2.2 and 2.3, or N/PN to 2.4**: Were adjustment techniques used that are likely to correct for the presence of selection biases? |  | Y | Y | Y | Y |  |
|  | **Risk of bias judgement** |  | Low | Low | Low | Low |  |
| Bias in classification of interventions | |  |  |  |  |  |  |
|  | 3.1 Were intervention groups clearly defined? |  | Y | Y | Y | Y |  |
|  | 3.2 Was the information used to define intervention groups recorded at the start of the intervention? |  | Y | Y | Y | Y |  |
|  | 3.3 Could classification of intervention status have been affected by knowledge of the outcome or risk of the outcome? |  | PN | PN | N | N |  |
|  | **Risk of bias judgement** |  | Low | Low | Low | Low |  |
|  | Optional: What is the predicted direction of bias due to classification of interventions? |  |  |  |  |  |  |
| Bias due to deviations from intended interventions | |  |  |  |  |  |  |
|  | **If your aim for this study is to assess the effect of assignment to intervention, answer questions 4.1 and 4.2** |  |  |  |  |  |  |
|  | 4.1. Were there deviations from the intended intervention beyond what would be expected in usual practice? |  | PN | PY | PN | PN |  |
|  | 4.2. **If Y/PY to 4.1**: Were these deviations from intended intervention unbalanced between groups *and* likely to have affected the outcome? |  | PN | PN | PN | PN |  |
|  | **If your aim for this study is to assess the effect of starting and adhering to intervention, answer questions 4.3 to 4.6** |  |  |  |  |  |  |
|  | 4.3. Were important co-interventions balanced across intervention groups? |  | NA | NA | NA | NA |  |
|  | 4.4. Was the intervention implemented successfully for most participants? |  | NA | NA | NA | NA |  |
|  | 4.5. Did study participants adhere to the assigned intervention regimen? |  | NA | NA | NA | NA |  |
|  | 4.6. **If N/PN to 4.3, 4.4 or 4.5**: Was an appropriate analysis used to estimate the effect of starting and adhering to the intervention? |  | NA | NA | NA | NA |  |
|  | **Risk of bias judgement** |  | Low | Moderate | Low | Low |  |
| Bias due to missing data | |  |  |  |  |  |  |
|  | 5.1 Were outcome data available for all, or nearly all, participants? |  | NA | Y | N | Y |  |
|  | 5.2 Were participants excluded due to missing data on intervention status? |  | NA | N | N | N |  |
|  | 5.3 Were participants excluded due to missing data on other variables needed for the analysis? |  | NA | N | N | N |  |
|  | 5.4 **If PN/N to 5.1, or Y/PY to 5.2 or 5.3**: Are the proportion of participants and reasons for missing data similar across interventions? |  | NA | NA | Y | NA |  |
|  | 5.5 **If PN/N to 5.1, or Y/PY to 5.2 or 5.3**: Is there evidence that results were robust to the presence of missing data? |  | NA | NA | Y | NA |  |
|  | **Risk of bias judgement** |  | NA | Low | Low | Low |  |
| Bias in measurement of outcomes | |  |  |  |  |  |  |
|  | 6.1 Could the outcome measure have been influenced by knowledge of the intervention received? |  | N | N | N | N |  |
|  | 6.2 Were outcome assessors aware of the intervention received by study participants? |  | Y | Y | Y | Y |  |
|  | 6.3 Were the methods of outcome assessment comparable across intervention groups? |  | Y | Y | Y | PN |  |
|  | 6.4 Were any systematic errors in measurement of the outcome related to intervention received? |  | PN | PN | PN | NI |  |
|  | **Risk of bias judgement** |  | Low | Low | Low | Serious |  |
| Bias in selection of the reported result | |  |  |  |  |  |  |
|  | Is the reported effect estimate likely to be selected, on the basis of the results, from... |  |  |  |  |  |  |
|  | 7.1. ... multiple outcome *measurements* within the outcome domain? |  | N | N | N | NI |  |
|  | 7.2 ... multiple *analyses* of the intervention-outcome relationship? |  | N | N | N | NI |  |
|  | 7.3 ... different *subgroups*? |  | N | N | N | NI |  |
|  | **Risk of bias judgement** |  |  |  |  |  |  |
|  | Optional: What is the predicted direction of bias due to selection of the reported result? |  | Low | Low | Low | Serious |  |
| Overall bias | |  |  |  |  |  |  |
|  | **Risk of bias judgement** | Low | Moderate | Moderate | Low | Serious | Low |

NA = not applicable, NI = not indicated, Y = yes, N = no, PY = probably yes, PN = probably no

| Table 20: Mixed Methods Appraisal Tool | Schmalz et al. (2020) |
| --- | --- |
| Screening questions (for all types) |  |
| S1. Are there clear research questions? | Y |
| S2. Do the collected data allow to address the research questions? | Y |
| 1. Qualitative |  |
| 1.1. Is the qualitative approach appropriate to answer the research question? | Y |
| 1.2. Are the qualitative data collection methods adequate to address the research question? | Y |
| 1.3. Are the findings adequately derived from the data? | Y |
| 1.4. Is the interpretation of results sufficiently substantiated by data? | Y |
| 1.5. Is there coherence between qualitative data sources, collection, analysis and interpretation? | Y |
| 2. Quantitative RCTs |  |
| 2.1. Is randomization appropriately performed? | NA |
| 2.2. Are the groups comparable at baseline? | NA |
| 2.3. Are there complete outcome data? | NA |
| 2.4. Are outcome assessors blinded to the intervention provided? | NA |
| 2.5 Did the participants adhere to the assigned intervention? | NA |
| Quantitative NRCTs |  |
| 3.1. Are the participants representative of the target population? | NA |
| 3.2. Are measurements appropriate regarding both the outcome and intervention (or exposure)? | NA |
| 3.3. Are there complete outcome data? | NA |
| 3.4. Are the confounders accounted for in the design and analysis? | NA |
| 3.5. During the study period, is the intervention administered (or exposure occurred) as intended? | NA |
| 4. Quantitative descriptive |  |
| 4.1. Is the sampling strategy relevant to address the research question? | Y |
| 4.2. Is the sample representative of the target population? | Y |
| 4.3. Are the measurements appropriate? | N |
| 4.4. Is the risk of nonresponse bias low? | Y |
| 4.5. Is the statistical analysis appropriate to answer the research question? | N |
| 5. Mixed methods |  |
| 5.1. Is there an adequate rationale for using a mixed methods design to address the research question? | Y |
| 5.2. Are the different components of the study effectively integrated to answer the research question? | Y |
| 5.3. Are the outputs of the integration of qualitative and quantitative components adequately interpreted? | Y |
| 5.4. Are divergences and inconsistencies between quantitative and qualitative results adequately addressed? | NA |
| 5.5. Do the different components of the study adhere to the quality criteria of each tradition of the methods involve | Y |
| Yes | 14 |
| No | 2 |
| NA | 11 |

NA = not applicable, NI = not indicated, Y = yes, N = no

| Table 21: Preferred Reporting Items for Systematic reviews and Meta-Analyses extension for Scoping Reviews (PRISMA-ScR) Checklist | | | Aapro et al. (2020) |
| --- | --- | --- | --- |
| Title | 1 | Identify the report as a scoping review. | Y |
| ABSTRACT | | |  |
| Structured summary | 2 | Provide a structured summary that includes (as applicable): background, objectives, eligibility criteria, sources of evidence, charting methods, results, and conclusions that relate to the review questions and objectives. | Y |
| INTRODUCTION | | |  |
| Rationale | 3 | Describe the rationale for the review in the context of what is already known. Explain why the review questions/objectives lend themselves to a scoping review approach. | Y |
| Objectives | 4 | Provide an explicit statement of the questions and objectives being addressed with reference to their key elements (e.g., population or participants, concepts, and context) or other relevant key elements used to conceptualize the review questions and/or objectives. | Y |
| METHODS | | |  |
| Protocol and registration | 5 | Indicate whether a review protocol exists; state if and where it can be accessed (e.g., a Web address); and if available, provide registration information, including the registration number. | N |
| Eligibility criteria | 6 | Specify characteristics of the sources of evidence used as eligibility criteria (e.g., years considered, language, and publication status), and provide a rationale. | Y |
| Information sources* | 7 | Describe all information sources in the search (e.g., databases with dates of coverage and contact with authors to identify additional sources), as well as the date the most recent search was executed. | Y |
| Search | 8 | Present the full electronic search strategy for at least 1 database, including any limits used, such that it could be repeated. | Y |
| Selection of sources of evidence† | 9 | State the process for selecting sources of evidence (i.e., screening and eligibility) included in the scoping review. | Y |
| Data charting process‡ | 10 | Describe the methods of charting data from the included sources of evidence (e.g., calibrated forms or forms that have been tested by the team before their use, and whether data charting was done independently or in duplicate) and any processes for obtaining and confirming data from investigators. | Y |
| Data items | 11 | List and define all variables for which data were sought and any assumptions and simplifications made. | Y |
| Critical appraisal of individual sources of evidence§ | 12 | If done, provide a rationale for conducting a critical appraisal of included sources of evidence; describe the methods used and how this information was used in any data synthesis (if appropriate). | Y |
| Synthesis of results | 13 | Describe the methods of handling and summarizing the data that were charted. | Y |
| RESULTS | | |  |
| Selection of sources of evidence | 14 | Give numbers of sources of evidence screened, assessed for eligibility, and included in the review, with reasons for exclusions at each stage, ideally using a flow diagram. | Y |
| Characteristics of sources of evidence | 15 | For each source of evidence, present characteristics for which data were charted and provide the citations. | Y |
| Critical appraisal within sources of evidence | 16 | If done, present data on critical appraisal of included sources of evidence (see item 12). | NA |
| Results of individual sources of evidence | 17 | For each included source of evidence, present the relevant data that were charted that relate to the review questions and objectives. | Y |
| Synthesis of results | 18 | Summarize and/or present the charting results as they relate to the review questions and objectives. | Y |
| DISCUSSION | | |  |
| Summary of evidence | 19 | Summarize the main results (including an overview of concepts, themes, and types of evidence available), link to the review questions and objectives, and consider the relevance to key groups. | Y |
| Limitations | 20 | Discuss the limitations of the scoping review process. | N |
| Conclusions | 21 | Provide a general interpretation of the results with respect to the review questions and objectives, as well as potential implications and/or next steps. | Y |
| FUNDING | | |  |
| Funding | 22 | Describe sources of funding for the included sources of evidence, as well as sources of funding for the scoping review. Describe the role of the funders of the scoping review. | Y |

NA = not applicable, NI = not indicated, Y = yes, N = no

# Appendix references

1.	Aapro M, Bossi P, Dasari A, et al. Digital health for optimal supportive care in oncology: benefits, limits, and future perspectives. *Support Care Cancer*. 2020;28(10). doi:10.1007/s00520-020-05539-1

2.	Qaderi SM, Swartjes H, Vromen H, Bremers AJA, Custers JAE, de Wilt JHW. Acceptability, quality of life and cost overview of a remote follow-up plan for patients with colorectal cancer. *Eur J Surg Oncol*. 2021;47(7). doi:10.1016/j.ejso.2020.12.018

3.	Schougaard LMV, Larsen LP, Jessen A, et al. AmbuFlex: tele-patient-reported outcomes (telePRO) as the basis for follow-up in chronic and malignant diseases. *Qual Life Res*. 2016;25:525-534. doi:10.1007/s11136-015-1207-0

4.	Sprave T, Zöller D, Stoian R, et al. App-controlled treatment monitoring and support for head and neck cancer patients (APCOT): Protocol for a prospective randomized controlled trial. *JMIR Res Protoc*. 2020;9(12). doi:10.2196/21693

5.	Smits MJM, Bolman CAW, Mesters I, Lechner L. Blended Care Intervention for Cancer Aftercare in General Practice Centers: Protocol for a Randomized Controlled Trial. *JMIR Res Protoc*. 2025;14(101599504):e64662. doi:10.2196/64662

6.	Mir O, Ferrua M, Fourcade A, et al. Digital remote monitoring plus usual care versus usual care in patients treated with oral anticancer agents: the randomized phase 3 CAPRI trial. *Nat Med*. 2022;28(6):1224-1231. doi:10.1038/s41591-022-01788-1

7.	Minvielle E, Leleu H, Masseti M, et al. A budget impact analysis of a digital monitoring solution in patients treated with oral anticancer agents: a medico-economic analysis of the randomized phase 3 CAPRI trial. *Eur J Health Econ*. 2025;26(2):267-273. doi:10.1007/s10198-024-01702-1

8.	Storm M, Morken IM, Austin RC, et al. Evaluation of the nurse-assisted eHealth intervention ‘eHealth@Hospital-2-Home’ on self-care by patients with heart failure and colorectal cancer post-hospital discharge: protocol for a randomised controlled trial. *BMC Health Serv Res*. 2024;24(1). doi:10.1186/s12913-023-10508-5

9.	Absolom K, Holch P, Warrington L, et al. Electronic patient self-Reporting of adverse-events: Patient information and advice (eRAPID): A randomised controlled trial in systemic cancer treatment. *BMC Cancer*. 2017;17(1). doi:10.1186/s12885-017-3303-8

10.	Holch P, Pini S, Henry AM, et al. ERAPID electronic patient self-Reporting of Adverse-events: Patient Information and aDvice: A pilot study protocol in pelvic radiotherapy. *Pilot Feasibility Stud*. 2018;4(1). doi:10.1186/s40814-018-0304-6

11.	Absolom K, Warrington L, Hudson E, et al. Phase III Randomized Controlled Trial of eRAPID: eHealth Intervention During Chemotherapy. *J Clin Oncol*. Published online January 8, 2021. doi:10.1200/JCO.20.02015

12.	Velikova G, Absolom K, Hewison J, et al. Electronic self-reporting of adverse events for patients undergoing cancer treatment: the eRAPID research programme including two RCTs. Published online 2022. doi:10.3310/FDDE8516

13.	Dawkins B, Absolom K, Hewison J, et al. Cost-Effectiveness of eRAPID eHealth Intervention for Symptom Management During Chemotherapy. *JCO Oncol Pract*. 2024;20(4):581-590. doi:10.1200/OP.23.00498

14.	Maguire R, Fox PA, McCann L, et al. The eSMART study protocol: A randomised controlled trial to evaluate electronic symptom management using the advanced symptom management system (ASyMS) remote technology for patients with cancer. *BMJ Open*. 2017;7(5). doi:10.1136/bmjopen-2016-015016

15.	Ferre F., de Rosis S., Murante A.M., et al. Systematic and continuous collection of patient-reported outcomes and experience in women with cancer undergoing mastectomy and immediate breast reconstruction: a study protocol for the Tuscany Region (Italy). *BMJ Open*. 2021;11(1):234901.

16.	Nanton V, Appleton R, Dale J, et al. Integrated care in prostate cancer (ICARE-P): Nonrandomized controlled feasibility study of online holistic needs assessment, linking the patient and the health care team. *JMIR Res Protoc*. 2017;6(7). doi:10.2196/resprot.7667

17.	Schmalz O, Jacob C, Ammann J, et al. Digital monitoring and management of patients with advanced or metastatic non-small cell lung cancer treated with cancer immunotherapy and its impact on quality of clinical care: Interview and survey study among health care professionals and patients. *J Med Internet Res*. 2020;22(12). doi:10.2196/18655

18.	Kearns N, Raigal-Aran L, O’Connell K, et al. The Women’s Health Initiative cancer survivorship clinic incorporating electronic patient-reported outcomes: a study protocol for the Linking You to Support and Advice (LYSA) randomized controlled trial. *Pilot Feasibility Stud*. 2022;8(1). doi:10.1186/s40814-022-01186-x

19.	Koumakis L, Schera F, Parker H, et al. Fostering Palliative Care Through Digital Intervention: A Platform for Adult Patients With Hematologic Malignancies. *Front Digit Health*. 2021;3. doi:10.3389/fdgth.2021.730722

20.	Crafoord MT, Ekstrand J, Sundberg K, Nilsson MI, Fjell M, Langius-Eklöf A. Mobile Electronic Patient-Reported Outcomes and Interactive Support During Breast and Prostate Cancer Treatment: Health Economic Evaluation From Two Randomized Controlled Trials. *JMIR Cancer*. 2025;11:e53539. doi:10.2196/53539

21.	Breen S, Ritchie D, Schofield P, et al. The Patient Remote Intervention and Symptom Management System (PRISMS) - a Telehealth- mediated intervention enabling real-time monitoring of chemotherapy side-effects in patients with haematological malignancies: Study protocol for a randomised controlled trial. *Trials*. 2015;16(1). doi:10.1186/s13063-015-0970-0

22.	Webb PM, Brown A, Brown B, et al. Patient Reported Outcome Measures in cancer care: a hybrid effectiveness-Implementation trial to optimise Symptom control and health service Experience (PROMISE)–protocol for a randomised controlled trial of electronic self-reporting of symptoms versus usual care during and following treatment in... *BMJ Open*. 2024;14(11). doi:10.1136/bmjopen-2024-090836

23.	Girgis A, Durcinoska I, Arnold A, et al. Web-Based Patient-Reported Outcome Measures for Personalized Treatment and Care (PROMPT-Care): Multicenter Pragmatic Nonrandomized Trial. *J Med Internet Res*. 2020;22(10):e19685. doi:10.2196/19685

24.	Xia Y, Guan X, Shi Z, Luo Y, He P. Remote symptom monitoring with patient-reported outcomes and nudges during lung cancer immunotherapy in China (PRO-NET): Protocol for a randomised controlled trial. *BMJ Open*. 2025;15(1). doi:10.1136/bmjopen-2024-093374

25.	Denis F, Lethrosne C, Pourel N, et al. Randomized Trial Comparing a Web-Mediated Follow-up With Routine Surveillance in Lung Cancer Patients. *J Natl Cancer Inst*. 2017;109(9). doi:10.1093/jnci/djx029

26.	Lizee T, Basch E, Tremolieres P, et al. Cost-Effectiveness of Web-Based Patient-Reported Outcome Surveillance in Patients With Lung Cancer. *J Thorac Oncol Off Publ Int Assoc Study Lung Cancer*. 2019;14(6):1012-1020. doi:10.1016/j.jtho.2019.02.005

27.	Wheelock AE, Bock MA, Martin EL, et al. SIS.NET: a randomized controlled trial evaluating a web-based system for symptom management after treatment of breast cancer. *Cancer*. 2015;121(6):893-899. doi:10.1002/cncr.29088

28.	Basch E, Deal AM, Kris MG, et al. Symptom Monitoring With Patient-Reported Outcomes During Routine Cancer Treatment: A Randomized Controlled Trial. *J Clin Oncol Off J Am Soc Clin Oncol*. 2016;34(6):557-565. doi:10.1200/JCO.2015.63.0830

29.	Nixon NA, Spackman E, Clement F, Verma S, Manns B. Cost-effectiveness of symptom monitoring with patient-reported outcomes during routine cancer treatment. *J Cancer Policy*. 2018;15. doi:10.1016/j.jcpo.2017.12.001

30.	Billingy NE, Tromp VNMF, Veldhuijzen E, et al. SYMptom monitoring with Patient-Reported Outcomes using a web application among patients with Lung cancer in the Netherlands (SYMPRO-Lung): Study protocol for a stepped-wedge randomised controlled trial. *BMJ Open*. 2021;11(9). doi:10.1136/bmjopen-2021-052494

31.	Frankland J, Brodie H, Cooke D, et al. Follow-up care after treatment for prostate cancer: Evaluation of a supported self-management and remote surveillance programme. *BMC Cancer*. 2019;19(1). doi:10.1186/s12885-019-5561-0

32.	van Egdom LSE, Lagendijk M, van der Kemp MH, et al. Implementation of Value Based Breast Cancer Care. *Eur J Surg Oncol*. 2019;45(7). doi:10.1016/j.ejso.2019.01.007

33.	Dibble KE, Rosenberg SM, Snow C, et al. Young, empowered & strong (YES): a study protocol paper for a randomized controlled trial of an mhealth symptom monitoring and self-management intervention for adolescent and young adult (AYA) breast cancer survivors. *BMC Public Health*. 2025;25(126). doi:10.1186/s12889-025-21288-4

34.	Nixon N.A., Spackman E., Clement F., Verma S., Manns B. Cost-effectiveness of symptom monitoring with patient-reported outcomes during routine cancer treatment. *J Cancer Policy*. 2018;15((Nixon, Verma) Tom Baker Cancer Centre, 1331 29th St., NW Calgary, AB, Canada):32 EP - 36. doi:10.1016/j.jcpo.2017.12.001

35.	Exchange Rates UK: Compare Best Live Rates, News & Forecasts. Accessed March 10, 2026. https://www.exchangerates.org.uk/

36.	Inflation calculator. Accessed March 10, 2026. https://www.bankofengland.co.uk/monetary-policy/inflation/inflation-calculator

37.	Absolom K, Warrington L, Hudson E, et al. Data Supplement for Phase III Randomised Controlled Trial of eRAPID: eHealth Intervention During Chemotherapy. *J Clin Oncol*. Published online November 3, 2025. doi:10.1200/JCO.20.02015

38.	Higgins JPT, Altman DG, Gøtzsche PC, et al. The Cochrane Collaboration’s tool for assessing risk of bias in randomised trials. *The BMJ*. 2011;343:d5928. doi:10.1136/bmj.d5928

39.	Husereau D, Drummond M, Augustovski F, et al. Consolidated Health Economic Evaluation Reporting Standards 2022 (CHEERS 2022) Statement: Updated Reporting Guidance for Health Economic Evaluations. *Value Health J Int Soc Pharmacoeconomics Outcomes Res*. 2022;25(1):3-9. doi:10.1016/j.jval.2021.11.1351

40.	Sterne JA, Hernán MA, Reeves BC, et al. ROBINS-I: a tool for assessing risk of bias in non-randomised studies of interventions. *BMJ*. 2016;355:i4919. doi:10.1136/bmj.i4919

41.	National Institute for Health and Care Excellence. Appendix H: Appraisal checklists, evidence tables, GRADE and economic profiles. Accessed February 11, 2026. https://www.nice.org.uk/process/pmg20/resources/appendix-h-appraisal-checklists-evidence-tables-grade-and-economic-profiles-pdf-8779777885

42.	Government Social Research Profession and Cabinet Office. Quality in Qualitative Evaluation: A framework for assessing research evidence. GOV.UK. 2003. Accessed February 20, 2026. https://www.gov.uk/government/publications/government-social-research-framework-for-assessing-research-evidence

43.	Hong QN, Gonzalez-Reyes A, Pluye P. Improving the usefulness of a tool for appraising the quality of qualitative, quantitative and mixed methods studies, the Mixed Methods Appraisal Tool (MMAT). *J Eval Clin Pract*. 2018;24(3):459-467. doi:10.1111/jep.12884

44.	Tricco AC, Lillie E, Zarin W, et al. PRISMA Extension for Scoping Reviews (PRISMA-ScR): Checklist and Explanation. *Ann Intern Med*. 2018;169(7):467-473. doi:10.7326/M18-0850

45.	Singh S, Upadhyaya S, Deshmukh P, et al. Time motion study using mixed methods to assess service delivery by frontline health workers from South India: methods. *Hum Resour Health*. 2018;16(1):17. doi:10.1186/s12960-018-0279-7

46.	Maguire R, Kotronoulas G, Donnan PT, et al. Development and preliminary testing of a brief clinical tool to enable daily monitoring of chemotherapy toxicity: The Daily Chemotherapy Toxicity self-Assessment Questionnaire. *Eur J Cancer Care (Engl)*. 2018;27(6):e12890. doi:10.1111/ecc.12890

47.	van der Hout A, Jansen F, van Uden-Kraan CF, et al. Cost-utility of an eHealth application “Oncokompas” that supports cancer survivors in self-management: results of a randomised controlled trial. *J Cancer Surviv*. 2021;15(1):77-86. doi:10.1007/s11764-020-00912-9

48.	Denis F, Lethrosne C, Pourel N, et al. Randomized Trial Comparing a Web-Mediated Follow-up With Routine Surveillance in Lung Cancer Patients. *J Natl Cancer Inst*. 2017;109(9). doi:10.1093/jnci/djx029

49.	Basch E, Artz D, Dulko D, et al. Patient online self-reporting of toxicity symptoms during chemotherapy. *J Clin Oncol Off J Am Soc Clin Oncol*. 2005;23(15):3552-3561. doi:10.1200/JCO.2005.04.275
